# Supplementary material for: Brain rhythms define distinct interaction networks with differential dependence on anatomy
Source: Neuron. 2021 Dec 1;109(23):3862–3878.e5. doi: 10.1016/j.neuron.2021.09.052 (PMC8639786; doi:10.1016/j.neuron.2021.09.052)
Supplement: Document S1. Figures S1–S8 and Tables S1 and S2 [file mmc1.pdf]

Neuron, Volume *109*

Supplemental information

Brain rhythms define distinct interaction networks with differential dependence on anatomy

Julien Vezoli, Martin Vinck, Conrado Arturo Bosman, André Moraes Bastos, Christopher Murphy Lewis, Henry Kennedy, and Pascal Fries

Fig.S1

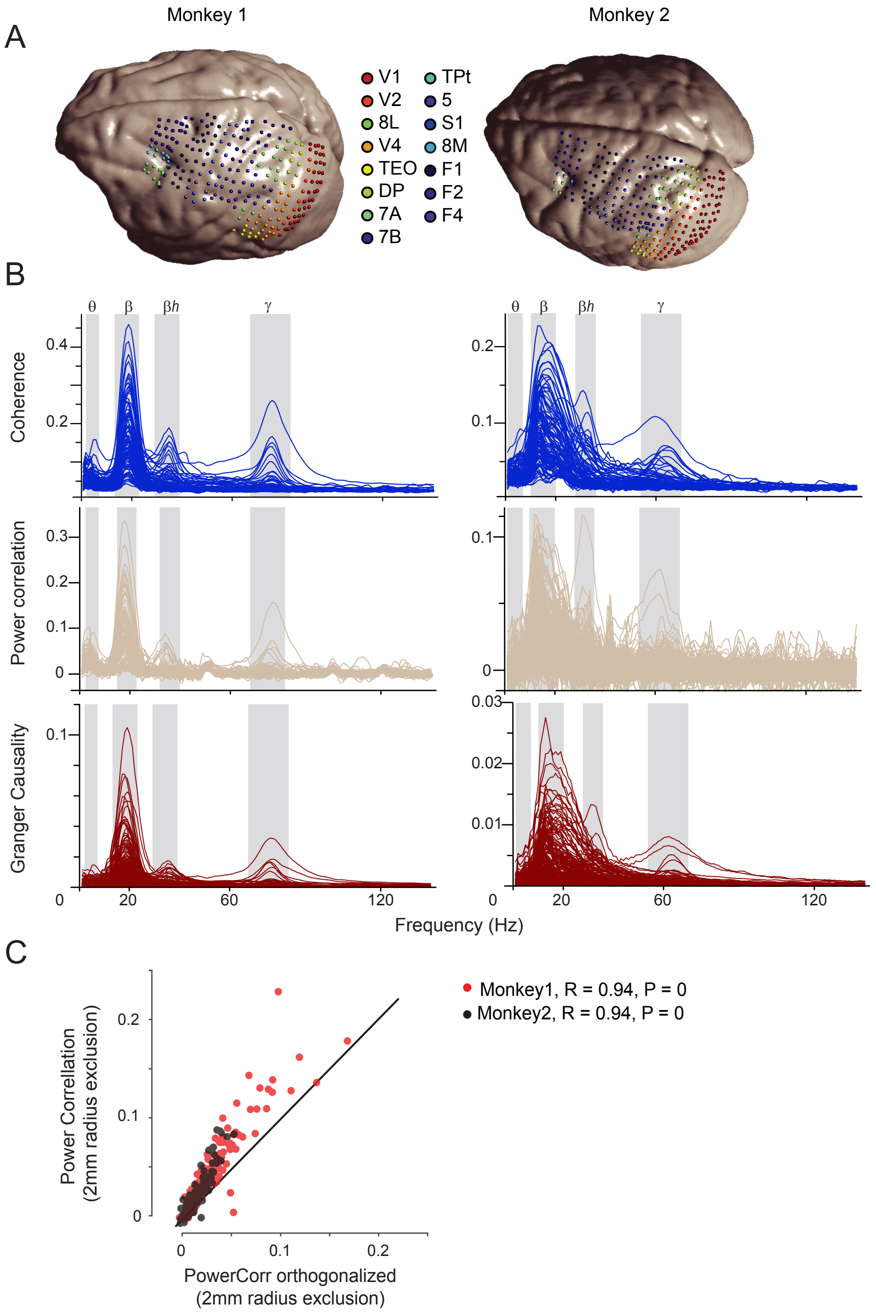

Figure S1. Recording site distributions and FC spectra per monkey, orthogonalized power correlation. Related to Figure 1.

(**A**) ECoG grids registered to brain surface reconstructions from individual MRIs of the two macaque monkeys, as indicated. Sites are colored according to the area color legend on the right, based on the Kennedy lab nomenclature (Markov et al., 2011). V1: Primary visual cortex; V2: Secondary visual cortex; 8L: Lateral part of area 8/FEF; V4: Fourth visual area; TEO: Temporal-occipital area; DP: Dorsal prelunate area; 7A and 7B: Parts A and B of parietal area 7; TPt: temporo-parietal area (posterior auditory association cortex); 5: area 5; S1: Primary somatosensory cortex; 8M: Medial part of area 8/FEF; F1: corresponding to Primary motor cortex; F2: corresponding to the caudal part of dorsal premotor cortex; F4: corresponding to the caudal part ventral premotor cortex.

(**B**) Same as Fig. 1C, but including power correlation and GC spectra. Spectra from Monkey 1 are in the left column, those of Monkey 2 in the right column. The coherence spectra in this figure are identical to those in Fig. 1C and merely included for completeness.

(**C**) Scatter plot of power correlation versus orthogonalized power correlation. Each dot corresponds to one pair of recording sites in Monkey 1 (red dots) or Monkey 2 (black dots), excluding sites within a 2 mm radius to avoid residual volume conduction effects. Note that power correlation and orthogonalized power correlation values were highly correlated (R=0.94, P=0) in both monkeys.

Fig.S2

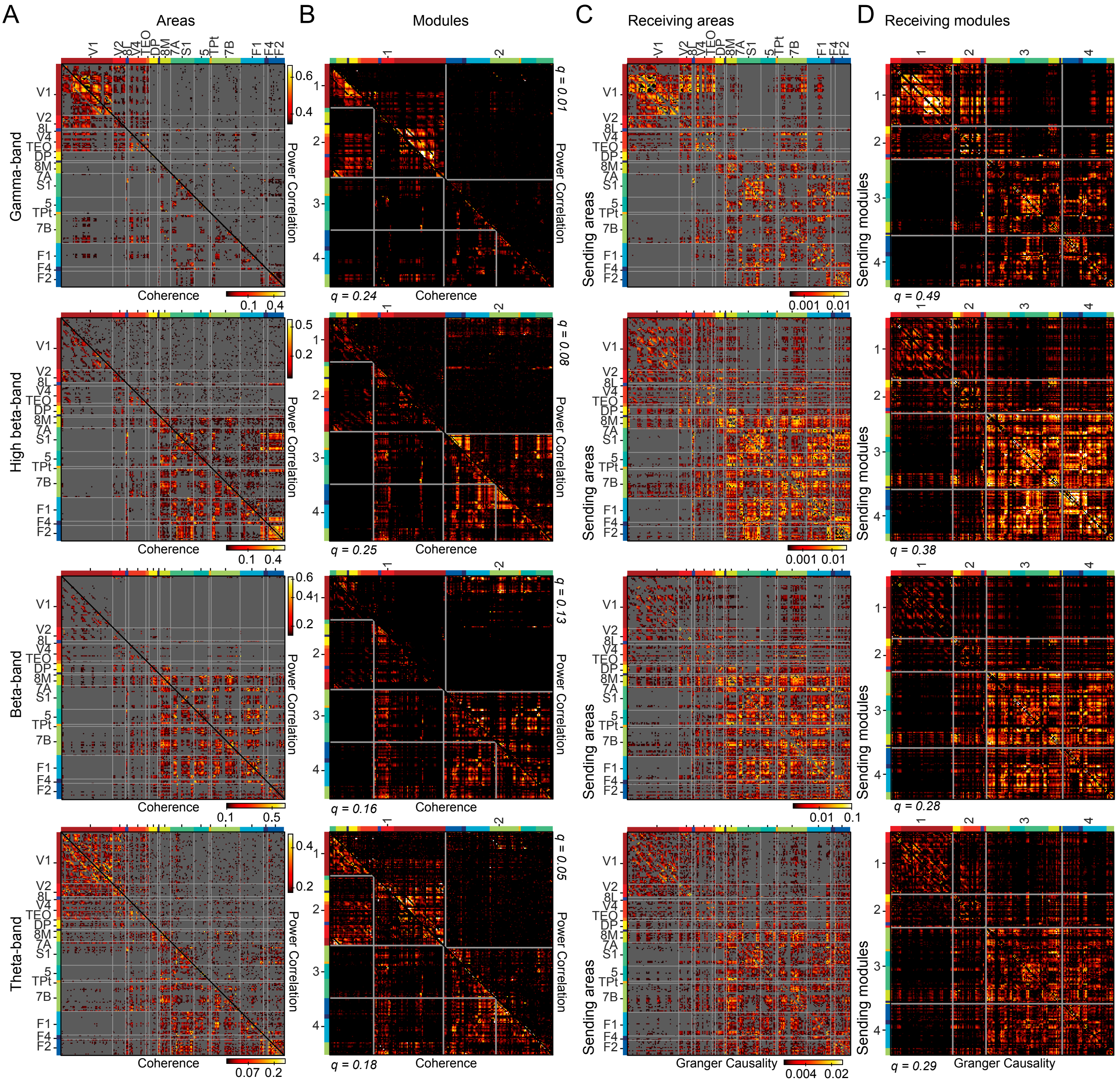

Figure S2. FC matrices for Monkey 2. Related to Figure 2.
(**A-D**) Same as Fig. 2 A-D, but for Monkey 2.

Fig.S3

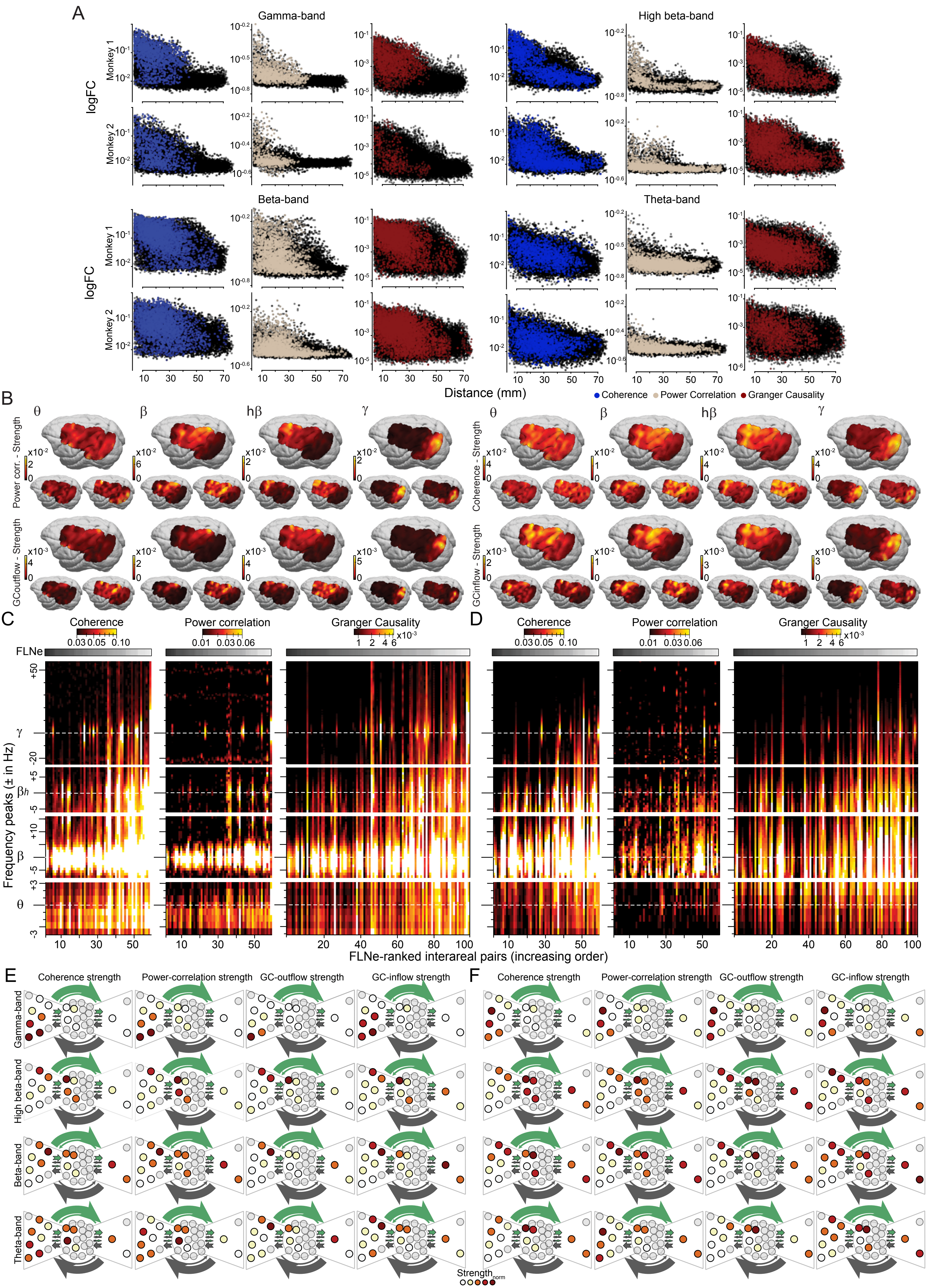
A
Gamma-band
High beta-band
Beta-band
Theta-band
logFC
Monkey 1
Monkey 2
Distance (mm)
Coherence
Power Correlation
Granger Causality
B
Power corr. - Strength
GCoutflow - Strength
Coherence - Strength
GCinflow - Strength
C
D
Coherence
Power correlation
Granger Causality
FLNe
Frequency peaks (± in Hz)
FLNe-ranked interareal pairs (increasing order)
E
F
Coherence strength
Power-correlation strength
GC-outflow strength
GC-inflow strength
Gamma-band
High beta-band
Beta-band
Theta-band
Strength norm

Figure S3. Per monkey: Site-pair-wise FC versus distance, FC strength topographies, FLNe-ranked FC spectra, and FC strength displayed on the AC-derived core-periphery structure. Related to Figure 3 and Figure 8.

(A) Scatter plots of FC versus distance on the cortical surface (each dot corresponds to one pair of recording sites from separate cortical areas), separately per FC type (color code on bottom right), per frequency band (as indicated above the four quadrants of the panel), and per monkey (as indicated for odd versus even rows). Black dots indicate lack of significance (comparison to a random graph with equal weight distribution; FDR-corrected for multiple comparisons over sites, see Methods).

(B) Same analyses as in Fig. 3, but showing data for the average over both monkeys (larger brains) and separately per monkey (smaller brains: Monkey 1 on left, Monkey 2 on right). To allow optimal comparison, significance masking was removed. Analyses were performed separately per FC type (as indicated to the left of the four quadrants of the panel), and per frequency band (as indicated on the upper left corner of each column).

(C) Same as left part of Fig. S4A, but for Monkey 1.

(D) Same as left part of Fig. S4A, but for Monkey 2.

(E) Same as Fig. 8, but for Monkey 1.

(F) Same as Fig. 8, but for Monkey 2.

Fig.S4

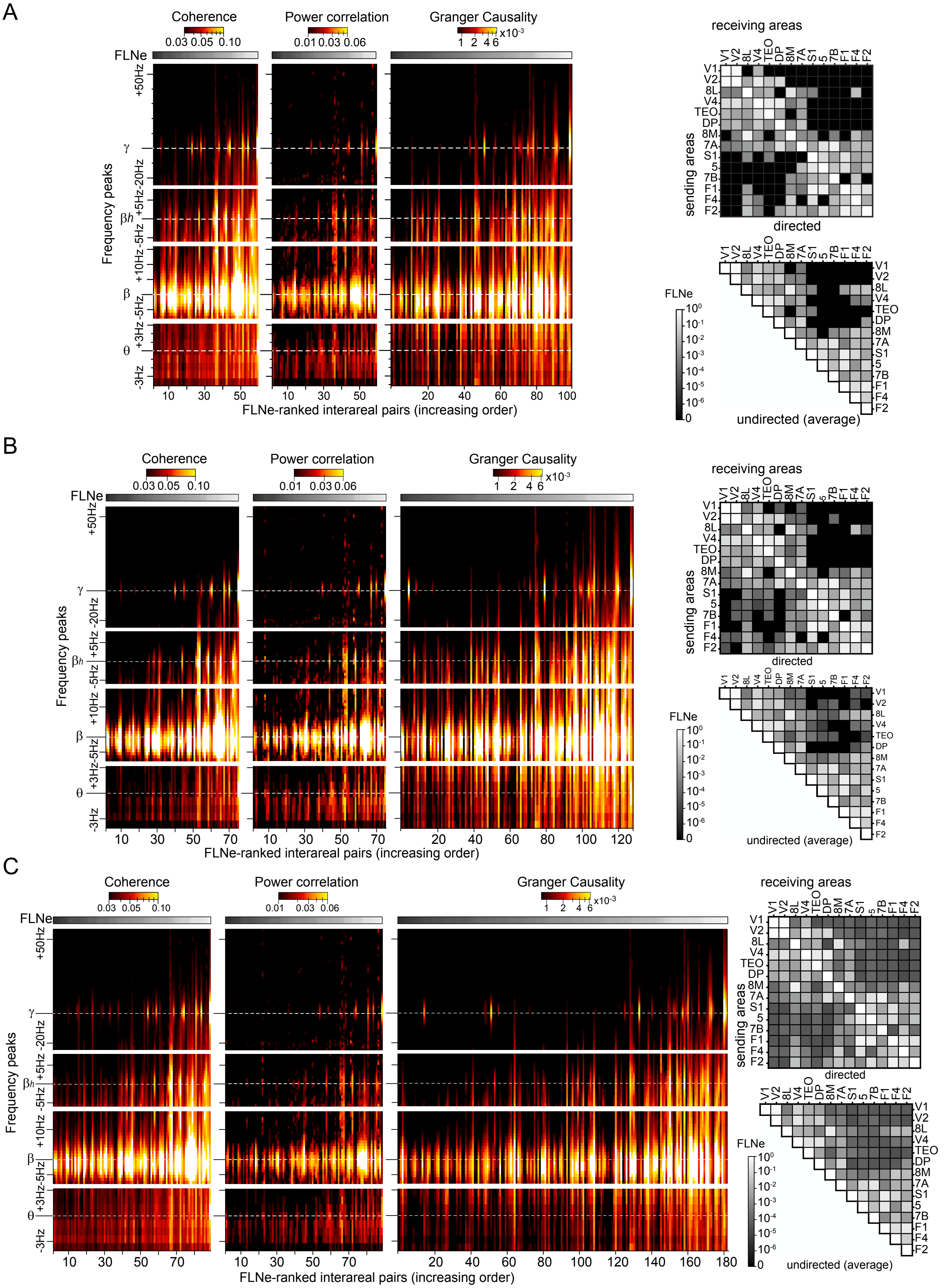

Figure S4. FLNe and FLNe-sorted FC spectra. Related to Figure 4.
(**A**) Coherence, power correlation and GC values (color coded) as a function of frequency (y-axis, relative to per-monkey band-wise peak frequencies) and as a function of FLNe rank (x-axis) of the respective interareal pair. For coherence and power correlation, ranking used FLNe averaged over the two directions as shown in the triangular matrix on the bottom right of the panel. For GC, ranking used FLNe as shown in the full matrix on the top right of the panel. FC values were first averaged over all respective interareal site pairs and subsequently over animals.
(**B**) Same as (A), but including FLNe values based on less than 10 neurons, while still excluding FLNe values of zero. The corresponding FLNe matrices are shown on the right.
(**C**) Same as (A), but including FLNe values based on less than 10 neurons, and replacing FLNe values of zero by estimates assuming Poisson distributions fitted to neuron counts from non-zero FLNe distributions. The corresponding FLNe matrices are shown on the right.

Fig.S5

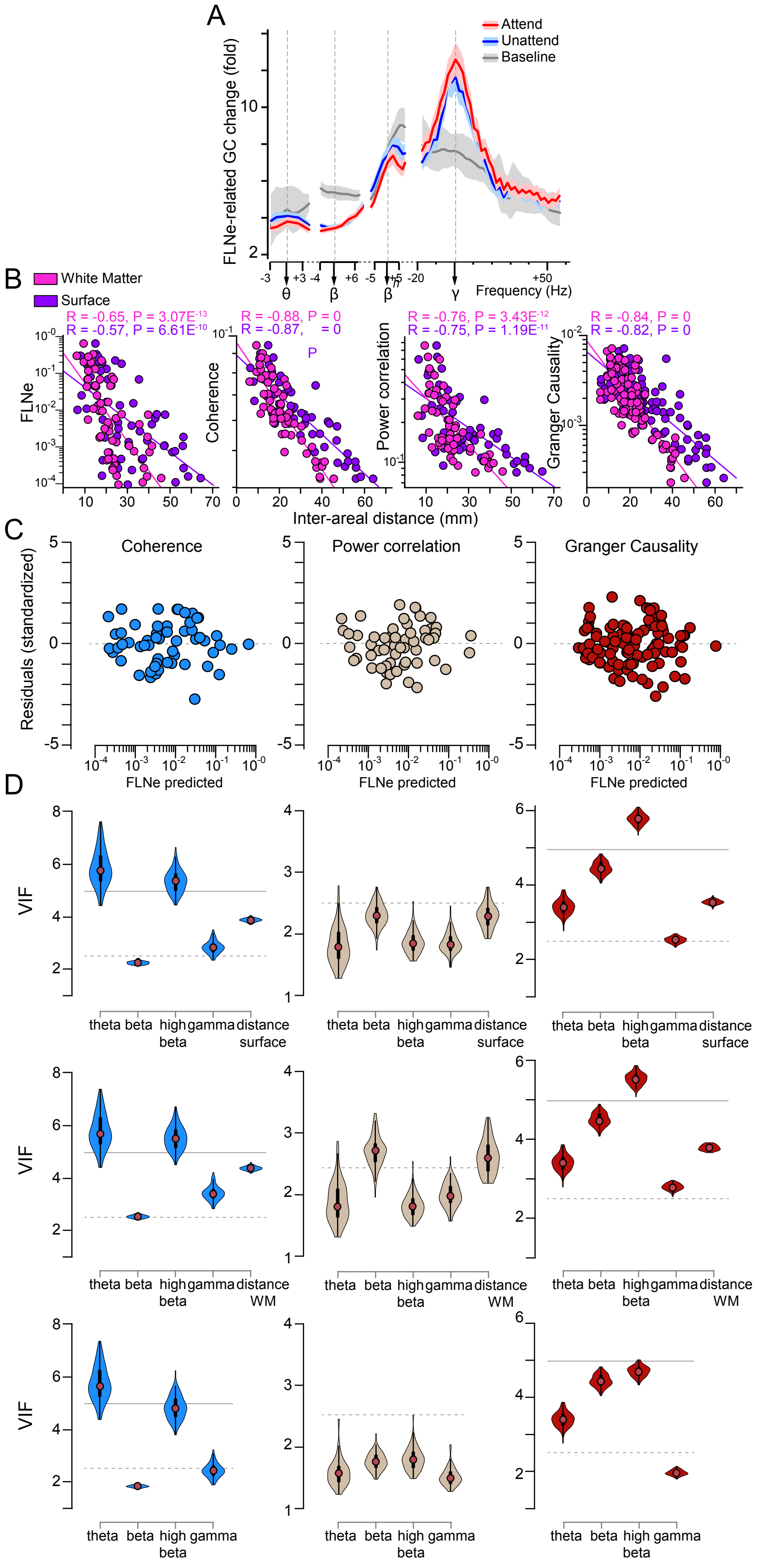

Figure S5. AC-FC correlation for attention conditions and baseline; AC and FC correlate with interareal distance. Related to Figure 5.

(**A**) Same as the FLNe-related GC change shown as red line in Fig. 5C, but here separately for the two attention conditions and the baseline period, as indicated in the color legend on the top right of the panel. Spectra show means over all trials ± 99.9% confidence intervals from bootstrap estimates over trials.

(**B**) Linear regression of log10(FLNe), log10(Coherence), log10(Power correlation) and log10(Granger Causality) against interareal distance on the brain surface (purple) or through white-matter (pink). For FLNe, each dot corresponds to an interareal projection with more than 10 labeled neurons (N=100), for coherence and power correlation, each dot corresponds to a pair of cortical areas (N=105), for GC, each dot corresponds to a directed interareal influence (N=210). FC values were averaged over the four individual frequency bands and over the two monkeys. Individual decay rates are presented in Table S1.

(**C**) Scatterplots of residuals against predicted variable verify homoscedasticity for each FC metric.

(**D**) Variance Inflation Factor (VIF) calculated for each predicting variable of the three models, with interareal distance on the brain surface (top) or through white-matter (WM, middle) as an additional predicting variable or not (bottom).

Dashed (VIF = 2.5) and plain lines (VIF = 5) show moderate correlation range still below critical level.

Fig.S6

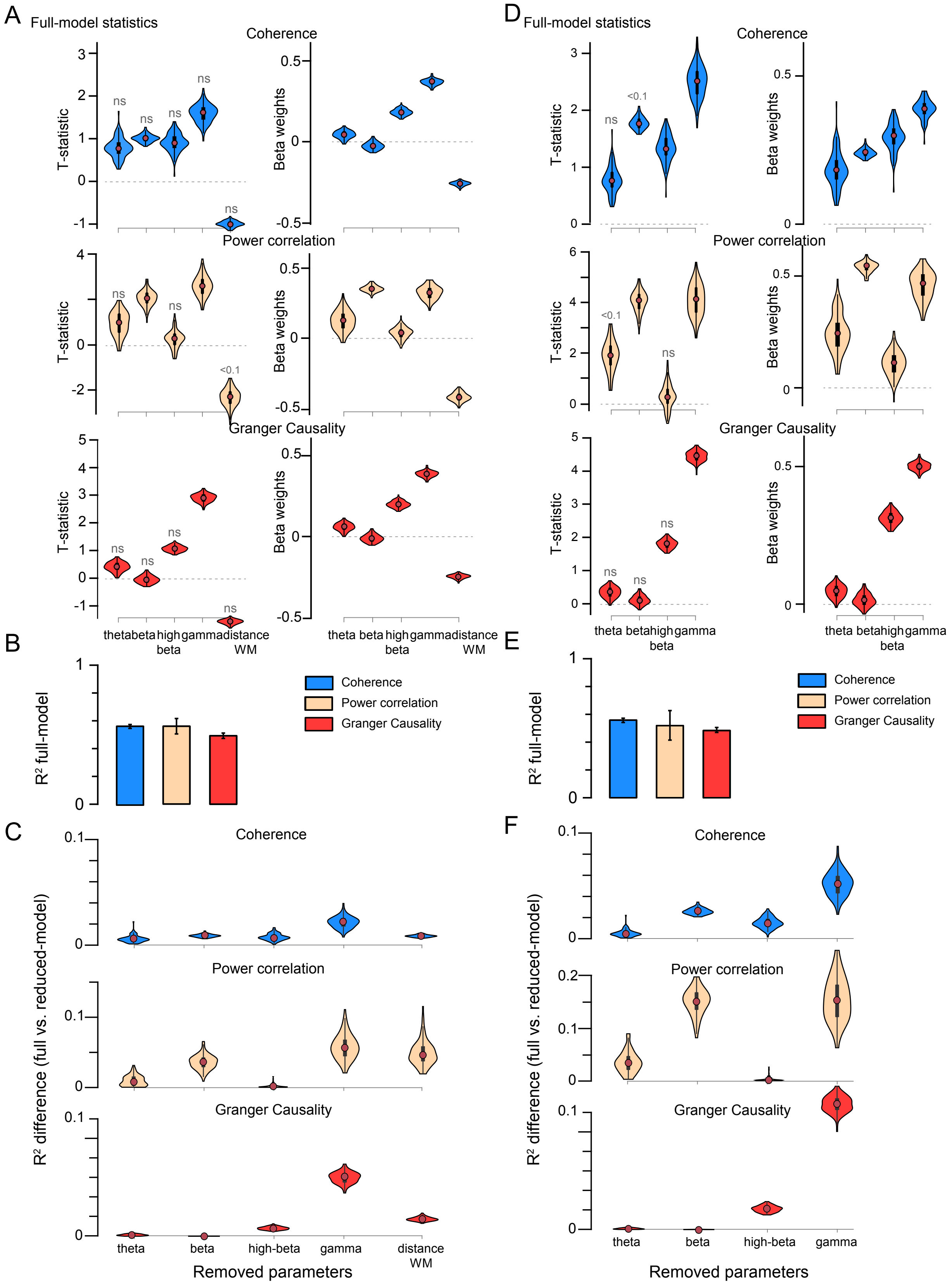

Figure S6. Multiple linear regression with or without interareal distance as additional predictor. Related to Figure 6.
(**A-C**) Same as Figure 6A-C, but using interareal distance through white-matter (WM).
(**D-F**) Same as Fig.6A-C, but not including interareal distance as predicting variable.
In (B) and (E), means and ± 99.9% confidence intervals from bootstrap estimates over trials.

Fig.S7

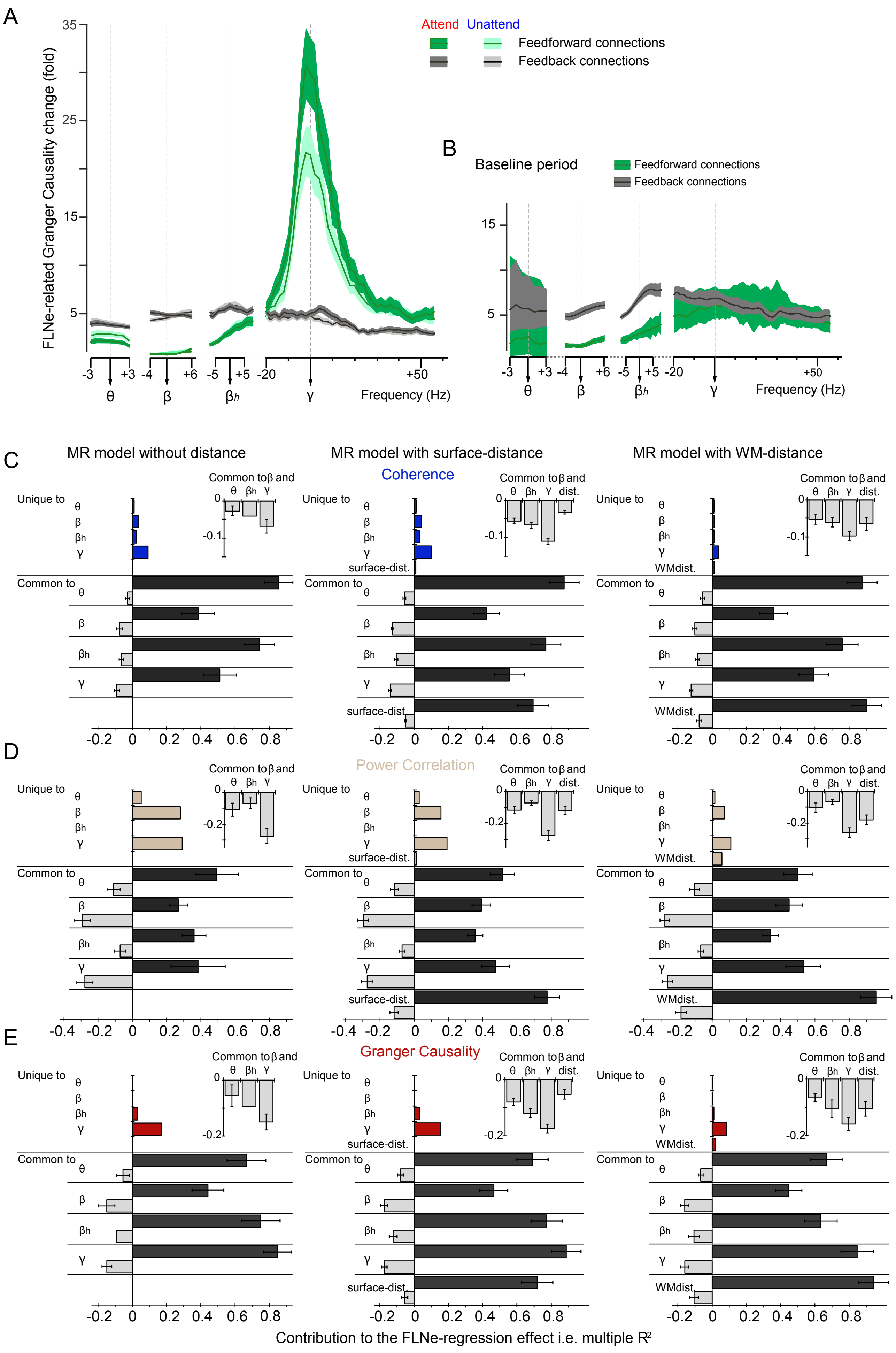
A
Attend Unattend
Feedforward connections
Feedback connections
FLNe-related Granger Causality change (fold)
35
25
15
5
-3 +3 -4 +6 -5 +5 -20 +50
θ β βh γ
Frequency (Hz)
B
Baseline period
Feedforward connections
Feedback connections
15
5
Frequency (Hz)
C
MR model without distance
MR model with surface-distance
MR model with WM-distance
Coherence
Unique to
Common to
Common to β and
θ βh γ dist.
surface-dist.
WMdist.
D
Power Correlation
E
Granger Causality
-0.4 -0.2 0 0.2 0.4 0.6 0.8
Contribution to the FLNe-regression effect i.e. multiple R²

Figure S7. AC-FC correlation split by SLN and attention conditions; Commonality analysis summary. Related to Figure 7.

(A) Same as Fig. 7A (main panel), but separately for the two attention conditions indicated at the top.

(B) Same as Fig. 7A (main panel), but for the Baseline period.

(C-E) Unique (*Direct*) and Common (*Shared*) effects for all predictor variables as proportion of the regression effect (i.e. multiple R^2) for MR models with and without distance as additional regressor and for the three FC metrics: Coherence (C), Power Correlations (D), Granger Causality (E). Common (*Shared*) effects are summed separately for positive and negative effects. Inset displays summed Common negative effects for all pairs of predictors involving beta-band synchronizations, Sum ± SD.

Results demonstrate that FC in the gamma band is the dominant variable, with the strongest direct effect and the highest relative importance in MR models predicting FLNe, with or without partial regression of distance. Commonality analysis identified a substantial proportion of the regression effect caused by suppression in MR models, consistently involving FC in the beta-frequency band (sum of negative shared coefficients for Coherence, Power Correlation and GC: 9.73%-29.4%-15.03% without distance, 17.64%-29.75%-17.79% with surface-distance and 12.97%-27.74%-15.98% with WM-distance). The amount of overall suppression caused by beta-band FC in the model was strongest when combined with FC in the gamma-frequency band (insets Fig.S7C-E) suggesting subtle involvement of cross-frequency interactions in the observed regression effect. Furthermore, in all models, we observed that beta-band FC contributed the least to the MR effect (i.e. lowest rs^2). Hence, even if poorly predictive of FLNe, the main contribution of beta-band FC to the MR effect was to increase FLNe-predictive power of the remaining variables and mostly for gamma-band FC.

Spectra in (A-B) show means over all trials ± 99.9% confidence intervals from bootstrap estimates over trials. (C-E) Mean ± SEM.

Fig.S8

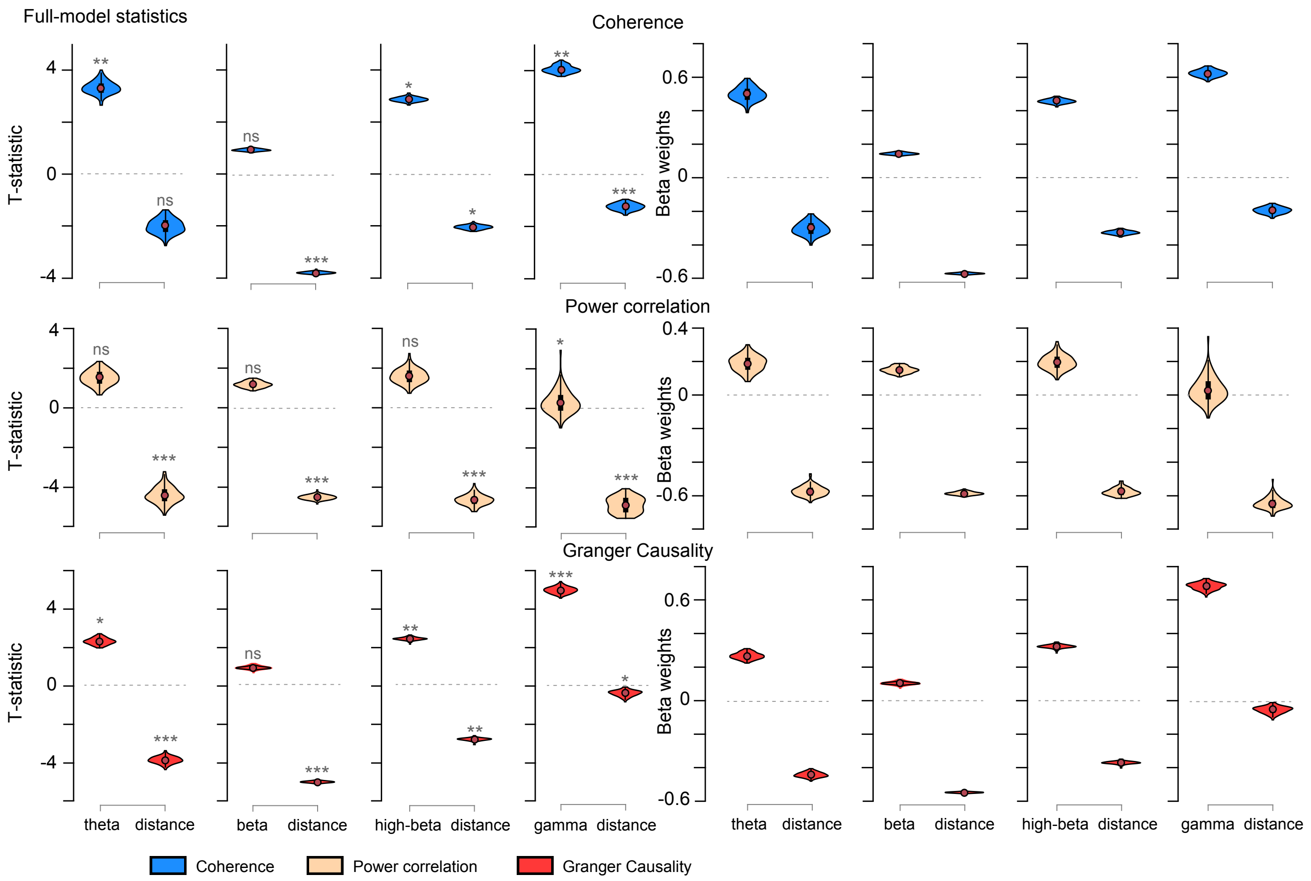

Figure S8. Multiple regression per frequency band. Related to Figure 6.
Similar analysis as Fig. 6, showing T-statistics and Beta weights of multiple linear regressions, with the dependent variable being log10(FLNe), and the independent variables being distance (on the cortical surface) and log10(FC), but now separately for theta, beta, high-beta and gamma, as indicated on the x-axes. The different FC types are shown in the four rows.

TableS1. Exponential decay rates of FC measures with interareal distance.

Mean over all trials ± SEM estimated from bootstrap over trials.

	Interareal distance	Coherence	Power Correlations	Granger Causality
Theta	WM	0.024 ± 0.0005 mm^{-1}	0.028 ± 0.004 mm^{-1}	0.055 ± 0.0012 mm^{-1}
	surface	0.016 ± 0.004 mm^{-1}	0.018 ± 0.003 mm^{-1}	0.034 ± 0.0008 mm^{-1}
Beta	WM	0.033 ± 0.0003 mm^{-1}	0.032 ± 0.001 mm^{-1}	0.063 ± 0.0007 mm^{-1}
	surface	0.021 ± 0.0002 mm^{-1}	0.019 ± 0.001 mm^{-1}	0.039 ± 0.0005 mm^{-1}
High-Beta	WM	0.042 ± 0.0004 mm^{-1}	0.028 ± 0.003 mm^{-1}	0.085 ± 0.0008 mm^{-1}
	surface	0.027 ± 0.0003 mm^{-1}	0.018 ± 0.002 mm^{-1}	0.055 ± 0.0005 mm^{-1}
Gamma	WM	0.027 ± 0.0004 mm^{-1}	0.015 ± 0.002 mm^{-1}	0.075 ± 0.0008 mm^{-1}
	surface	0.018 ± 0.0003 mm^{-1}	0.010 ± 0.001 mm^{-1}	0.049 ± 0.0006 mm^{-1}

TableS2. Summary statistics of multiple regression models for FLNe.

Relative importance measures (Commonality Analysis: Direct and Shared coefficients, General Dominance – GenDom., adjusted Effect Size and Relative Importance Weights – RIW) performed on the mean over all trials; otherwise, mean ± 99.9% CI estimated from bootstrap over trials.

Coherence

Predictors	R^2	R_{adj}^2	NRMSE	r_s^2	Beta values	Beta weights	sig.	Direct	Shared	GenDom.	Effect size	RIW
	0.563 ± 0.02	0.522 ± 0.02	0.23 ± 0.002									
Theta				0.83 ± 0.08	1.73 ± 1.65	0.20 ± 0.19	ns	0.0068	0.4606	0.153	0.136	0.138
Beta				0.34 ± 0.02	1.33 ± 0.26	0.28 ± 0.05	< 0.1	0.0241	0.1914	0.066	0.105	0.079
High-beta				0.69 ± 0.03	1.66 ± 0.75	0.34 ± 0.15	ns	0.0183	0.3919	0.125	0.203	0.123
Gamma				0.51 ± 0.04	2.23 ± 0.53	0.43 ± 0.10	0.0165	0.0562	0.2851	0.123	0.213	0.144
Surface dist.				0.65 ± 0.02	0.01 ± .003	0.17 ± 0.04	ns	0.0056	0.3678	0.096	-0.093	0.080
	0.569 ± 0.01	0.528 ± 0.01	0.23 ± 0.002									
Theta				0.83 ± 0.08	1.58 ± 1.59	0.19 ± 0.19	ns	0.0057	0.4618	0.144	0.123	0.133
Beta				0.34 ± 0.02	0.76 ± 0.22	0.16 ± 0.04	ns	0.0064	0.1850	0.057	0.057	0.068
High-beta				0.69 ± 0.03	1.04 ± 0.76	0.21 ± 0.15	ns	0.0061	0.3858	0.115	0.119	0.114
Gamma				0.50 ± 0.04	1.52 ± 0.57	0.29 ± 0.11	ns	0.0206	0.2645	0.104	0.141	0.123
WM dist.				0.84 ± 0.02	-0.02 ± .005	-0.21 ± 0.04	ns	0.0072	0.4674	0.146	0.126	0.128
	0.558 ± 0.01	0.525 ± 0.02	0.23 ± 0.002									
Theta				0.84 ± 0.08	1.56 ± 1.60	0.18 ± 0.19	ns	0.0055	0.4619	0.184	0.121	0.162
Beta				0.34 ± 0.02	-1.14 ± 0.22	0.24 ± 0.04	< 0.1	0.0188	0.1726	0.080	0.086	0.091
High-beta				0.70 ± 0.03	1.44 ± 0.75	0.29 ± 0.15	ns	0.0137	0.3782	0.152	0.166	0.146
Gamma				0.51 ± 0.04	2.02 ± 0.53	0.39 ± 0.10	0.02	0.0511	0.2340	0.142	0.186	0.160

Table S2 (continued). Summary statistics of multiple regression models for FLNe.
Mean over all trials ± 99.9% CI estimated from bootstrap over trials.

Power correlations

Predictors	R^2	R_{adj}^2	NRMSE	r_s^2	Beta values	Beta weights	sig.	Direct	Shared	GenDom.	Effect size	RIW
	0.545 ± 0.08	0.502 ± 0.08	0.23 ± 0.01									
Theta				0.43 ± 0.28	0.83 ± 1.21	0.19 ± 0.24	ns	0.0164	0.2193	0.092	0.086	0.094
Beta				0.25 ± 0.07	2.03 ± 0.75	0.49 ± 0.18	0.0028	0.0859	0.0525	0.107	0.167	0.118
High-beta				0.29 ± 0.18	0.05 ± 0.86	0.01 ± 0.20	ns	0.0001	0.1565	0.051	0.004	0.049
Gamma				0.39 ± 0.14	2.44 ± 1.06	0.49 ± 0.21	0.0011	0.1051	0.1103	0.149	0.210	0.159
Surface dist.				0.67 ± 0.10	-0.01 ± 0.01	-0.14 ± 0.18	ns	0.0070	0.3608	0.146	0.079	0.125
	0.571 ± 0.05	0.529 ± 0.06	0.23 ± 0.007									
Theta				0.41 ± 0.26	0.61 ± 1.15	0.14 ± 0.23	ns	0.0087	0.2770	0.086	0.063	0.089
Beta				0.24 ± 0.06	-1.50 ± 0.77	0.36 ± 0.18	0.0316	0.043	0.0981	0.089	0.124	0.100
High-beta				0.27 ± 0.17	0.11 ± 0.73	0.03 ± 0.17	ns	0.0004	0.1562	0.050	0.010	0.049
Gamma				0.38 ± 0.14	1.97 ± 1.00	0.40 ± 0.20	0.0085	0.0618	0.1536	0.129	0.170	0.139
WM dist.				0.83 ± 0.08	-0.03 ± 0.02	-0.32 ± 0.17	< 0.1	0.0321	0.4425	0.216	0.204	0.194
	0.539 ± 0.10	0.504 ± 0.11	0.23 ± 0.01									
Theta				0.39 ± 0.26	1.25 ± 1.32	0.24 ± 0.26	< 0.1	0.0281	0.2076	0.125	0.105	0.122
Beta				0.27 ± 0.08	2.34 ± 0.71	0.56 ± 0.16	0.0001	0.1516	-0.0132	0.151	0.191	0.155
High-beta				0.31 ± 0.19	0.15 ± 0.94	0.03 ± 0.22	ns	0.0003	0.1563	0.000	0.010	0.062
Gamma				0.36 ± 0.14	2.70 ± 1.11	0.55 ± 0.21	0.0001	0.1574	0.0580	0.157	0.233	0.199

Table S2 (continued). Summary statistics of multiple regression models for FLNe.
Mean over all trials ± 99.9% CI estimated from bootstrap over trials.

Granger Causality

Predictors	R^2	R_{adj}^2	NRMSE	r_s^2	Beta values	Beta weights	sig.	Direct	Shared	GenDom.	Effect size	RIW
	0.485 ± 0.02	0.458 ± 0.02	0.23 ± 0.02									
Theta				0.61 ± 0.07	0.24 ± 0.27	0.07 ± 0.07	ns	0.0009	0.2963	0.079	0.037	0.073
Beta				0.29 ± 0.03	-0.09 ± 0.17	-0.04 ± 0.07	ns	0.0002	0.1412	0.039	-0.014	0.039
High-beta				0.68 ± 0.03	0.78 ± 0.20	0.36 ± 0.08	< 0.1	0.0162	0.3162	0.100	0.191	0.090
Gamma				0.87 ± 0.03	1.17 ± 0.17	0.53 ± 0.06	0.0004	0.0751	0.3465	0.183	0.316	0.199
Surface dist.				0.67 ± 0.02	-0.01 ± 0.003	0.09 ± 0.05	ns	0.0016	0.3220	0.085	-0.045	0.084
	0.492 ± 0.01	0.465 ± 0.01	0.23 ± 0.001									
Theta				0.60 ± 0.07	0.19 ± 0.29	0.06 ± 0.08	ns	0.0005	0.2967	0.074	0.030	0.069
Beta				0.29 ± 0.02	-0.08 ± 0.17	-0.03 ± 0.07	ns	0.0002	0.1414	0.037	-0.012	0.036
High-beta				0.67 ± 0.03	0.44 ± 0.16	0.20 ± 0.06	ns	0.0052	0.3324	0.092	0.108	0.083
Gamma				0.86 ± 0.03	0.89 ± 0.17	0.40 ± 0.06	0.007	0.0414	0.4216	0.160	0.240	0.178
WM dist.				0.85 ± 0.02	-0.02 ± 0.004	-0.21 ± 0.04	ns	0.0086	0.4202	0.130	0.127	0.126
	0.484 ± 0.02	0.462 ± 0.02	0.23 ± 0.002									
Theta				0.61 ± 0.07	0.24 ± 0.27	0.07 ± 0.07	ns	0.0009	0.2963	0.097	0.038	0.087
Beta				0.29 ± 0.03	-0.09 ± 0.17	-0.04 ± 0.07	ns	0.0002	0.1412	0.048	-0.014	0.045
High-beta				0.69 ± 0.03	0.68 ± 0.19	0.31 ± 0.07	ns	0.0148	0.3176	0.125	0.165	0.111
Gamma				0.87 ± 0.03	1.09 ± 0.15	0.49 ± 0.05	0.0002	0.0831	0.3385	0.214	0.295	0.240
