# Supplementary material for: Brain rhythms define distinct interaction networks with differential dependence on anatomy
Source: Neuron. 2021 Dec 1;109(23):3862–3878.e5. doi: 10.1016/j.neuron.2021.09.052 (PMC8639786; doi:10.1016/j.neuron.2021.09.052)
Supplement: Document S2. Article plus supplemental information [file mmc2.pdf]

Neuron

Article

Brain rhythms define distinct interaction networks with differential dependence on anatomy

Highlights

- Coherence, power correlation, and Granger causality among >200 sites across 15 areas
- These interaction metrics peak at the theta, beta, high-beta, and gamma rhythms
- The 4 rhythms define distinct interaction networks, largely independent of power
- The networks differentially depend on anatomy, strongly for gamma, weakly for beta

Authors

Julien Vezoli, Martin Vinck, Conrado Arturo Bosman, André Moraes Bastos, Christopher Murphy Lewis, Henry Kennedy, Pascal Fries

Correspondence

julien.vezoli@esi-frankfurt.de (J.V.), pascal.fries@esi-frankfurt.de (P.F.)

In brief

The brain is much more than the sum of its parts because those parts interact in complex networks. Vezoli et al. show that at least 4 distinct interaction networks coexist, mediated by the neuronal synchronization of 4 brain rhythms. These rhythm-defined networks are differentially dependent on the strengths of anatomical projections.

Vezoli et al., 2021, Neuron *109*, 3862–3878
December 1, 2021 © 2021 The Authors. Published by Elsevier Inc.
https://doi.org/10.1016/j.neuron.2021.09.052

CellPress
OPEN ACCESS

Neuron

Article

Brain rhythms define distinct interaction networks with differential dependence on anatomy

Julien Vezoli,[1,*] Martin Vinck,[1,8] Conrado Arturo Bosman,[2,3] André Moraes Bastos,[1,4] Christopher Murphy Lewis,[1,5] Henry Kennedy,[6,7] and Pascal Fries[1,2,9,*]

[1]Ernst Strüngmann Institute (ESI) for Neuroscience in Cooperation with the Max Planck Society, 60528 Frankfurt, Germany
[2]Donders Institute for Brain, Cognition, and Behaviour, Radboud University, 6525 EN Nijmegen, the Netherlands
[3]Swammerdam Institute for Life Sciences, Center for Neuroscience, Faculty of Science, University of Amsterdam, 1098 XH Amsterdam, the Netherlands
[4]Department of Psychology and Vanderbilt Brain Institute, Vanderbilt University, Nashville, TN 37240, USA
[5]Brain Research Institute, University of Zurich, Zurich 8057, Switzerland
[6]Université Lyon, Université Claude Bernard Lyon 1, Inserm, Stem Cell and Brain Research Institute U1208, 69500 Bron, France
[7]Institute of Neuroscience, State Key Laboratory of Neuroscience, Chinese Academy of Sciences (CAS) Key Laboratory of Primate Neurobiology, Shanghai 200031, China
[8]Donders Centre for Neuroscience, Department of Neuroinformatics, Radboud University, 6525 AJ Nijmegen, the Netherlands
[9]Lead contact
*Correspondence: julien.vezoli@esi-frankfurt.de (J.V.), pascal.fries@esi-frankfurt.de (P.F.)
https://doi.org/10.1016/j.neuron.2021.09.052

SUMMARY

Cognitive functions are subserved by rhythmic neuronal synchronization across widely distributed brain areas. In 105 area pairs, we investigated functional connectivity (FC) through coherence, power correlation, and Granger causality (GC) in the theta, beta, high-beta, and gamma rhythms. Between rhythms, spatial FC patterns were largely independent. Thus, the rhythms defined distinct interaction networks. Importantly, networks of coherence and GC were not explained by the spatial distributions of the strengths of the rhythms. Those networks, particularly the GC networks, contained clear modules, with typically one dominant rhythm per module. To understand how this distinctiveness and modularity arises on a common anatomical backbone, we correlated, across 91 area pairs, the metrics of functional interaction with those of anatomical projection strength. Anatomy was primarily related to coherence and GC, with the largest effect sizes for GC. The correlation differed markedly between rhythms, being less pronounced for the beta and strongest for the gamma rhythm.

INTRODUCTION

Cognitive functions emerge in distributed neuronal networks through local and interareal neuronal interactions, constituting a complex interaction network. A full account of this interaction network will be fundamental for understanding brain function. Neuronal interaction networks depend on structural neuronal connectivity networks, and central insights have been obtained from anatomy. Anatomical tract tracing based on tracer injections in animals has revealed that connection strengths decrease exponentially with distance and are highly structured with characteristic motifs (Ercsey-Ravasz et al., 2013; Horvát et al., 2016; Theodoni et al., 2021). While anatomical connectivity (AC) is necessary for neuronal interaction, it is not identical to it. A given anatomical projection may or may not be used for neuronal interactions at a given moment, and it may be used for neuronal interactions of different kinds (e.g., mediating activation, suppression, modulation). While AC is typically measured in regard to monosynaptic connections, interareal neuronal interactions can extend to di- and polysynaptic interactions. While much of anatomical tract tracing, including the data used here, is focused on cortico-cortical connections, neuronal interactions may also use subcortical pathways (Guillery and Sherman, 2002).

Interareal neuronal interactions are subserved by neuronal entrainment and synchronization (Bosman et al., 2012; Brovelli et al., 2004; Gregoriou et al., 2009; Grothe et al., 2012; Lobier et al., 2018; Siegel et al., 2008). Interareal neuronal synchronization can be assessed by local field potential (LFP) coherence, and entrainment can be assessed by LFP Granger causality (GC). Both coherence and GC can be determined per frequency, resulting in coherence or GC spectra. Also, neuronal interactions can lead to correlated power fluctuations, which can be assessed by power correlation spectra. Intriguingly, neuronal rhythms in different frequency bands mediate different types of interareal interactions. We showed previously that among 8 macaque visual areas, interareal GC is stronger in the bottom-up direction for theta and gamma, and stronger in the top-down direction for beta (Bastos et al., 2015b). This raises the

 © 2021 The Authors. Published by Elsevier Inc.
This is an open access article under the CC BY license (http://creativecommons.org/licenses/by/4.0/).

CellPress
OPEN ACCESS

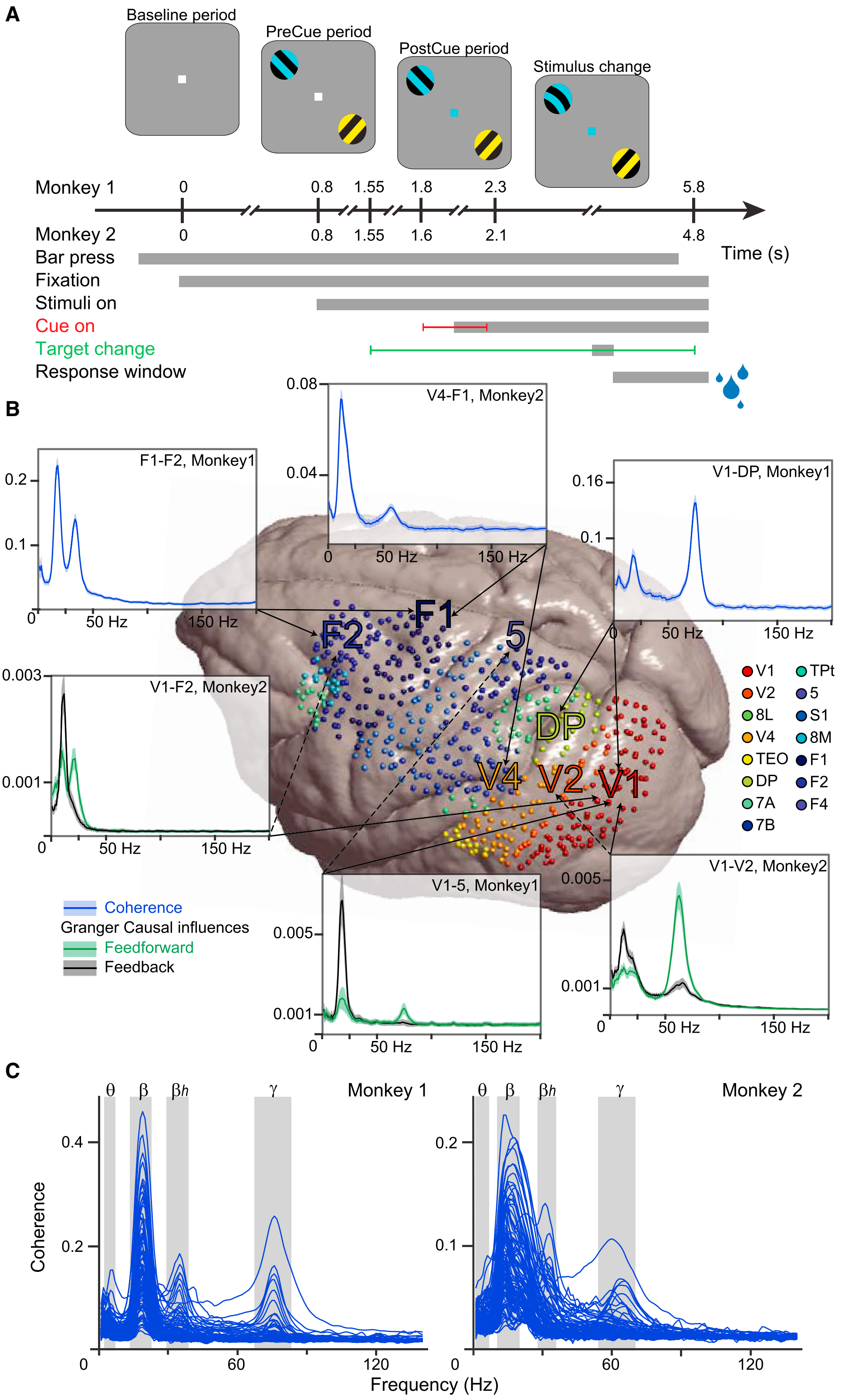

(legend on next page)

possibility that different rhythms define distinct interaction networks, including coherence and GC networks, as also suggested by previous analyses of power correlations in human subjects (Brookes et al., 2011; de Pasquale et al., 2010; de Pasquale et al., 2012; Hipp et al., 2012; Hipp and Siegel, 2015). Our previous analysis only took into account the GC asymmetry between bottom-up and top-down directions, per area pair, thereby accounting for only a very small component of the total GC variability. We related these GC asymmetries to differences in the laminar pattern between anatomical bottom-up and top-down projections, per area pair, again accounting for merely a tiny part of the total variability in anatomical projections. Anatomical projection strengths show a much larger variance, in fact, >5 orders of magnitude, across different area pairs (Markov et al., 2014a). Here, we establish the full variability in interareal power correlation, coherence, and GC across all pairs of simultaneously recorded sites and brain areas, which we directly relate to the full variability in interareal anatomical projection strength across those area pairs.

We use a unique high-resolution micro-electrocorticography (mECoG) dataset providing simultaneous LFP signals from 218 recording sites distributed across 15 areas, in 2 awake macaques. The complete 218 × 218 matrices of power correlation, coherence, and GC revealed the respective interaction networks to consist of clearly defined modules, and that the coherence and GC networks are independent of the underlying power distributions. Intriguingly, those interaction networks agree partly for some pairs of frequency bands, while differing markedly between others, as observed in human subjects (Williams et al., 2021). This is remarkable given that all rhythms operate on the same anatomical backbone, suggesting that they are differentially affected by AC. To understand this better, we analyzed the mutual dependence between on the one hand, interareal power correlation, coherence, or GC, and on the other hand, the strength of the corresponding anatomical projections. Anatomical projection strength was assessed by retrograde tracer injections and quantification of labeled neurons in many cortical areas. Across area pairs, the resulting cortico-cortical projection strengths predicted power correlation less than coherence, and they were most predictive of GC. Importantly, anatomical projection strengths predicted coherence and GC much better in the gamma band than in the beta band, with intermediate values for the high-beta band. Finally, as we had previously found that beta is stronger in the top-down and gamma stronger in the bottom-up direction, we reanalyzed the correlation between anatomical projection and functional interaction strength, independently for the 2 directions. This showed that variability in beta-based interactions was more related to projections in the top-down direction, and variability in gamma-based interactions to projections in the bottom-up direction. These findings provide a fuller account of cortical interaction networks defined by brain rhythms and reveal a previously unsuspected richer landscape.

RESULTS

Interareal functional connectivity (FC) occurs in 4 characteristic frequency bands

We investigated neuronal activity in large-scale brain networks in 2 macaque monkeys performing a selective visual attention task (Figure 1A; see Method details). We focused on the task period with sustained visual stimulation and attention. Attention conditions were pooled to increase sensitivity, except where explicitly noted. Chronically implanted subdural mECoG grids with 252 electrodes allowed simultaneous recording from 218 local bipolar derivations, referred to as (recording) sites, distributed over large parts of the left hemisphere (Figure 1B for the combined sites of both monkeys, Figure S1A for the sites per monkey), and covering 15 cortical areas. We computed the following frequency-resolved FC metrics between all possible site pairs: (1) coherence, a metric of interareal synchronization; (2) power correlation, the Spearman rank correlation between fluctuations in band-limited power; and (3) GC, a metric of directed interareal influence (see Method details). Coherence and power correlation are undirected metrics, whereas GC is a directed metric that allows the calculation of influences in both directions. For analyses at the level of site pairs, we used all possible site combinations—per monkey ≈23,000 coherence or power-correlation spectra, and ≈46,000 GC spectra (after exclusion of site pairs with spectra indicative of artifactual coupling, which amounted to 1.7% of all site pairs in monkey 1 and 1.1% in monkey 2; see Method details). The 15 simultaneously recorded cortical areas allowed for the analysis of FC for 105 area pairs. Each area

Figure 1. Stimuli, attention task, recording site distribution, FC spectra, and frequency bands

(A) Two macaque monkeys were trained to release a lever when a change occurred to the target stimulus, the stimulus with the same color as the fixation dot, while maintaining fixation and ignoring changes to the distractor stimulus, the stimulus with a different color than the fixation dot. Correct performance was rewarded with liquid reward (blue droplets). Task delays for Monkeys 1 and 2 are given in seconds. Errors bars indicate possible time of occurrence for cue onset and target change.

(B) Pooled recording sites of both monkeys on the surface of the INIA19 template brain (see Figure S1A for sites per monkey). Sites are colored according to the area color legend on the right, based on the Kennedy lab nomenclature (Markov et al., 2011). V1, primary visual cortex; V2, secondary visual cortex; 8L, lateral part of area 8/FEF; V4, fourth visual area; TEO, temporal-occipital area; DP, dorsal prelunate area; 7A and 7B, parts A and B of parietal area 7; TPt, temporo-parietal area (posterior auditory association cortex); 5, area 5; S1, primary somatosensory cortex; 8M, medial part of area 8/FEF; F1, corresponding to primary motor cortex; F2, corresponding to the caudal part of dorsal premotor cortex; F4, corresponding to the caudal part of the ventral premotor cortex. Spectra show examples of interareal coherence (in blue) and GC (green: feedforward; black: feedback, plain/dashed lines point to the cortical area sending feedforward/feedback projections). Spectra show means over all trials ± 99.9% confidence intervals from bootstrap estimates over trials.

(C) Each line is the average coherence spectrum for a pair of cortical areas, in 1 of the monkeys (Monkey 1: left plot; Monkey 2: right plot). With 15 simultaneously recorded cortical areas, there are 105 area pairs, hence, 105 average coherence spectra per plot. Each area has been recorded with several recording sites (see Method details). Therefore, each area pair corresponds to several interareal site pairs. The spectra of site pairs belonging to a given area pair were averaged for this plot. For each of the 4 frequency bands, the peak frequencies (PFs) and the corresponding full width at half-maximum (FWHM) are given in the Results and Method details, and the FWHMs are indicated in this figure by the gray-shaded areas.

See also Figure S1.

CellPress
OPEN ACCESS

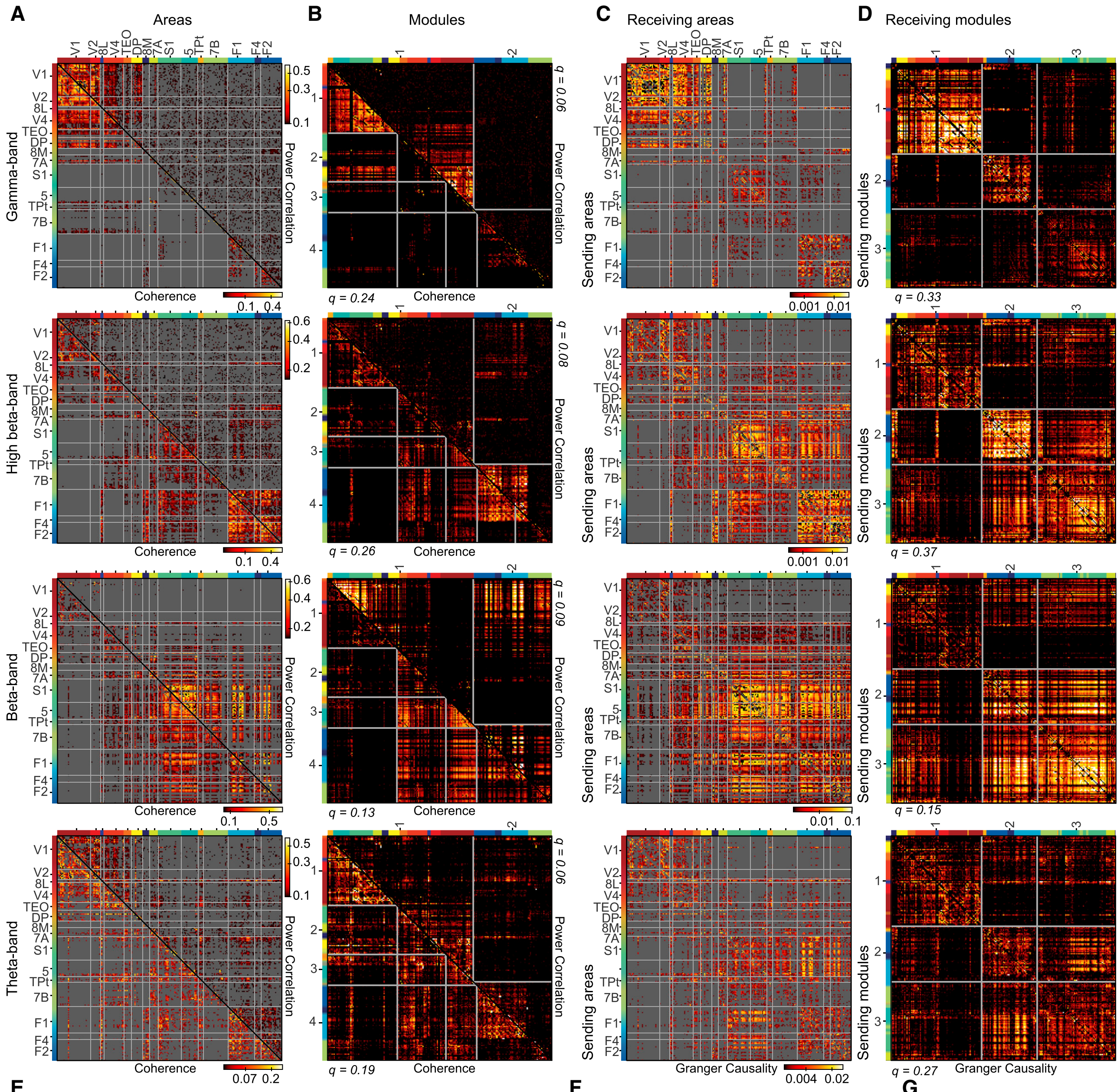

E

1 - R^2	γ, β*h*	γ, β	γ, θ	β*h*, β	β*h*, θ	β, θ
Coherence	0.73 (0.84)	0.91 (0.98)	0.72 (0.75)	0.45 (0.71)	0.58 (0.71)	0.67 (0.89)
Power Correlation	0.12 (0.98)	0.42 (1)	0.12 (0.88)	0.31 (0.87)	0.11 (0.90)	0.36 (0.96)
Granger Causality	0.55 (0.98)	0.75 (1)	0.75 (0.99)	0.29 (0.74)	0.55 (0.95)	0.49 (0.92)

F

γ	β*h*	β	θ
1 (1)	1 *(1)*	1 (1)	0.98 *(1)*
0.22 (0.95)	0.18 (0.95)	0.36 (0.87)	0.54 (0.96)
1 (1)	1 (1)	*1* (1)	0.99 *(1)*

G

γ	β*h*	β	θ
1 (1)	1 (1)	1 (1)	0.99 (1)

Figure 2. Brain rhythms define distinct interaction networks

(A–D) Data from monkey 1 (see Figure S2 for monkey 2). Note that the color scales are logarithmic.

(A) The 4 matrices in this column show coherence (lower triangular matrix) and power correlation (upper triangular matrix) for the frequency bands listed to their left. Each matrix entry corresponds to the respective FC value of 1 pair of recording sites, calculated across all available post-cue data epochs (see Method details), and averaged over the frequency bins in the respective frequency bands (see Results and Method details). Matrix entries with non-significant FC are masked in gray (non-parametric randomization test by shuffling data epochs, corrected for multiple comparisons across site pairs). The axes list the cortical areas, from which the sites have been recorded, with the areas ordered according to their hierarchical level (Chaudhuri et al., 2015). Area boundaries are indicated by gray lines on the matrices. Each area, and its corresponding recording sites, is given a color code. The sites will maintain these area-specific colors, when they are reordered in the modularity analysis shown in (B) and (D).

(B) Same FC values as in (A), but reordered according to modules obtained from a consensus modularity analysis (see Method details). Modules are separated by gray lines. The modularity analysis was performed separately for coherence and power correlation (i.e., separately on those triangular matrices), and consensus

(legend continued on next page)

was recorded from several sites (see Method details), such that each interareal interaction was assessed by several interareal site pairs. The spectra of site pairs belonging to a given area pair were averaged for all of the analyses at the level of area pairs.

Figure 1B shows example coherence and GC spectra for several pairs of cortical areas. These spectra show distinct peaks, which are specific for the respective pair of brain areas. Across all 105 area pairs, average FC spectra showed peaks for 4 characteristic brain rhythms, with some individual differences across the 2 monkeys (Figure 1C for coherence, Figure S1B for power correlation and GC): The theta rhythm (3 ± 2 Hz in monkey 1 and 4 ± 3 Hz in monkey 2; peak ± full width at half-maximum [FWHM]), the beta rhythm (18 ± 5 Hz in monkey 1 and 15 ± 5 Hz in monkey 2), the high-beta rhythm (34 ± 5 Hz in monkey 1 and 32 ± 4 Hz in monkey 2), and the gamma rhythm (75 ± 8 Hz in monkey 1 and 62 ± 8 Hz in monkey 2) (Figures 1B, 1C, and S1B). All further analyses focus on these 4 rhythms. For analyses at the 4 corresponding frequency bands, FC values were averaged over the frequency bins in the monkey-specific frequency bands and subsequently averaged over monkeys. For analyses of full spectra, the FC spectra were aligned to the 4 monkey-specific peak frequencies (PFs) and subsequently averaged over monkeys.

Different rhythms define distinct FC networks

For each band, we calculated all FC metrics for all pairs of recording sites. The resulting FC matrices for monkey 1 are shown in Figures 2A and 2C; The same analysis for monkey 2 is shown in Figures S2A and S2C; The individual matrices cannot be directly averaged over animals because the numbers of recording sites per area differ between monkeys (see Method details). The 4 frequency bands showed distinct interaction networks. We defined distinctiveness D = 1-R^2, with R^2 being the coefficient of determination (squared Pearson correlation coefficient) across all site pairs, between matrices of one FC type (concatenated over the 2 animals), separately for all combinations of frequency bands (Figure 2E). As an example, GC networks were least distinct between beta and high-beta, with D = 0.29, and most distinct between beta and gamma, with D = 0.75. As it is known that FC metrics in different frequency bands tend to jointly decrease with distance (Leopold et al., 2003; Nelson and Pouget, 2012), we partialized the calculation of R^2 for distance, which increased all D values. Hence for GC networks, D between beta and high-beta increased to 0.74, and D between beta and gamma increased to 1. Note that D was overall much lower for power correlation than for coherence or GC.

The almost complete distinctiveness, after distance partialization, between FC networks for some frequency-band combinations is remarkable, given that all networks emerge on the same AC network. If the shared AC network does not account for the distinct interaction networks, then these interaction networks may be accounted for by different distributions of power across recording sites for the different frequency bands. We calculated the same distinctiveness metric between FC and the co-occurrence of power, concretely, for example, between the gamma-GC matrix and the matrix of the products of average gamma power values of the corresponding site pairs (specifically $log_{10}(\sqrt{power_{site1} \times power_{site2}})$), again either with or without partializing for cortical distance (Figure 2F). Staying with the example of the gamma GC network, distinctiveness was 1, both with and without partialization for distance. We considered that GC may be particularly related to power in the sending-area sites, and therefore also calculated D between, for example, the gamma-GC matrix and the matrix of average gamma power values of the corresponding sending-area sites (specifically $\log_{10}$(power); Figure 2G). This left D values essentially unchanged at 1 (with and without partialization). Thus, band-specific FC networks, as assessed, for example, by gamma and beta GC, are highly distinct and contain structure beyond power distributions. Note that the D values were overall much lower for power correlation than for coherence or GC.

Note that the GC matrices (Figure 2C) list both the sending areas (on the y axis) and the receiving areas (on the x axis) according to their hierarchical level. In this manner, differences between GC in the bottom-up versus top-down direction described previously (Bastos et al., 2015b) can be appreciated by appropriately comparing the upper and lower triangular GC matrices (for the 8 visual areas investigated in Bastos et al. [2015b], i.e., V1, V2, 8L, V4, TEO, DP, 8M, 7A). This illustrates that these bottom-up versus top-down differences account for only a small fraction of the full GC variability that we investigate here.

Visual inspection of the FC matrices suggested that band-specific interaction networks may form distinct modules. We

was obtained over the 4 frequency bands. The color codes on the margin indicate per site the respective cortical area as introduced in (A); note that those color codes are separate for the upper and lower triangular matrix.
(C) Similar to (A), but for GC. GC is a directed metric, requiring the full matrix. Each matrix entry corresponds to the GC from a site in the cortical area listed on the y axis to a site in the area listed on the x axis.
(D) Similar to (B), but for GC. GC modularity analysis was performed on the full matrix, and consensus community structure was obtained over the 4 frequency bands.
(E–G) Data averaged over both monkeys.
(E) Distinctiveness (1-R^2; see Results) between patterns of FC of a given type (as listed per row), for all combinations of frequency bands (listed per column). The patterns of FC are the triangular matrices shown in (A) for coherence and power correlation, and the full matrices shown in (C) for GC. Values in parentheses are the distinctiveness after partialization for distance on the cortical surface.
(F) Distinctiveness (1-R^2; see Results) between patterns of FC of a given type (as listed per row in E), and the pattern of the product of power at the respective sites (specifically $log_{10}(\sqrt{power_{site1} \times power_{site2}})$), and in the frequency bands listed per column. Values in parentheses are the distinctiveness after partialization for distance on the cortical surface.
(G) Same as (F), but only for GC and replacing the product of power by the power at the sending site, the site from which the GC originates.
See also Figure S2.

CellPress
OPEN ACCESS

therefore performed a modularity analysis that rearranges connectivity matrices so that highly connected sites ("nodes") are contained in the same module (see Method details; Rubinov and Sporns, 2010). The resulting partitioning is referred to as community structure. To obtain 1 consensus community structure over the 4 frequency bands, we combined (for example) the GC matrices for the 4 bands in the same way as previous studies combined connectivity matrices over participants. For the example of GC in monkey 1 (Figure 2D), this revealed that module 1 was dominated by gamma, module 2 by high-beta, and module 3 by beta, with relatively strong links between modules 2 and 3, and weak links between both of these modules and module 1. The modularity analysis for monkey 2 is shown in Figure S2 and shows an overall similar pattern for GC. To characterize overall modularity, we calculated the modularity index. Networks with high modularity index show strong intramodule and weak intermodule connectivity. Modularity indices are reported in Figures 2 and S2, on the margins of the corresponding (triangular) matrices. Modularity indices were much lower for power correlation than for coherence or GC.

To investigate whether band-specific FC networks have meaningful brain-topographical patterns, we calculated, for each site pair, a metric that is referred to as "strength." Coherence strength of a recording site is the average coherence of that site with all other sites (excluding sites within a 2-mm radius to avoid residual volume conduction effects). Power correlation strength is defined accordingly. For GC, we defined the GC-outflow strength of a site as the average GC of that site to all other sites, and GC-inflow strength as the average GC to that site from all other sites. Averaging collapses the FC matrices onto their margins, and allows visualization of topographical distributions as strength maps (Figure 3, averaged over monkeys; see Method details). Strength maps, for most combinations of FC type and frequency band, showed contiguous clusters with the tendency to respect sulcal anatomy, and thereby most strength maps showed clear and meaningful topographies.

We also demonstrate that weak long-distance FC deviates significantly from randomized intersite FC (Figure S3A; see Method details). Significant interareal FC covers long distances (>20 mm) for all frequencies; FC at gamma extends up to 50 mm and FC at beta, theta, and high-beta beyond 60 mm.

FC network topographies correlate with AC

We next investigated whether these FC patterns could be partly explained by known patterns of AC. We previously showed that GC asymmetries are related to the feedforward/feedback character of the respective anatomical projections (Bastos et al., 2015b), as quantified by the supragranular labeled neuron (SLN) percentage value (Barone et al., 2000; Markov et al., 2014b). In the present study, we address another fundamental aspect of an anatomical projection, namely its strength, which is captured by the fraction of labeled neurons (FLN) (Vezoli et al., 2004). After injection of a retrograde tracer into area A, retrogradely labeled neurons are counted across the brain (e.g., in area B). The FLN of the projection from B to A is the number of labeled neurons found in B divided by the total number of neurons found across the brain. In this way, FLN reflects the fraction of neurons projecting to A that originates in B. While the majority of projections to a given cortical area arises from within the area itself (~80%), we are concerned here with projections arising from other areas and so estimate the extrinsic fraction of labeled neurons (FLNe) (Markov et al., 2011).

We were interested in how interareal FC, assessed by coherence, power correlation, and GC, relates to interareal AC, assessed by FLNe. We used a dataset based on 28 retrograde tracer injections across 14 cortical areas (Figure 4A). These 14 areas were identical to the 15 areas recorded electrophysiologically, except that they did not include the temporo-parietal area (TPt). The 14 areas resulted in a 14 × 14 matrix of 182 FLNe values (Figure 4C). Note that this is a directed matrix of AC, in which FLNe from area A to area B is quantified independently of the FLNe in the reverse direction. Thus, the following analyses relate the full FLNe matrix (Figure 4C) to the corresponding part of the full GC matrix. By contrast, the matrices of coherence and power correlation assess overall FC irrespective of direction. To relate them to AC strength, we averaged FLNe over the 2 directions, giving a triangular matrix with 91 entries (Figure 4B). Spectra were averaged over all site pairs of a given area pair (Figures S3C and S3D) and subsequently over the 2 animals (Figure S4). Hence, we determined the PFs per monkey and per rhythm (theta, beta, high-beta, gamma), and expressed frequencies relative to the per-monkey PFs. This suggested that overall, coherence and GC increased with increasing FLNe (Figure S4A). For this analysis, we excluded FLNe values based on <10 labeled neurons (Figure S4A) to ensure the reliability of FLNe estimation (Markov et al., 2014a). The pattern held, when we included those FLNe values (Figure S4B), or when we replaced them by estimates from a model fitted to neuron counts from the non-zero FLNe values (Figure S4C; see Method details).

FLNe-FC correlations differ across FC types and frequencies

To quantify the observed patterns, we performed linear regression analysis between $\log_{10}$(FC) and $\log_{10}$(FLNe) separately for all combinations of FC type (coherence, power correlation and GC) and frequency band (theta, beta, high-beta, gamma) (Figure 5). For each combination, there was a significantly positive correlation ($p < 4.17E-3$ after Bonferroni correction for multiple comparisons), but with a wide range of correlation strengths (Figure 5A). FLNe was least predictive for FC at beta, with explained variance (R^2 values) for beta power correlation or beta GC of 0.14. FLNe was most predictive of theta ($R^2 = 0.47$) and high-beta coherence ($R^2 = 0.39$) and gamma GC ($R^2 = 0.42$).

To capture the size of the FLNe effect on FC, we used a regression analysis (Figure 5B). We performed a simple linear regression with the dependent variable $\log_{10}$(FC) and the independent variable $\log_{10}$(FLNe). We then used the linear fit to calculate the expected FC at the minimal FLNe value, in other words, FC(min(FLNe)), and at the maximal FLNe value, in other words, FC(max(FLNe)). The ratio FC(max(FLNe)) / FC(min(FLNe)) was used as the FLNe-related FC change (Figures 5B and 5C). This metric is related to the regression slope, but normalizes for differences in FC across frequencies that are not due to FLNe. We derived error estimates by 100 bootstrap replications over trials (Figures 5B and 5C) (Efron and Tibshirani, 1994).

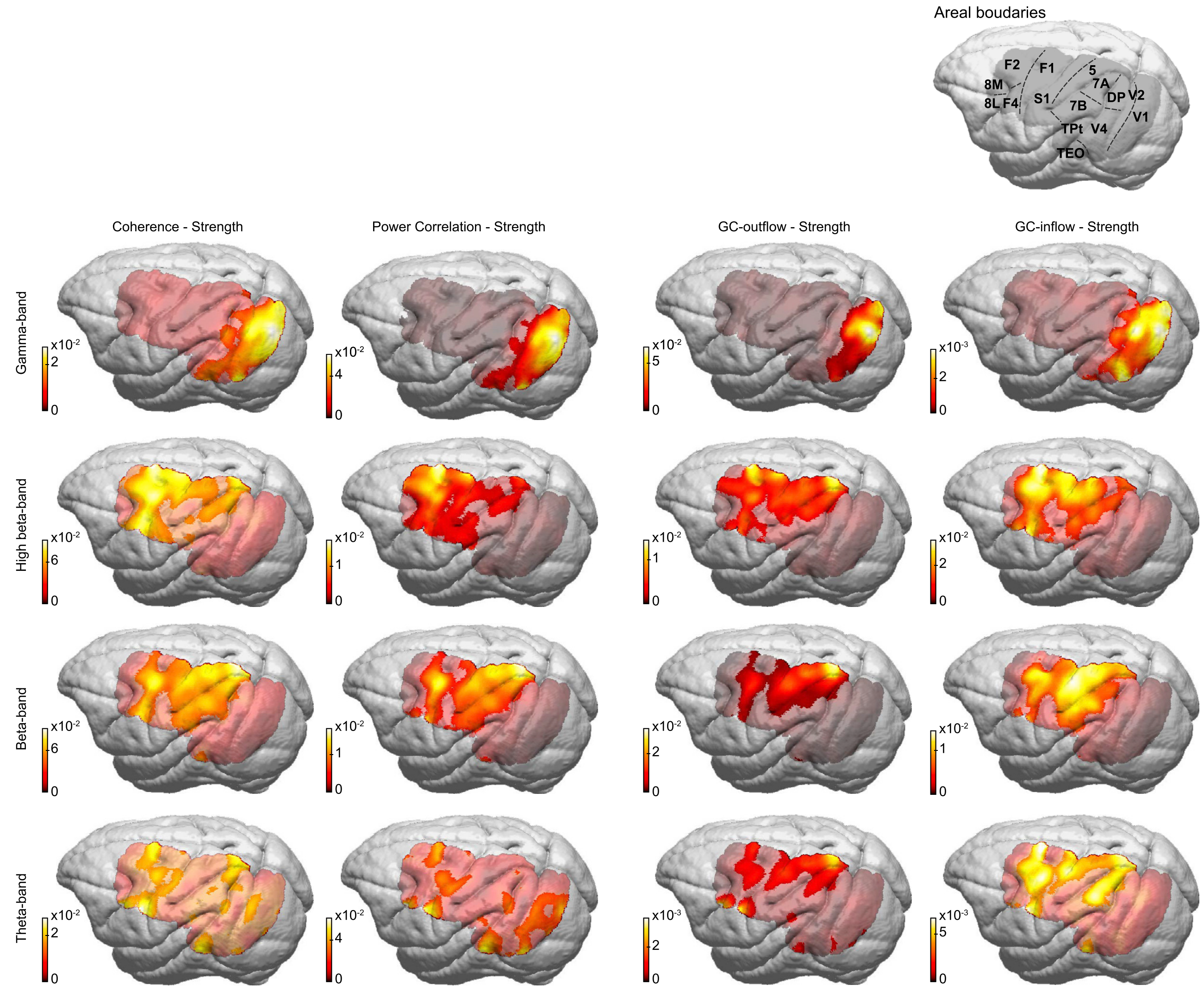

Figure 3. Topographies of FC strengths

All of the panels show FC strength topographies averaged over both monkeys, with the FC type (coherence, power correlation, GC outflow, GC inflow) listed above the columns and the frequency bands listed to the left of the rows. The strength metric for a given FC type and frequency band is defined per recording site: the coherence (or power correlation) strength of a given site is the average coherence (or power correlation) of that site with all other sites; the GC outflow strength of a given site is the average GC directed from that site to all other sites; the GC inflow strength of a given site is the average GC directed to that site from all other sites. Strength topographies of the 2 monkeys have been co-registered to the same template brain and then averaged over the monkeys. Gray masking indicates non-significant strength (comparison to a random graph with equal weight distribution; false discovery [FDR] corrected for multiple comparisons over sites; see Method details). The template brain in the upper right of the figure shows the cortical area boundaries.
See also Figure S3.

This analysis revealed that the 3 types of FC showed different degrees of dependency with FLNe. The power correlation was the least FLNe dependent, coherence was intermediate, and GC was by far the most strongly FLNe dependent. The spectrum for power correlation did not show any clear peaks (even when scaled independently). The spectrum for coherence showed peaks for high-beta and gamma, and a local trough for beta, while that for GC showed a small peak in the theta range and substantial peaks for high-beta and gamma, and again a local trough for beta. These results suggest that the dependence of coherence and GC on AC has a characteristic spectral pattern. At the individual PFs for gamma and high-beta (and partly also for theta), this dependence is stronger than at neighboring frequencies; by contrast, at the individual PFs for beta, this dependence is weaker than at neighboring frequencies.

FC is both stimulation and task dependent, which is likely to dynamically influence its dependence on AC. Therefore, we obtained this spectrum for GC separately for the pre-stimulus baseline period, and for the 2 attention conditions during the post-cue period (Figure S5A). During the baseline, the spectrum showed much less of a gamma peak and higher values for beta. With

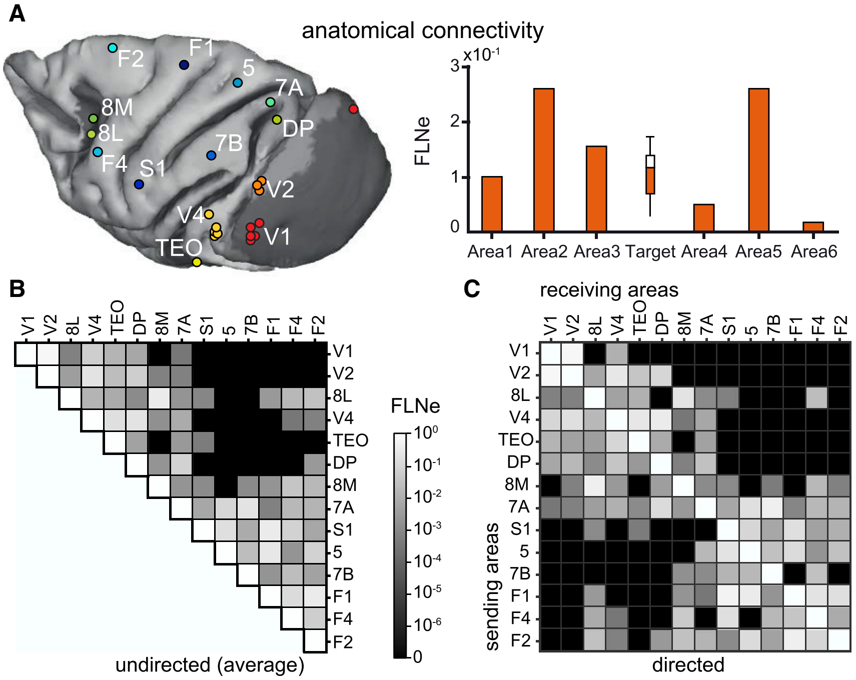

Figure 4. Anatomical connectivity assessed by FLNe

(A) Each colored dot indicates the injection site of a retrograde tracer, shown here on a template brain.

(B) FLNe values for all indicated pairs of areas, averaged over the projections in the respective 2 directions, for example, V1-to-V4 and V4-to-V1.

(C) FLNe values for all indicated projections from the areas listed on the y axis to the areas listed on the x axis. Black matrix entries indicate projections for which <10 labeled neurons were counted (see also Figure S4). Those entries were discarded for the average shown in (B).

(B and C) Note the logarithmic grayscale, which applies to both panels and spans 6 orders of magnitude.

See also Figure S4.

attention, values tended to be reduced for theta and high-beta and enhanced for gamma.

FC-FLNe correlations are not explained by distance, yet FC predicts FLNe

FLNe declines exponentially with interareal distance, a phenomenon referred to as the exponential-distance rule (EDR), characterized by the exponential decay rate, λ (see Ercsey-Ravasz et al., 2013). The EDR held for the subset of areas investigated here (Figure S5B), with $\lambda = 0.202$/mm for distance through white matter, consistent with previous reports (Ercsey-Ravasz et al., 2013). Importantly, the EDR holds for the present FC data (Figure S5B, all bands averaged for simplicity), but with exponential decay rates that were substantially lower (0.01–0.08/mm; values per band and FC type reported in Table S1) (Fischer et al., 2018; Leopold et al., 2003; Nelson and Pouget, 2012). Figure S5B shows linear relationships between $\log_{10}$(FLNe) or $\log_{10}$(FC) and distance, which is equivalent to an exponential decay of FLNe or FC with distance. Furthermore, Figure 5A shows linear relationships between $\log_{10}$(FC) and $\log_{10}$(FLNe). Hence, for further regression analyses, we use $\log_{10}$(FC), $\log_{10}$(FLNe), and the non-log-transformed distance.

The joint dependence of FC and FLNe on distance may explain the observed correlation between FLNe and FC. Note that this would not explain the observed frequency dependence of the FLNe-FC correlation. Nevertheless, we investigated the extent to which the FLNe-FC relation is explained by distance by performing a multiple linear regression (MR), with the dependent variable being $\log_{10}$(FLNe) and the independent variables being $\log_{10}$(FC) for theta, beta, high-beta, and gamma, and additionally the distance (as distance metric, we use distance on the cortical surface [Figure 6] or distance through the white matter [Figure S6], both giving similar results [Table S2]). Note that this analysis also informs us whether FLNe can be partly predicted by FC metrics. Figure S6E shows that FC alone (without distance information) is strongly predictive of FLNe, with explained variance (R^2 full model) ranging from 0.48 for GC to 0.56 for coherence (Table S2). This is interesting because FLNe cannot be obtained for the human brain, as it requires active retrograde transport of tracer injected into the living brain (Donahue et al., 2016). By contrast, FC, and in particular GC, can be obtained for the human brain, and GC has already been shown to relate to the anatomical SLN metric (Michalareas et al., 2016).

The MR analysis revealed that all of the FC metrics were significantly predictive of FLNe for some frequency bands, and importantly, that this was the case when distance was included as an independent variable (Figure 6A). Specifically, power correlation was significantly FLNe predictive in the beta and gamma bands. Coherence and GC were FLNe predictive in the gamma band. Note that those FC metrics predicted FLNe so accurately that the contribution of distance was not significant. Figure 6A shows results obtained for distance measured on the cortical surface. When distance was measured through white matter, this explained slightly more FLNe variance, but overall, the pattern of results was highly similar (Figure S6).

To further investigate the differential FLNe-predictive power of the FC metrics in the different bands and of distance, we performed the following analysis. We determined R^2 values for the full MR models, separately for power correlation, coherence, and GC (Figure 6B). We repeated this analysis after excluding either one of the frequency bands or distance as an independent variable, in other words, we calculated R^2 values for reduced models. Figure 6C shows the R^2 difference between the full and the reduced model (similar to a stepwise linear regression approach); the x axis lists the independent variable that had been removed, such that the corresponding y axis values reflect the improvement in R^2 value when this variable is included. For all FC metrics, the removal of distance reduced R^2 values by only relatively small amounts, less than the removal of most of the individual band-wise FC metrics. As above, distance through white matter had a larger effect,

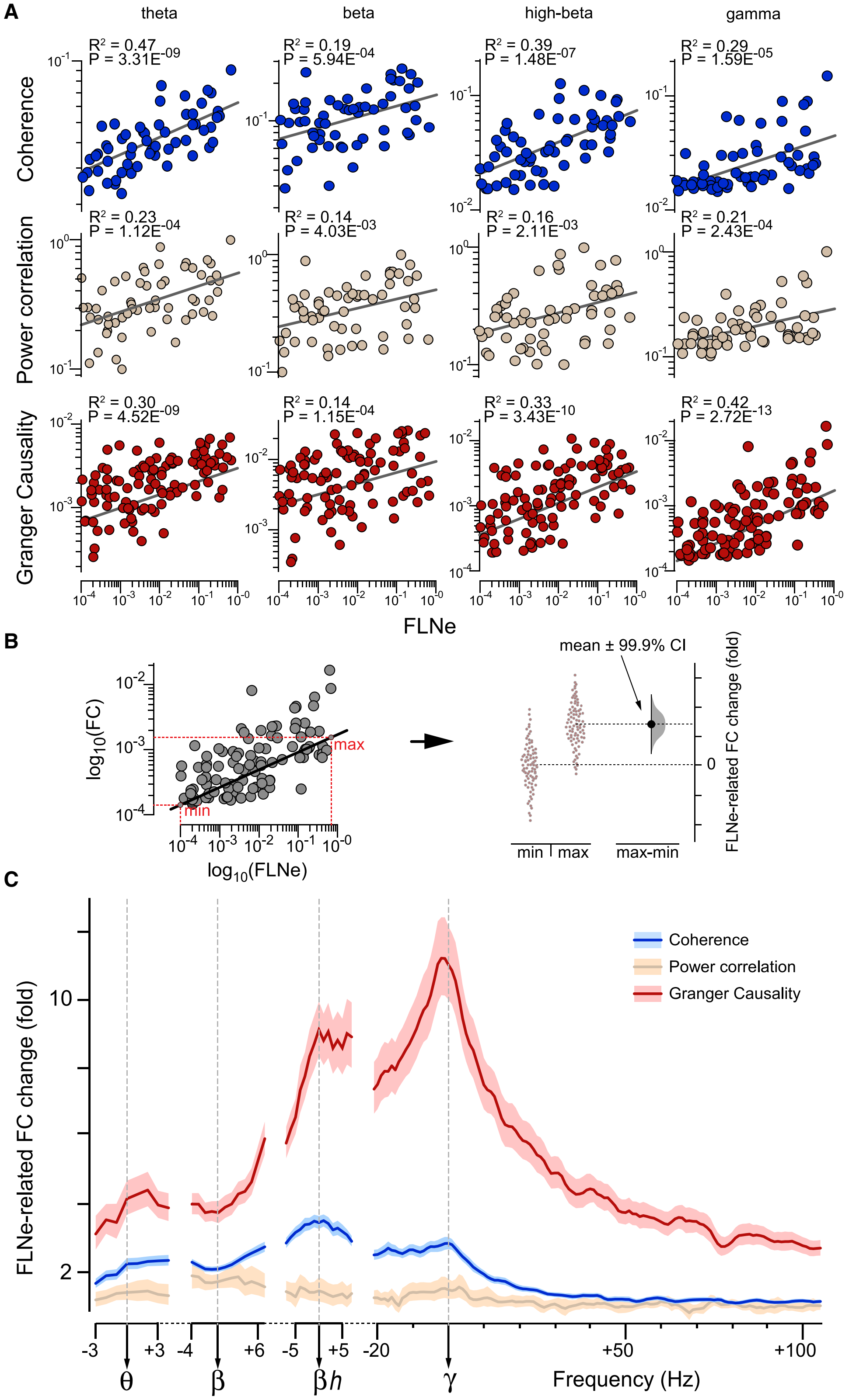

(legend on next page)

CellPress
OPEN ACCESS

but overall, the pattern of results was highly similar (Figure S6C). Also, complete removal of distance as an independent variable left the overall pattern of results qualitatively unchanged, and as expected, regression coefficients for FC increased (Figures S6D–S6F).

Note that these analyses revealed that all $\log_{10}$(FC) metrics were linearly related to distance (Figure S5B), leading to a multi-collinearity among the independent variables. We performed several analyses to control for this (Figures S5C, S5D, and S7C–S7E; Table S2; see Method details). We also used simpler MR models, each with the dependent variable being $\log_{10}$(FLNe), and each with the independent variables being distance and the $\log_{10}$(FC) of merely 1 frequency band (Figure S8). Most of these models found a significant effect of distance. For the power correlation, only gamma was significantly FLNe predictive. For coherence and GC, all frequency bands except beta were significantly FLNe predictive.

FLNe-FC relations depend on corresponding SLN values

The analyses so far suggest that FLNe partly determines FC values, with a specific spectral pattern. We had previously found that one aspect of FC, namely GC between 2 areas, is related to another aspect of AC, namely the feedforward/feedback characteristics of the corresponding connections captured by the SLN metric. When retrograde tracer is injected in area A and the labeled cells are counted in area B, separately for the supragranular (Nsupra) and infragranular (Ninfra) compartments of B, then the SLN of the A-to-B projection is

$$\text{Nsupra} \ / \ (\text{Nsupra} + \text{Ninfra}).$$

The larger the SLN metric, the more the corresponding projection is of the feedforward type. Projections with SLN > 0.5 are considered feedforward, and projections with SLN < 0.5 are considered feedback. We previously found that if the SLN indicates that area B is higher in the hierarchy than area A, then theta- and gamma-band GC is stronger in the A-to-B (feedforward) than the B-to-A (feedback) direction, whereas beta-band GC is stronger in the B-to-A (feedback) than the A-to-B (feedforward) direction (Bastos et al., 2015b). Here, we investigate whether this SLN-GC relationship influences the above-described dependence of GC on FLNe.

To investigate this, we selected 2 groups of projections, namely strongly feedforward projections, with SLN > 0.7, and strongly feedback projections, with SLN < 0.3. Within those 2 groups, we calculated the FLNe-related GC change for all frequencies (i.e., as in Figure 5C, but split for SLN). For feedforward projections, the change spectrum showed peaks at theta and gamma, separated by a relative trough around beta (Figure 7A, green line). For feedback projections, the change spectrum showed the strongest peak at high-beta and a smaller one at gamma (Figure 7A, black line). Figure 7B shows the corresponding scatterplots at the PFs of each rhythm. For gamma GC, FLNe explained 48% (R^2 value) of the variance in the feedforward and 37% in the feedback direction, whereas for beta GC, FLNe explained 15% in the feedback direction and none in the feedforward direction (0.0001%, not significant [n.s.]). The absence of a significant relation between FLNe and beta GC in the feedforward direction is also reflected in the beta-band trough (Figure 7A, in green). We next determined the asymmetry index of the FLNe-related changes by taking the difference of the feedforward- minus the feedback-related spectrum and dividing by their sum (Figure 7A, inset). This asymmetry index showed particularly pronounced negative values for beta and positive values for gamma, with much smaller effects for theta and high-beta. To test whether this result depended on the particular SLN cutoff (0.7/0.3), we repeated the same analysis for various cutoffs and found that the observed effects generally showed a gradual dependence on SLN values (Figure 7C).

When we perform this analysis separately for the 2 attention conditions in the post-cue period, we find that attention strengthens particularly the relation between FLNe of feedforward connections and feedforward GC in the gamma band (Figure S7A). By contrast, this relation is essentially lost during the baseline, when gamma is weak (Figure S7B).

Mapping frequency-specific FC networks onto the anatomical core-periphery structure

We established that FC is related to both the strength (FLNe) and the feedforward character (SLN) of anatomical projections. The analysis of anatomical projections has integrated those 2 metrics, demonstrating that areas can be arranged in a bowtie structure: some areas are in the knot (the core) and others in the two fans (peripheries) of the bowtie (Markov et al., 2013). Areas inside the core are densely interconnected and with strong (high FLNe) connections, whereas areas in the fans are connected less densely with areas in the core and with weaker connections to those areas. We found FC strength in the gamma frequency band to dominate in the left fan areas of the bowtie structure (Figure 8A), the areas sending predominantly feedforward projections to the core. FC strength at other frequencies was more evenly distributed among core and periphery (Figures 8B–8D). Overall, FC strength was the strongest in the high-beta frequency band for the core and in the beta

Figure 5. FC and AC display frequency-dependent covariance

(A) Scatterplots between the 3 FC types (indicated to the left of the rows) and FLNe. For coherence and power correlation, each dot corresponds to a pair of areas, for which the combined FLNe in both directions was based on >10 labeled neurons (N = 60). For GC, each dot corresponds to an anatomical projection for which the FLNe in the same direction as the corresponding GC was based on >10 labeled neurons (100). FC values were averaged over monkeys before correlation analysis. Note logarithmic scaling on x and y axes.

(B) With both axes in $\log_{10}$ units, subtraction of FC values between minimum and maximum AC values (left) can be interpreted as FLNe-related fold change of FC (right).

(C) FLNe-related FC change as a function of FC frequency. Log_{10}(FC) spectra (color coded, legend top right) have been aligned to individual peak frequencies before averaging over monkeys and then correlated with $\log_{10}$(FLNe). Means over all trials ± 99.9% confidence intervals from bootstrap estimates over trials. See also Figure S5.

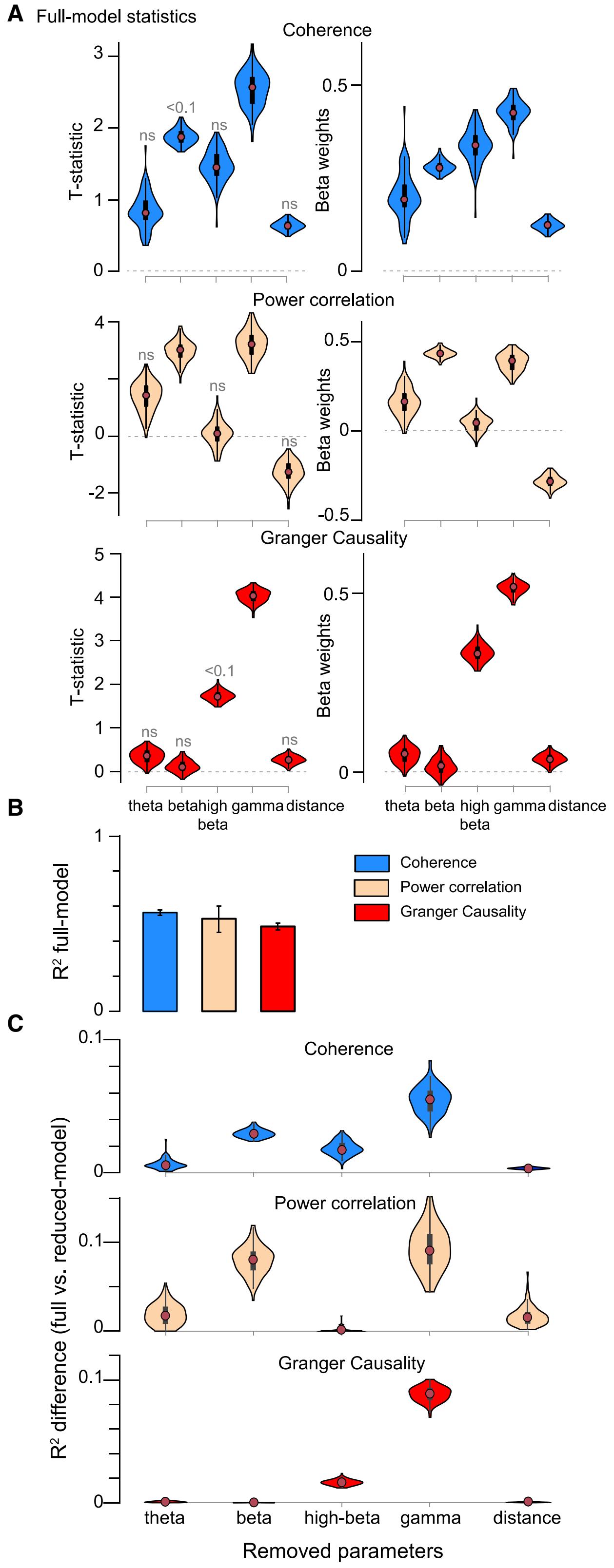

Figure 6. Multiple regression discloses distance as poor predictor of the structure-function relationship

(A) Violin plots of model estimates (left column: t-statistic; right column: beta coefficients) for each of the 5 variables considered, namely FC in the 4 frequency bands and distance, separately per FC type (as indicated above each row).

(B) Total explained variance (R^2) for the 3 models (color-legend, top right). Means ± 99.9% confidence intervals from bootstrap estimates over trials.

(C) Difference in total explained variance of the 3 models (same color code as in B) between the full and the reduced model, after removing the parameter listed on the x axis. This estimates the contribution of each of the 5 parameters to the total explained variance.

See also Figures S6 and S8.

frequency band for right-fan areas of the bowtie structure (Figure 8C), areas sending predominantly feedback projections to the core.

DISCUSSION

In summary, we report and make available, for each of 4 rhythms, the full pattern of ≈23,000 coherence and power-correlation values and ≈46,000 GC values, among 218 recording sites distributed over 105 pairs of cortical areas in 2 awake, task-performing macaque monkeys. We find that the rhythms define distinct interaction networks that are largely independent of the spatial distribution of power, particularly for coherence and GC. Modularity analyses revealed that beta, high-beta, and gamma GC are largely contained in separate modules, with relatively strong links between the beta and high-beta modules, and relatively weak links between beta and gamma modules. The coexistence of distinct rhythm-specific functional interaction networks on a fixed anatomical backbone partially reflects the differential dependence of the rhythmic interactions on cortico-cortical anatomical projections. Projection strength, assessed by FLNe, was predictive of all FC types in all frequency bands, but with marked differences: weakest for power correlation, intermediate for coherence, and strongest for GC, and weakest for beta and much stronger for high-beta and gamma. This suggests that high-beta and particularly gamma-based interactions prominently depend on direct cortico-cortical projections. The relative independence of beta FC from AC may be due to the known geometry of feedback projections (Markov et al., 2014b) and/or to a more prominent dependence on pathways involving subcortical structures (Guillery and Sherman, 2002); it may make beta an ideal candidate to quickly establish new FC structures based on learning and top-down cognition, including prediction (Miller et al., 2018). Intriguingly, FC in the different frequency bands jointly predicted about half of the FLNe variability across projections. In a MR, this rendered the previously reported strong influence of distance insignificant. As FC and AC values for this study have been obtained in separate animals, the prediction of AC by FC in a given individual is likely even higher. This suggests that FC metrics could provide estimates for AC in humans, with relevance for science and medicine (Becker and Hervais-Adelman, 2020; Smith et al., 2015). Finally, GC in the gamma band showed a much stronger relation to FLNe in the feedforward than in the feedback direction, and conversely, GC in the beta band showed no significant relation

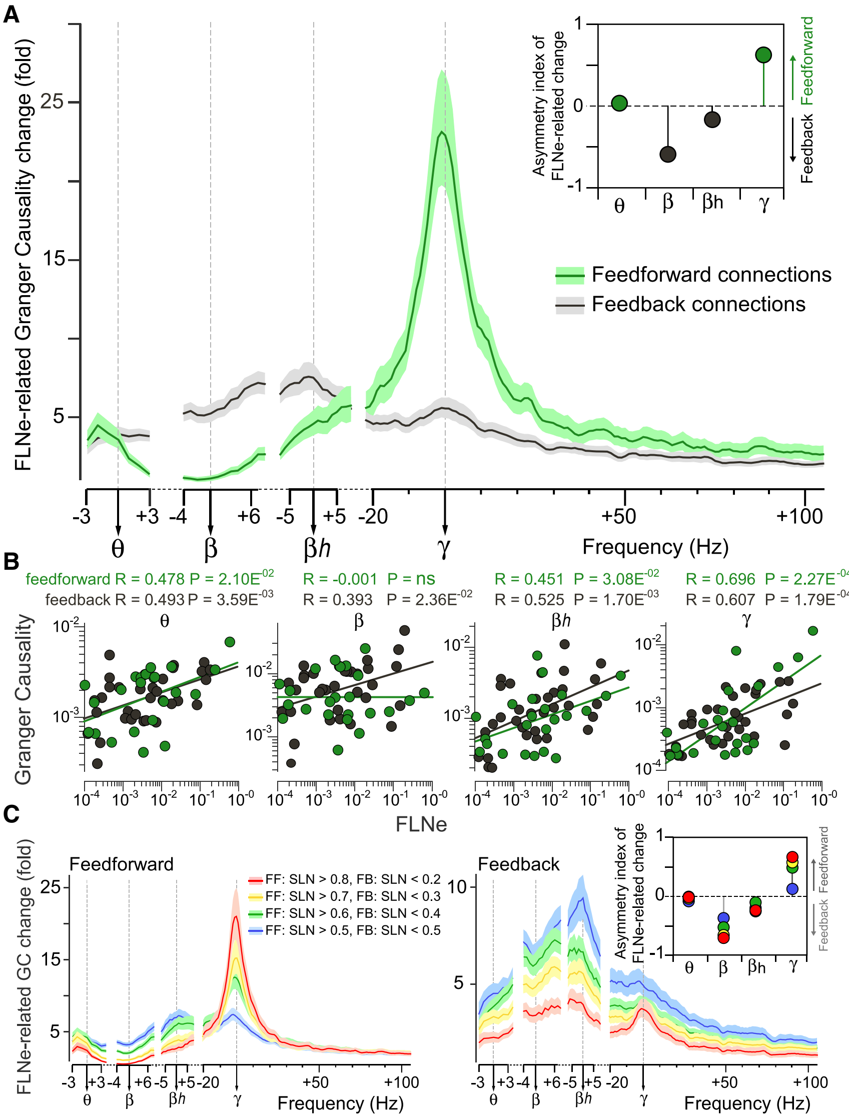

Figure 7. Anatomical influence on FC strength depends on frequency and direction

(A) Frequency-resolved spectra of the FLNe-related change in GC (same as red line in Figure 5C), plotted separately for feedforward (in green, SLN $\geq$ 0.7) and feedback connections (in black, SLN $\leq$ 0.3), extracted from linear regression shown in (B). Inset (top right) displays asymmetry index (see Results) for all frequency bands. A positive (negative) asymmetry index indicates larger effect size in feedforward (feedback) direction.

(B) Same as bottom row of Figure 5A, but separately for feedforward (green) and feedback (black) connection, as defined for (A).

(C) Same as (A), but varying the selection threshold for feedforward (left) or feedback (right) connections. Left plot: selecting more strongly feedforward projections, with higher SLN thresholds, resulted in lower FLNe-related GC changes for all frequency bands, except the gamma band, where this effect reversed. Right plot: selecting more strongly feedback projections, with lower SLN thresholds, resulted in lower FLNe-related GC changes. Note different axes scales for feedforward and feedback. Inset (top right): asymmetry index (see Results) for varying threshold. Color code for varying SLN thresholds detailed in legend. Ordinate axes in (A) and (C) start from 1. All plots show means over all trials ± 99.9% confidence intervals from bootstrap estimates over trials.

See also Figure S7.

to feedforward, but did show a sizeable relation to feedback FLNe.

This latter set of results is likely related to our previous finding that for a given pair of areas in visual cortex, beta GC is stronger in the feedback than the feedforward direction, whereas theta and gamma GC are stronger in the feedforward than the feedback direction (Bastos et al., 2015b). Across area pairs, interareal GC asymmetries were linearly related to the corresponding interareal hierarchical separation, as quantified by the anatomical SLN metric. SLN quantifies, for a given anatomical projection, the extent to which it originates from supragranular neurons. The more a projection is feedforward (feedback) (i.e., the more hierarchical levels it bridges in the feedforward [feedback] direction), the closer its SLN is to 1 (to 0). SLN is normalized for the total number of parent neurons of the projection and is independent of projection strength. By contrast, FLNe quantifies, for a given anatomical projection, how many neurons it comprises, normalized by the total number of labeled neurons (see below for more discussion on this). FLNe does not take the laminar distribution of the parent neurons into account, and it is thereby independent of the feedforward/feedback character of the projection. In fact, FLNe and SLN have an inverted U-shaped relation. The strongest projections, with the largest FLNe, are between areas on similar hierarchical levels, with SLN close to 0.5 (Markov et al., 2013). Across interareal projections, FLNe ranges over 5 orders of magnitude. Here, we have related this large range of FLNe values to corresponding values in coherence, power correlation, and GC across an edge-complete 14 × 14 matrix, including both visual and non-visual areas.

The calculation of FLNe involves a normalization. When area B is the area injected with retrograde tracer, the FLNe from area A to area B is the number of labeled neurons in A normalized by the total number of retrogradely labeled neurons outside B. Therefore, FLNe can be considered to be a strength metric of the A-to-B projection relative to all other projections to B. Given this normalization of FLNe, one could consider similarly normalizing GC from A to B by the total GC inflow to B from all recorded areas. We performed this GC normalization and repeated the MR analysis of Figures 6A and 6C. Overall, this increased the effects

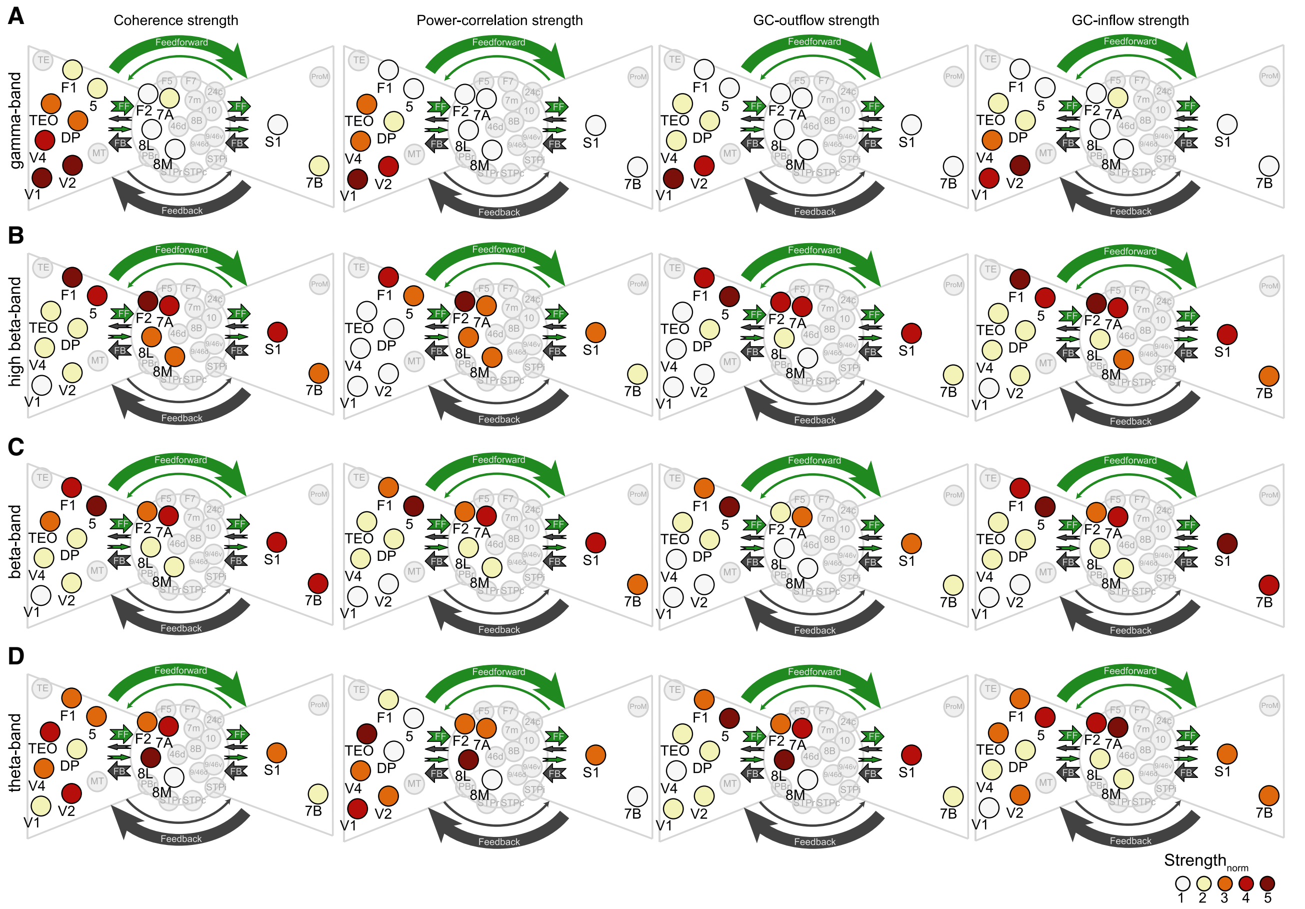

Figure 8. FC strength displayed on the AC-derived core-periphery structure

For cortical areas both recorded by the ECoG and used to build the core-periphery structure (highlighted areas) (Markov et al., 2013), the color code displays the FC strength of the respective area, separately per FC type (as indicated above the columns), and separately per frequency band (as indicated to the left of the rows). FC strength values were averaged over monkeys before normalization into the range 1–5 (color scale, bottom right), separately for each frequency band. See also Figure S3.

for beta while reducing the effects for gamma, although effects still remained much weaker for beta than gamma, and the latter at similar levels as high-beta (and no frequency band reaching significance). Importantly, distance still had relatively minor effects. Note that this GC normalization is dependent on the specific areas recorded by our ECoG, which constitutes only a subset of areas and therefore cannot strictly be compared to the normalization of FLNe. Note also that GC from A to B is normalized by the power in B. The power in B can be considered a metric of the total, interareal and local, synaptic input to B (Pesaran et al., 2018). Thus, GC already entails a normalization similar to that of FLNe.

The relation of FLNe with FC metrics was weakest for power correlation, intermediate for coherence, and strongest for GC. The fact that GC is strongly related to FLNe may partly reflect the fact that GC assesses the strength of the directed interareal influence, just as FLNe assesses the strength of the directed interareal projection. Anatomical projections are always directed from the area containing the parent neurons to the area containing the synaptic contacts. Thus, there is a natural correspondence between FLNe and GC. Intriguingly, GC is also particularly interesting for using the observed prediction of AC by FC in humans. FC metrics based on non-invasively recorded signals from the human brain are challenging to interpret because those signals reflect mixtures of many brain sources (Palva et al., 2018; Schoffelen and Gross, 2009). As signal mixing is essentially instantaneous, it is explicitly rejected in the calculation of GC, which estimates causal, and thereby time-delayed, interactions (Michalareas et al., 2016). The investigation of neuronal synchronization in the human brain is of the utmost importance, which has motivated the development of very advanced methods (Farahibozorg et al., 2018; Wang et al., 2018), some of which capitalize on the exclusion of instantaneous interactions (Colclough et al., 2015; Hipp et al., 2012; Nolte et al., 2004; Pascual-Marqui et al., 2017; Stam et al., 2007; Vinck et al., 2011).

These and related approaches in human participants link higher-order cognitive functions, including attention and working memory, to brain-wide networks synchronized at different frequency bands (Gross et al., 2002, 2004; Hipp et al., 2011; Kujala et al., 2007; Lobier et al., 2018; Rouhinen et al., 2020; Siegel

et al., 2008). Intrinsic brain networks in addition to task-related networks have been investigated with source-projected magnetoencephalography (MEG) and have revealed well-characterized resting-state networks through power correlation at different frequency bands (Brookes et al., 2011; de Pasquale et al., 2010; de Pasquale et al., 2012; Hipp et al., 2012; Hipp and Siegel, 2015). Source-projected human MEG has revealed intriguing relations between brain rhythms and anatomy. Source-projected MEG resting state recordings from 187 participants revealed dominant PFs across the cortex in the theta- to alpha-band range, decreasing along the posterior-anterior axis and negatively correlated to cortical thickness, a proxy of cortical hierarchical level (Mahjoory et al., 2020). Source-projected MEG data from participants performing an attention task on visual stimuli showed stimulus-induced occipital gamma-band activity with PFs that had a positive correlation, across 123 participants, with the local cortical thickness (van Pelt et al., 2018). Of particular relevance to the present study, source-projected MEG demonstrated that across 26 participants attentional top-down effects on alpha and gamma power in occipital cortex have a positive correlation to frontoparietal structural connectivity assessed with high angular resolution diffusion imaging magnetic resonance measurements (Marshall et al., 2015). Furthermore, alpha-band synchronization between superior-occipital cortex and the parietal lobule is modulated by attention, and its hemispheric asymmetry across 28 participants is predicted by the asymmetry in frontoparietal structural connectivity (D'Andrea et al., 2019).

Some of these studies capitalized on interindividual variability by performing correlation across many participants. By contrast, the typical approach in awake non-human primate research, due to economical constraints and ethical considerations, has been limited to two or so animals per study. This low N precludes cross-subject correlations and generally cross-subject statistical approaches, and it also limits inferences to the investigated sample, as in the present study (Fries and Maris, 2021). At the same time, chronic large-scale electrophysiological recordings in non-human primates provide coverage of many areas, although not as wide as MEG, with excellent spatial resolution and signal-to-noise ratio. This revealed that during a selective attention task, top-down GC from area 7A to V1 enhanced bottom-up GC from V1 to V4, and most strongly so when the top-down GC targeted the precise site from which the bottom-up GC originated (Richter et al., 2017). This result, in combination with the finding that occipito-parietal attention effects depend on frontoparietal structural connectivity (D'Andrea et al., 2019; Marshall et al., 2015), known to convey top-down influences, allows interesting predictions. The strength of top-down anatomical projections, assessed with FLNe, may predict the strength of attention effects at the top-down targets, assessed with mECoG. This is beyond the present study, but a fascinating topic for the future.

AC studies have shown that in non-human primates, the large range of cortical projection strengths, coupled with the EDR, results in the cortex being spatially embedded (Ercsey-Ravasz et al., 2013), so that the spatial pattern of long-distance connections is a defining feature of the cortical network (Horvát et al., 2016). Spatial embedding has been reported in humans and mice, indicating that it is a general characteristic of the cortex (Gămănuț et al., 2018; Horvát et al., 2016; Perinelli et al., 2019; Roberts et al., 2016; Rubinov et al., 2015). This leads to a high heterogeneity that is expressed structurally in a pronounced core-periphery organization (Markov et al., 2013). The present findings suggest that functional connectivity based on entrainment and synchronization shows a similarly high degree of heterogeneity, which is expressed in the modular organization found in the present study. Exploration of this functional heterogeneity promises to be a highly fruitful avenue for future research.

STAR★METHODS

Detailed methods are provided in the online version of this paper and include the following:

- KEY RESOURCES TABLE
- RESOURCE AVAILABILITY
  - Lead contact
  - Materials availability
  - Data and code availability
- EXPERIMENTAL MODEL AND SUBJECT DETAILS
- METHOD DETAILS
  - Visual attention task
  - Neurophysiological recordings
  - Data analysis
  - Volume registration of individual ECoG grids
  - Retrograde tracing database
- QUANTIFICATION AND STATISTICAL ANALYSIS

SUPPLEMENTAL INFORMATION

Supplemental information can be found online at https://doi.org/10.1016/j.neuron.2021.09.052.

ACKNOWLEDGMENTS

This work was supported by the DFG (SPP 1665 FR2557/1-1, FOR 1847 FR2557/2-1, FR2557/5-1-CORNET, FR2557/6-1-NeuroTMR, and FR2557/7-1-DualStreams, to P.F.), the EU (HEALTH-F2-2008-200728-BrainSynch, FP7-604102-HBP, and FP7-600730-Magnetrodes, to P.F.), a European Young Investigator Award (to P.F.), the National Institutes of Health (1U54MH091657-WU-Minn-Consortium-HCP, to P.F.), the LOEWE program (NeFF, to P.F.), the ANR (LABEX CORTEX ANR-11-LABX-0042, Universite de Lyon ANR-11-IDEX-0007, A2P2MC ANR-17-NENC-0004, CORTICITY ANR-17-HBPR-0003, REGION AUVERGNE-RHONE-ALPES SCUSI 1700933701, to H.K.), the ERC (Starting Grant SPATEMP, to M.V.), and the BMBF (BINDA, 031L0167, to M.V.). The authors would like to thank Olaf Sporns for thoughtful discussions and advice on modularity analysis, Jarrod Dowdall for helpful support at some stages of the analysis, and Georgios Spyropoulos for insightful discussions on earlier versions of the manuscript.

AUTHOR CONTRIBUTIONS

C.A.B. and P.F. designed the experiments and performed the implantation surgeries with the help of other colleagues; C.A.B. trained the monkeys and recorded the electrophysiological data; J.V., M.V., A.M.B., C.M.L., and C.A.B. wrote the analysis programs; J.V. performed the analyses with the help of M.V. and with the advice of P.F.; H.K. provided the anatomical data; J.V., M.V., H.K., and P.F. wrote the paper in collaboration with A.M.B., C.M.L., and C.A.B.

DECLARATION OF INTERESTS

P.F. and C.M.L. have a patent on thin-film electrodes and are beneficiaries of a respective license contract with Blackrock Microsystems LLC (Salt Lake City, UT, USA). P.F. is a member of the Scientific Technical Advisory Board of CorTec GmbH (Freiburg, Germany) and is managing director of Brain Science GmbH (Frankfurt am Main, Germany).

Received: December 16, 2020
Revised: August 10, 2021
Accepted: September 24, 2021
Published: October 20, 2021

REFERENCES

Baker, S.N., Olivier, E., and Lemon, R.N. (1997). Coherent oscillations in monkey motor cortex and hand muscle EMG show task-dependent modulation. J. Physiol. *501*, 225–241.

Barone, P., Batardiere, A., Knoblauch, K., and Kennedy, H. (2000). Laminar distribution of neurons in extrastriate areas projecting to visual areas V1 and V4 correlates with the hierarchical rank and indicates the operation of a distance rule. J. Neurosci. *20*, 3263–3281.

Bastos, A.M., Litvak, V., Moran, R., Bosman, C.A., Fries, P., and Friston, K.J. (2015a). A DCM study of spectral asymmetries in feedforward and feedback connections between visual areas V1 and V4 in the monkey. Neuroimage *108*, 460–475.

Bastos, A.M., Vezoli, J., Bosman, C.A., Schoffelen, J.M., Oostenveld, R., Dowdall, J.R., De Weerd, P., Kennedy, H., and Fries, P. (2015b). Visual areas exert feedforward and feedback influences through distinct frequency channels. Neuron *85*, 390–401.

Becker, R., and Hervais-Adelman, A. (2020). Resolving the Connectome, Spectrally-Specific Functional Connectivity Networks and Their Distinct Contributions to Behavior. eNeuro *7*, ENEURO.0101-20.2020.

Bosman, C.A., Schoffelen, J.M., Brunet, N., Oostenveld, R., Bastos, A.M., Womelsdorf, T., Rubehn, B., Stieglitz, T., De Weerd, P., and Fries, P. (2012). Attentional stimulus selection through selective synchronization between monkey visual areas. Neuron *75*, 875–888.

Brookes, M.J., Woolrich, M., Luckhoo, H., Price, D., Hale, J.R., Stephenson, M.C., Barnes, G.R., Smith, S.M., and Morris, P.G. (2011). Investigating the electrophysiological basis of resting state networks using magnetoencephalography. Proc. Natl. Acad. Sci. USA *108*, 16783–16788.

Brovelli, A., Ding, M., Ledberg, A., Chen, Y., Nakamura, R., and Bressler, S.L. (2004). Beta oscillations in a large-scale sensorimotor cortical network: directional influences revealed by Granger causality. Proc. Natl. Acad. Sci. USA *101*, 9849–9854.

Brunet, N.M., Bosman, C.A., Vinck, M., Roberts, M., Oostenveld, R., Desimone, R., De Weerd, P., and Fries, P. (2014). Stimulus repetition modulates gamma-band synchronization in primate visual cortex. Proc. Natl. Acad. Sci. USA *111*, 3626–3631.

Bruns, A., Eckhorn, R., Jokeit, H., and Ebner, A. (2000). Amplitude envelope correlation detects coupling among incoherent brain signals. Neuroreport *11*, 1509–1514.

Chaudhuri, R., Knoblauch, K., Gariel, M.A., Kennedy, H., and Wang, X.J. (2015). A Large-Scale Circuit Mechanism for Hierarchical Dynamical Processing in the Primate Cortex. Neuron *88*, 419–431.

Colclough, G.L., Brookes, M.J., Smith, S.M., and Woolrich, M.W. (2015). A symmetric multivariate leakage correction for MEG connectomes. Neuroimage *117*, 439–448.

D'Andrea, A., Chella, F., Marshall, T.R., Pizzella, V., Romani, G.L., Jensen, O., and Marzetti, L. (2019). Alpha and alpha-beta phase synchronization mediate the recruitment of the visuospatial attention network through the Superior Longitudinal Fasciculus. Neuroimage *188*, 722–732.

de Pasquale, F., Della Penna, S., Snyder, A.Z., Lewis, C., Mantini, D., Marzetti, L., Belardinelli, P., Ciancetta, L., Pizzella, V., Romani, G.L., and Corbetta, M. (2010). Temporal dynamics of spontaneous MEG activity in brain networks. Proc. Natl. Acad. Sci. USA *107*, 6040–6045.

de Pasquale, F., Della Penna, S., Snyder, A.Z., Marzetti, L., Pizzella, V., Romani, G.L., and Corbetta, M. (2012). A cortical core for dynamic integration of functional networks in the resting human brain. Neuron *74*, 753–764.

Dhamala, M., Rangarajan, G., and Ding, M. (2008). Estimating Granger causality from fourier and wavelet transforms of time series data. Phys. Rev. Lett. *100*, 018701.

Dodge, Y. (2008). The Concise Encyclopedia of Statistics (Springer).

Donahue, C.J., Sotiropoulos, S.N., Jbabdi, S., Hernandez-Fernandez, M., Behrens, T.E., Dyrby, T.B., Coalson, T., Kennedy, H., Knoblauch, K., Van Essen, D.C., and Glasser, M.F. (2016). Using Diffusion Tractography to Predict Cortical Connection Strength and Distance: A Quantitative Comparison with Tracers in the Monkey. J. Neurosci. *36*, 6758–6770.

Efron, B., and Tibshirani, R.J. (1994). An Introduction to the Bootstrap (CRC Press).

Ercsey-Ravasz, M., Markov, N.T., Lamy, C., Van Essen, D.C., Knoblauch, K., Toroczkai, Z., and Kennedy, H. (2013). A predictive network model of cerebral cortical connectivity based on a distance rule. Neuron *80*, 184–197.

Everitt, B.S., and Skrondal, A. (2008). The Cambridge Dictionary of Statistics (Cambridge University Press).

Fang, Q., and Boas, D.A. (2009). Tetrahedral mesh generation from volumetric binary and gray-scale images. In 2009 6th IEEE International Symposium on Biomedical Imaging: From Nano to Macro: Proceedings (Curran Associates).

Farahibozorg, S.R., Henson, R.N., and Hauk, O. (2018). Adaptive cortical parcellations for source reconstructed EEG/MEG connectomes. Neuroimage *169*, 23–45.

Fischer, F., Pieper, F., Galindo-Leon, E., Engler, G., Hilgetag, C.C., and Engel, A.K. (2018). Intrinsic Functional Connectivity Resembles Cortical Architecture at Various Levels of Isoflurane Anesthesia. Cereb. Cortex *28*, 2991–3003.

Forthofer, R.N., Lee, E.S., and Hernandez, M. (2008). Biostatistics: A Guide to Design, Analysis, and Discovery (Academic Press).

Fries, P., and Maris, E. (2021). What to do if N is two? arXiv, 2106.14562v1 http://arixiv.org/abs/2016.14562v1.

Gămănuţ, R., Kennedy, H., Toroczkai, Z., Ercsey-Ravasz, M., Van Essen, D.C., Knoblauch, K., and Burkhalter, A. (2018). The Mouse Cortical Connectome, Characterized by an Ultra-Dense Cortical Graph, Maintains Specificity by Distinct Connectivity Profiles. Neuron *97*, 698–715.e10.

Gregoriou, G.G., Gotts, S.J., Zhou, H., and Desimone, R. (2009). High-frequency, long-range coupling between prefrontal and visual cortex during attention. Science *324*, 1207–1210.

Gross, J., Timmermann, L., Kujala, J., Dirks, M., Schmitz, F., Salmelin, R., and Schnitzler, A. (2002). The neural basis of intermittent motor control in humans. Proc. Natl. Acad. Sci. USA *99*, 2299–2302.

Gross, J., Schmitz, F., Schnitzler, I., Kessler, K., Shapiro, K., Hommel, B., and Schnitzler, A. (2004). Modulation of long-range neural synchrony reflects temporal limitations of visual attention in humans. Proc. Natl. Acad. Sci. USA *101*, 13050–13055.

Grothe, I., Neitzel, S.D., Mandon, S., and Kreiter, A.K. (2012). Switching neuronal inputs by differential modulations of gamma-band phase-coherence. J. Neurosci. *32*, 16172–16180.

Guillery, R.W., and Sherman, S.M. (2002). Thalamic relay functions and their role in corticocortical communication: generalizations from the visual system. Neuron *33*, 163–175.

Hipp, J.F., and Siegel, M. (2015). BOLD fMRI Correlation Reflects Frequency-Specific Neuronal Correlation. Curr. Biol. *25*, 1368–1374.

Hipp, J.F., Engel, A.K., and Siegel, M. (2011). Oscillatory synchronization in large-scale cortical networks predicts perception. Neuron *69*, 387–396.

Hipp, J.F., Hawellek, D.J., Corbetta, M., Siegel, M., and Engel, A.K. (2012). Large-scale cortical correlation structure of spontaneous oscillatory activity. Nat. Neurosci. *15*, 884–890.

CellPress
OPEN ACCESS

Horvát, S., Gămănuţ, R., Ercsey-Ravasz, M., Magrou, L., Gămănuţ, B., Van Essen, D.C., Burkhalter, A., Knoblauch, K., Toroczkai, Z., and Kennedy, H. (2016). Spatial Embedding and Wiring Cost Constrain the Functional Layout of the Cortical Network of Rodents and Primates. PLoS Biol. *14*, e1002512.

Korn, E.L., Li, M.C., McShane, L.M., and Simon, R. (2007). An investigation of two multivariate permutation methods for controlling the false discovery proportion. Stat. Med. *26*, 4428–4440.

Kujala, J., Pammer, K., Cornelissen, P., Roebroeck, A., Formisano, E., and Salmelin, R. (2007). Phase coupling in a cerebro-cerebellar network at 8-13 Hz during reading. Cereb. Cortex *17*, 1476–1485.

Leopold, D.A., Murayama, Y., and Logothetis, N.K. (2003). Very slow activity fluctuations in monkey visual cortex: implications for functional brain imaging. Cereb. Cortex *13*, 422–433.

Lobier, M., Palva, J.M., and Palva, S. (2018). High-alpha band synchronization across frontal, parietal and visual cortex mediates behavioral and neuronal effects of visuospatial attention. Neuroimage *165*, 222–237.

Mahjoory, K., Schoffelen, J.M., Keitel, A., and Gross, J. (2020). The frequency gradient of human resting-state brain oscillations follows cortical hierarchies. eLife *9*, e53715.

Markov, N.T., Misery, P., Falchier, A., Lamy, C., Vezoli, J., Quilodran, R., Gariel, M.A., Giroud, P., Ercsey-Ravasz, M., Pilaz, L.J., et al. (2011). Weight consistency specifies regularities of macaque cortical networks. Cereb. Cortex *21*, 1254–1272.

Markov, N.T., Ercsey-Ravasz, M., Van Essen, D.C., Knoblauch, K., Toroczkai, Z., and Kennedy, H. (2013). Cortical high-density counterstream architectures. Science *342*, 1238406.

Markov, N.T., Ercsey-Ravasz, M.M., Ribeiro Gomes, A.R., Lamy, C., Magrou, L., Vezoli, J., Misery, P., Falchier, A., Quilodran, R., Gariel, M.A., et al. (2014a). A weighted and directed interareal connectivity matrix for macaque cerebral cortex. Cereb. Cortex *24*, 17–36.

Markov, N.T., Vezoli, J., Chameau, P., Falchier, A., Quilodran, R., Huissoud, C., Lamy, C., Misery, P., Giroud, P., Ullman, S., et al. (2014b). Anatomy of hierarchy: feedforward and feedback pathways in macaque visual cortex. J. Comp. Neurol. *522*, 225–259.

Marshall, T.R., Bergmann, T.O., and Jensen, O. (2015). Frontoparietal Structural Connectivity Mediates the Top-Down Control of Neuronal Synchronization Associated with Selective Attention. PLoS Biol. *13*, e1002272.

Michalareas, G., Vezoli, J., van Pelt, S., Schoffelen, J.M., Kennedy, H., and Fries, P. (2016). Alpha-Beta and Gamma Rhythms Subserve Feedback and Feedforward Influences among Human Visual Cortical Areas. Neuron *89*, 384–397.

Miller, E.K., Lundqvist, M., and Bastos, A.M. (2018). Working Memory 2.0. Neuron *100*, 463–475.

Mitra, P.P., and Pesaran, B. (1999). Analysis of dynamic brain imaging data. Biophys. J. *76*, 691–708.

Molnár, F., Horvát, S., Ribeiro Gomes, A.R., Ercsey-Ravasz, M., Knoblauch, K., Kennedy, H., and Toroczkai, Z. (2020). Predictability of cortico-cortical connections in the mammalian brain. bioRxiv. https://doi.org/10.1101/2020.12.03.410803.

Nelson, M.J., and Pouget, P. (2012). Physical model of coherent potentials measured with different electrode recording site sizes. J. Neurophysiol. *107*, 1291–1300.

Nolte, G., Bai, O., Wheaton, L., Mari, Z., Vorbach, S., and Hallett, M. (2004). Identifying true brain interaction from EEG data using the imaginary part of coherency. Clin. Neurophysiol. *115*, 2292–2307.

Oostenveld, R., Fries, P., Maris, E., and Schoffelen, J.M. (2011). FieldTrip: Open source software for advanced analysis of MEG, EEG, and invasive electrophysiological data. Comput. Intell. Neurosci. *2011*, 156869.

Palva, J.M., Wang, S.H., Palva, S., Zhigalov, A., Monto, S., Brookes, M.J., Schoffelen, J.M., and Jerbi, K. (2018). Ghost interactions in MEG/EEG source space: a note of caution on inter-areal coupling measures. Neuroimage *173*, 632–643.

Pascual-Marqui, R.D., Biscay, R.J., Bosch-Bayard, J., Faber, P., Kinoshita, T., Kochi, K., Milz, P., Nishida, K., and Yoshimura, M. (2017). Innovations orthogonalization: a solution to the major pitfalls of EEG/MEG "leakage correction.". https://www.researchgate.net/publication/319209855_Innovations_orthogonalization_a_solution_to_the_major_pitfalls_of_EEGMEG_leakage_correction.

Perinelli, A., Tabarelli, D., Miniussi, C., and Ricci, L. (2019). Dependence of connectivity on geometric distance in brain networks. Sci. Rep. *9*, 13412.

Pesaran, B., Vinck, M., Einevoll, G.T., Sirota, A., Fries, P., Siegel, M., Truccolo, W., Schroeder, C.E., and Srinivasan, R. (2018). Investigating large-scale brain dynamics using field potential recordings: analysis and interpretation. Nat. Neurosci. *21*, 903–919.

R Development Core Team (2013). R: A language and environment for statistical computing (R Foundation for Statistical Computing).

Richter, C.G., Thompson, W.H., Bosman, C.A., and Fries, P. (2017). Top-Down Beta Enhances Bottom-Up Gamma. J. Neurosci. *37*, 6698–6711.

Richter, C.G., Bosman, C.A., Vezoli, J., Schoffelen, J., and Fries, P. (2019). Brain rhythms shift and deploy attention. bioRxiv. https://doi.org/10.1101/795567.

Roberts, J.A., Perry, A., Lord, A.R., Roberts, G., Mitchell, P.B., Smith, R.E., Calamante, F., and Breakspear, M. (2016). The contribution of geometry to the human connectome. Neuroimage *124* (Pt A), 379–393.

Rohenkohl, G., Bosman, C.A., and Fries, P. (2018). Gamma Synchronization between V1 and V4 Improves Behavioral Performance. Neuron *100*, 953–963.e3.

Rohlfing, T., Kroenke, C.D., Sullivan, E.V., Dubach, M.F., Bowden, D.M., Grant, K.A., and Pfefferbaum, A. (2012). The INIA19 Template and NeuroMaps Atlas for Primate Brain Image Parcellation and Spatial Normalization. Front. Neuroinform. *6*, 27.

Rouhinen, S., Siebenhühner, F., Palva, J.M., and Palva, S. (2020). Spectral and Anatomical Patterns of Large-Scale Synchronization Predict Human Attentional Capacity. Cereb. Cortex *30*, 5293–5308.

Rubinov, M., and Sporns, O. (2010). Complex network measures of brain connectivity: uses and interpretations. Neuroimage *52*, 1059–1069.

Rubinov, M., Ypma, R.J., Watson, C., and Bullmore, E.T. (2015). Wiring cost and topological participation of the mouse brain connectome. Proc. Natl. Acad. Sci. USA *112*, 10032–10037.

Schoffelen, J.M., and Gross, J. (2009). Source connectivity analysis with MEG and EEG. Hum. Brain Mapp. *30*, 1857–1865.

Siegel, M., Donner, T.H., Oostenveld, R., Fries, P., and Engel, A.K. (2008). Neuronal synchronization along the dorsal visual pathway reflects the focus of spatial attention. Neuron *60*, 709–719.

Smith, S.M., Jenkinson, M., Woolrich, M.W., Beckmann, C.F., Behrens, T.E., Johansen-Berg, H., Bannister, P.R., De Luca, M., Drobnjak, I., Flitney, D.E., et al. (2004). Advances in functional and structural MR image analysis and implementation as FSL. Neuroimage *23* (*Suppl 1*), S208–S219.

Smith, S.M., Nichols, T.E., Vidaurre, D., Winkler, A.M., Behrens, T.E., Glasser, M.F., Ugurbil, K., Barch, D.M., Van Essen, D.C., and Miller, K.L. (2015). A positive-negative mode of population covariation links brain connectivity, demographics and behavior. Nat. Neurosci. *18*, 1565–1567.

Spyropoulos, G., Bosman, C.A., and Fries, P. (2018). A theta rhythm in macaque visual cortex and its attentional modulation. Proc. Natl. Acad. Sci. USA *115*, E5614–E5623.

Stam, C.J., Nolte, G., and Daffertshofer, A. (2007). Phase lag index: assessment of functional connectivity from multi channel EEG and MEG with diminished bias from common sources. Hum. Brain Mapp. *28*, 1178–1193.

Theodoni, P., Majka, P., Reser, D.H., Wójcik, D.K., Rosa, M.G.P., and Wang, X.J. (2021). Structural attributes and principles of the neocortical connectome in the marmoset monkey. Cereb. Cortex, bhab191.

Van Essen, D.C. (2012). Cortical cartography and Caret software. Neuroimage *62*, 757–764.

van Pelt, S., Shumskaya, E., and Fries, P. (2018). Cortical volume and sex influence visual gamma. Neuroimage *178*, 702–712.

Vezoli, J., Falchier, A., Jouve, B., Knoblauch, K., Young, M., and Kennedy, H. (2004). Quantitative analysis of connectivity in the visual cortex: extracting function from structure. Neuroscientist *10*, 476–482.

Vinck, M., Oostenveld, R., van Wingerden, M., Battaglia, F., and Pennartz, C.M. (2011). An improved index of phase-synchronization for electrophysiological data in the presence of volume-conduction, noise and sample-size bias. Neuroimage *55*, 1548–1565.

Wang, S.H., Lobier, M., Siebenhühner, F., Puoliväli, T., Palva, S., and Palva, J.M. (2018). Hyperedge bundling: a practical solution to spurious interactions in MEG/EEG source connectivity analyses. Neuroimage *173*, 610–622.

Williams, N., Wang, S.H., Arnulfo, G., Nobili, L., Palva, S., and Palva, J.M. (2021). Modules in connectomes of phase-synchronization comprise anatomically contiguous, functionally related regions. bioRxiv. https://doi.org/10.1101/2021.06.24.449415.

CellPress
OPEN ACCESS

STAR★METHODS

KEY RESOURCES TABLE

REAGENT or RESOURCE	SOURCE	IDENTIFIER
Deposited data		
Tract tracing dataset	Open Source	http://core-nets.org
Anatomical MRI template	Open Source	https://www.nitrc.org/projects/inia19/
F99 template and atlases	Open Source	https://balsa.wustl.edu/
FC spectra and matrices	This study	https://zenodo.org/record/5511890
Experimental models: organisms/strains		
M. mulatta.	German Primate Center	https://www.dpz.eu/en/home.html
Software and algorithms		
MATLAB	MathWorks	https://www.mathworks.com
FieldTrip	Open Source	https://www.fieldtriptoolbox.org
FSL	Open Source	https://fsl.fmrib.ox.ac.uk/fsl/fslwiki
iso2mesh	Open Source	http://iso2mesh.sf.net/cgi-bin/index.cgi
Brain Connectivity toolbox	Open Source	http://sites.google.com/site/bctnet/
R	R Development Core Team, 2013	https://www.R-project.org/
CORTEX	NIMH CORTEX	RRID:SCR_006837
Plexon	Plexon, USA	https://plexon.com
Neuralynx Digital Lynx system	Neuralynx, USA	https://neuralynx.com

RESOURCE AVAILABILITY

Lead contact

Further information and requests for resources should be directed to and will be fulfilled by the Lead Contact, Pascal Fries (pascal.fries@esi-frankfurt.de).

Materials availability

This study did not generate new unique reagents.

Data and code availability

The averaged functional connectivity spectra and the functional connectivity matrices of frequency-band averages for all pairs of recording sites are available at https://zenodo.org/record/5511890.

Code used for this study is freely available, and the respective references are reported below.

Any additional information required to reanalyze the data reported in this paper is available from the lead contact upon request.

EXPERIMENTAL MODEL AND SUBJECT DETAILS

All procedures were approved by the animal ethics committee of Radboud University (Nijmegen, the Netherlands). Data from two adult male Rhesus monkeys (Macaca mulatta) were used in this study.

METHOD DETAILS

Visual attention task

Stimuli and behavior were controlled by the software CORTEX (NIMH). After touching a bar, the acquisition of fixation, and a pre-stimulus *Baseline period* of 0.8 s, two isoluminant and isoeccentric stimuli (drifting sinusoidal gratings, diameter: 3 degrees, spatial frequency: ∼1 cycles/degree; drift velocity: ∼1 deg/s; resulting temporal frequency: ∼1 cycle/s; contrast: 100%) were presented on a CRT monitor (120 Hz refresh rate non-interlaced). In each trial, the light grating stripes of one stimulus were slightly tinted yellow, the stripes of the other stimulus were slightly tinted blue, assigned randomly (Figure 1A). After a variable *Pre-cue period* (1-1.5 s in Monkey 1, 0.8-1.3 s in Monkey 2), the color of the fixation point changed to blue or yellow, indicating the stimulus with the corresponding

color to be the behaviorally relevant one. Either one of the stimuli, irrespective of being cued or not, could change at a random time between stimulus onset and 4.5 s after cue onset. The period between cue onset and stimulus change is referred to as the *Post-cue period*. The stimulus change consisted of the stimulus' stripes undergoing a gentle bend, lasting 0.15 s. A trial was considered correct and the monkey was rewarded when the bar was released within 0.15-0.5 s of the change in the cued stimulus. No reward but a time-out was given when monkeys released the bar in response to equally likely changes of the non-cued stimulus. In Monkeys 1 and 2, 94% and 84% of bar releases, respectively, were correct reports of changes in the relevant stimulus. Trials were terminated without reward when the monkey released the bar outside the response window, or when it broke fixation (fixation window: 0.85 degree radius in Monkey 1, 1 degree radius in Monkey 2). Trials with attention directed to the stimulus in the visual hemifield contralateral to the recorded hemisphere are referred to as *Attended*, trials with attention ispilateral as *Unattended*. The analyses presented here pooled trials from those two attention conditions, unless otherwise specified, and they used only trials with correct behavioral report. The analyses used the period in the trial, during which stimuli were presented, and the monkey paid attention to one of them, i.e., the *Post-cue* period. The exception are Figures S5A, S7A, and S7B, which also used the *Baseline* period preceding stimulus onset (Bastos et al., 2015b). In total, the analyses used 9 sessions from Monkey 1 and 14 sessions from Monkey 2.

Neurophysiological recordings

Neuronal signals were recorded from the left hemisphere in two male rhesus monkeys using subdural ECoG grids consisting of 252 electrodes (1 mm diameter), which were spaced 2-3 mm apart. Two nearly identical copies of the ECoG grid were used in the two animals. Signals were amplified by eight 32-channel Plexon headstage amplifiers (Plexon, USA), against a silver wire implanted epidurally over the right occipital cortex (common recording reference). Signals were then high-pass (low-pass) filtered at 0.159Hz (8kHz) and digitized at approximately 32 kHz with a Digital Lynx acquisition system (Neuralynx, USA). Local Field Potentials were obtained by low-pass filtering at 250 Hz and down sampling to 1 kHz. Offline, the signals were re-referenced to remove the common recording reference through local bipolar derivations, i.e., sample-by-sample differences, between neighboring electrodes. Note that this procedure also allows rejection of headstage-specific noise and greater signal localization (Richter et al., 2019). Bipolar derivations were obtained for all pairs of immediately neighboring electrodes on the same lane of the ECoG grid, which were also recorded through the same headstage (Bastos et al., 2015b). We refer to bipolar derivations as "(recording) sites." The spatial position of each site was defined to be the midpoint between the two constituting electrodes. In both monkeys, the 252 electrodes resulted in 218 recordings sites. Site pairs with spectra indicative of artifactual coupling (broadband FC outliers, identified by visual inspection) were excluded from all analyses of all FC types: In monkey 1, this applied to 392 out of a total of 23.653 coherence and power correlation spectra (1.7%), and 784 out of a total of 47.306 GC spectra (1.7%); In monkey 2, this applied to 269 out of a total of 23.653 coherence and power correlation spectra (1.1%), and 538 out of a total of 47.306 GC spectra (1.1%). Power line artifacts at 50 Hz and its harmonics up to the Nyquist frequency, as well as screen refresh-rate artifacts (120Hz) were estimated and subtracted from the data using a Discrete Fourier Transform. In order to minimize volume conduction effects, we excluded site pairs with an inter-site distance (along the dural surface) of less than 4 mm from the calculation of interareal averages. Note that this corresponds to the diameter of an anatomical macrocolumn: Anatomical tract-tracing studies have shown that 95% of intrinsic connections are located within a distance of 1.9 mm centered on the injection site (Markov et al., 2011). Note that values of power correlation were very similar and highly correlated to values of orthogonalized power correlation (Figure S1C), used to exclude spurious coupling due to volume conduction (Hipp et al., 2012). Orthogonalized power correlation was computed with the FieldTrip function *ft_connectivity_powcorr_ortho,* excluding zero-lag contribution on a trial-by-trial basis.

Data analysis

All analyses were performed in MATLAB (MathWorks) using FieldTrip (https://www.fieldtriptoolbox.org) (Oostenveld et al., 2011) and custom scripts. Except otherwise noted, analyses used data recorded during the *Post-cue* period (as defined above, in the description of the visual attention task; see also Figure 1A). The first 0.3 s after cue onset were discarded to minimize cue-related transients. The remaining data until the first change of one of the stimuli (either target or distractor) were segmented into non-overlapping epochs of 1 s length. The exception to this are the analyses presented in Figures S5A, S7A, and S7B. These analyses include data from the *Baseline* period, which was merely 0.8 s long, and, after discarding 0.3 s of post-fixation transients, left merely 0.5 s of approximately stationary signals. To ease comparison, the *Post-cue* data shown in Figures S5A, S7A, and S7B were also segmented into non-overlapping 0.5 s epochs (again after discarding 0.3 s of cue-related transients). In total, this led to the following numbers of epochs and the following amounts of time. For the PostCue, we used 1565 (2067) epochs of 1sec, i.e., the total duration of data was 1565 s (2067 s) for Monkey 1 (Monkey 2). For the *Baseline*, we used 4239 (4396) epochs of 0.5 s, i.e., the total duration of data was 2119.5 s (2198 s) for Monkey 1 (Monkey 2). For the split Attention conditions, we used 1510 and 1358 (2540 and 2542) epochs of 0.5 s i.e., the total duration of data was 755 and 679 s (1270 and 1271 s), respectively, for the *Attended* and the *Unattended* conditions in Monkey 1 (Monkey 2).

Data epochs of 1 s length were multitapered using three Slepian tapers (Mitra and Pesaran, 1999) and Fourier-transformed (using the FieldTrip function "ft_freq_analysis" with the configuration option "mtmfft"), resulting in a spectral resolution of 1 Hz and a spectral smoothing of ± 1.5 Hz. Data epochs of 0.5 s length were multitapered using three Slepian tapers, zero-padded to 1 s, and Fourier-transformed (same FieldTrip approach), resulting in an interpolated spectral resolution of 1 Hz and a spectral smoothing of ± 3 Hz. The Fourier transforms were the basis for calculating the FC spectra, i.e., coherence spectra (Baker et al., 1997), spectra of power correlation across epochs (Bruns et al., 2000) and the GC spectra. These FC spectra were first calculated per

monkey, across all epochs of a given condition (*Post-cue, Attended, Unattended, Baseline*), and subsequently averaged over the two monkeys. Note that power correlation has frequently been calculated across partly overlapping windows, whereas we calculated power correlation across the non-overlapping epochs described above. GC spectra were estimated through non-parametric spectral matrix factorization of the cross-spectral density matrices, eliminating the need of explicit autoregressive data modeling with its inherent assumptions (Dhamala et al., 2008). For visualization only, FC spectra were smoothed with a frequency-dependent boxcar with a width of ± 1% of the respective center frequency.

We defined theta, beta, high-beta and gamma frequency bands, separately for each monkey, for each FC type, and for each task period (*Baseline* and *Post-cue*). The respective analyses used frequency bands that were specific per monkey, per FC type and per task period, yet averaged over site pairs and area pairs as detailed in the following. Per monkey, and per FC type, we first averaged FC spectra over all site pairs of a given area pair, separately for the 105 area pairs. To the resulting spectra, we applied an algorithm that blindly detected peaks and their corresponding peak frequencies (PFs) and full widths at half maximum (FWHM). PFs and FWHMs were similar for a given monkey across FC types. Yet, the two monkeys showed individual values, as expected from previous work (Rohenkohl et al., 2018; van Pelt et al., 2018). We report here PF and FWHM values averaged over area pairs and FC types, separately per monkey. Note that some area pairs showed spectra that lacked some of the peaks, and correspondingly did not contribute to the definition of the corresponding rhythms frequency band. For the *Post-cue* period, the theta rhythm was at 3 ± 2Hz (PF ± FWHM) in Monkey1, and 4 ± 3Hz in Monkey2; the beta rhythm was at 18 ± 5Hz in Monkey1 and 15 ± 5Hz in Monkey2; the high-beta rhythm was at 34 ± 5Hz in Monkey1 and 32 ± 4Hz in Monkey2; and the gamma rhythm was at 75 ± 8Hz in Monkey1 and 62 ± 8Hz in Monkey2 (Figures 1B, 1C, and S4A). In both monkeys, the theta, beta, high-beta and gamma band peaks were the only peaks detected. The same approach was applied for the *Baseline* period, and gave nearly identical results, even though only few site pairs showed a gamma peak during the *Baseline*. Analyses of FC in these four frequency bands averaged the respective FC metric over the frequency bins in the respective band.

Part of the same raw LFP data have been used in previous studies (Bastos et al., 2015a; Brunet et al., 2014; Richter et al., 2017; Rohenkohl et al., 2018; Spyropoulos et al., 2018). In particular, in one previous study, we used data from 8 visual areas, and focused our main analysis on the difference between bottom-up and top-down GC per area pair (Bastos et al., 2015b); this GC difference was related to the corresponding anatomical metric of the feedforward/feedback character of the projection, the SLN metric. The current study uses data from 15 brain areas, including visual and non-visual areas; it analyzes GC, coherence and power correlation and analyzes their full variability (not merely the area-wise GC difference) across all possible combinations of areas (and site pairs); this full FC variability is related to the corresponding anatomical metric of projection strengths, the FLNe metric.

Volume registration of individual ECoG grids

The anatomical MRI of each subject was spatially coregistered with the 3D positions of electrode locations using the FieldTrip toolbox (Oostenveld et al., 2011). These 3D positions were obtained by projecting the 2D positions (from high-resolution intraoperative photographs, using the sulci for alignment (Bastos et al., 2015b) onto each individual brain surface using the iso2mesh toolbox (Fang and Boas, 2009). Each individual anatomical MRI was coregistered, using linear and non-linear coregistration with FSL (Smith et al., 2004), to the F99 template brain containing anatomical atlases information (Van Essen, 2012) (Figure S1). This enabled us to assign each site to the underlying cortical area as done in (Bastos et al., 2015b), but here for all the 15 areas covered by ECoG grids (V1, V2, 8L, V4, TEO, DP, 8M, 7A, S1, TPt, 5, 7B, F1, F4 and F2). This resulted in the following numbers of sites per area in Monkey 1: V1: 24, V2: 9, V4: 17, DP: 10, TEO: 6, 8M: 6, 8L: 2, 7A: 7, S1: 20, 5: 13, TPt: 3, 7B, 20, F1: 23, F4: 4, F2: 17; and the following numbers of sites per area in Monkey 2: V1: 48, V2: 12, V4: 16, DP: 8, TEO: 3, 8M: 2, 8L: 3, 7A: 10, S1: 22, 5: 14, TPt: 2, 7B, 27, F1: 22, F4: 4, F2: 15. Furthermore, each individual anatomical MRI was aligned and warped to the INIA19 macaque brain template (Rohlfing et al., 2012), and the respective transformation matrix was then applied to a volume containing all ECoG electrode positions. This allowed us to combine the two ECoG grids on this template surface (Figure 1B) to create FC strength maps (Figures 2 and 3), after averaging overlapping parts of the two ECoG grids. Distances separating recording sites along the dural surface were determined with the fast-marching toolbox in MATLAB (MathWorks).

Retrograde tracing database

Acquisition and analysis of the anatomical dataset has been reported in (Chaudhuri et al., 2015; Markov et al., 2014a; Molnár et al., 2020). Updates, atlases and additional information concerning the anatomical dataset that was used for this work is available at http://core-nets.org. We used the fraction of labeled neurons (FLNe, defined in the Results section) to quantify AC strength. We used the proportion of supragranular labeled neurons (SLN, also defined in the Results section) to quantify the feedforward or feedback nature of an anatomical projection. Furthermore, we used interareal white-matter distances. For comparison of FC with AC, we selected areas and the corresponding site pairs of the ECoG grids, if they were also injected with retrograde tracers. This resulted in a total of 14 areas, which were electrophysiologically recorded in two macaques, and injected with tracers in a separate cohort of 26 macaques. The list of selected areas is: V1, V2, 8L, V4, TEO, DP, 8M, 7A, S1, 5, 7B, F1, F4, F2.

QUANTIFICATION AND STATISTICAL ANALYSIS

All statistical tests were based on the combined data from both animals with ECoGs, constituting a fixed-effect analysis that results in inferences limited to the investigated sample of two animals (Fries and Maris, 2021). To lend equal weight to each animal, data were first combined within each animal (across sites, site pairs, trials) and subsequently averaged over the two animals.

After definition of the four frequency bands per monkey, we tested which inter-areal site pairs showed significant FC, i.e., FC that reliably exceeded the bias level. This was done separately per FC type, i.e., power-correlation, coherence and GC. The bias level was estimated by randomly pairing epochs before FC calculation. For each of 100 randomizations, the maximum over all site-pairs was placed into a randomization distribution and site pairs were considered significant, if their FC exceeded the 97.5th percentiles of the randomization distribution (corresponding to a two-sided test).

Wherever possible, data from both monkeys were combined. The combined results amount to a fixed-effect analysis, allowing an inference on our sample of two animals, as in most other neurophysiology studies. Results presented are averages over interareal pairs and over all epochs. 99.9% confidence intervals were estimated from a bootstrap procedure over epochs as described in Efron and Tibshirani (1994): One-hundred bootstrap estimates of the mean were calculated for each area pair and each monkey, before averaging over monkeys. Averaging over monkeys was done after peak-alignment for each of the four frequency bands of interest.

The correlation between $\log_{10}$(FC) and $\log_{10}$(FLNe) was then performed and relevant statistics extracted, i.e., rho, p value and slope. We additionally extracted the FLNe-related FC change as the difference between FC values predicted (by linear regression) for minimal and maximal FLNe values. Due to the $\log_{10}$-transformation, the FLNe-related FC change reflects a fold change.

In order to investigate whether $\log_{10}$(FLNe) can be predicted by $\log_{10}$(FC) independently of distance, we performed a partial correlation in the form of a MR, according to the equation

$$\log_{10}(\mathrm{FLNe}) = b_1x_1 + b_2x_2 + \ldots + b_nx_n + b_dd + c,$$

with x_n being the $\log_{10}$(FC) for frequency band n, and d being the distance on the cortical surface (Figure 6) or through the white matter (Figure S6) (giving similar results, Table S2). The regression was calculated across area pairs, i.e., 182 area pairs for GC, and 91 area pairs for coherence and power correlation, as explained in the results section. FC values were first averaged for each area pair, over the corresponding site pairs, then over monkeys.

Note that the decay rates reported in the results were calculated using the natural logarithm as described in Ercsey-Ravasz et al. (2013).

By integrating distance into the regression model, we controlled for this potentially confounding variable and provide the partial correlation coefficients. However, in parallel to the expected bias reduction, the risk of data collinearity could in turn potentially reduce the precision of model estimates. Analyses revealed that all (log-transformed) FC metrics were linearly related to distance, leading to a multi-collinearity among the independent variables that may have affected the MR analysis. To investigate the severity of this, we performed several control analyses. First, we controlled for the non-violation of the ordinary least square assumption and plotted the residuals of the MR as a function of the predicted FLNe values separately for each FC metric (Figure S5C). This revealed no systematic relationships, i.e., no indication of relevant unobserved (hidden) variables. Second, we verified that variance inflation factors (VIFs) remained below critical levels, in particular for variables with significant model coefficients (Figure S5D). The VIF for a given predictor variable indicates the degree to which collinearity potentially inflates the standard error of its coefficient estimates, thereby reducing statistical power and warranting caution in the interpretation for this predictor. VIFs start at 1 meaning no correlation between predictor variables and any other; values between 2.5 and 5 indicate moderate correlation but not warranting corrective measures; values above 5 indicate a critical level (Dodge, 2008; Everitt and Skrondal, 2008). However, it is also important to note that values below 10 indicate that multicollinearity does not pose a serious problem to the MR model (Forthofer et al., 2008). We determined VIFs, separately per FC metric and frequency band (Figure S5D). These values were below the critical threshold for all combinations of FC metrics and frequency bands that had been found significantly predictive of FLNe in the previous analysis (Figure S5D). Third, we performed an analysis of structural coefficients and general dominance (Figures S7C–S7E). We computed squared structure coefficients (rs^2), general dominance (GenDom) and relative importance weights (RIW), as well as direct and shared effects for each variable, including distance (Figures S7C–S7E; Table S2). Importantly, even in the presence of correlation between variables, multicollinearity does not compromise the interpretation of MR coefficients provided this is done on grounds of outcome from analyses allowing assessment and control for collinearity, e.g., considering dominance or relative importance of partial regression coefficients. Hence, in addition to structure coefficients – measured already independently of collinearity and dividing each variable's contribution to the multiple regression effect – we measured direct effects of predictors and shared effects between predictors through 'unique' and 'common' coefficients calculated from commonality analysis (CA, performed with y-hat package under R, https://www.R-project.org/). For each predictor, the squared structure coefficients (rs^2) characterize the shared amount of variance with - or the individual contribution to - the multiple regression effect (R^2), and therefore should be interpreted as the amount of explained effect rather than explained variance of the dependent variable. In case of multicollinearity, CA provides the very useful direct and shared coefficients of total explained variance (R^2) to each subset of predictors from all possible subsets regression. It additionally allows identification of so-called 'suppressor' variables, through negative common coefficients, which estimate the amount of predictive power lost by other predictors when removing the considered variable(s) from the MR model. Direct or 'Unique' effects are comparable to change in the multiple coefficient of determination from squared semi-partial correlation after inclusion of a variable at last position of a hierarchical regression. Formulas for direct and shared components of a predictor variable Xi from a model with n predictors are, respectively Ui = -(1-Xi)XjXk… Xn) and Ci = -(1-Xi)(1-Xj)(1-Xk)…(1-Xn). Other relative importance measures considered and reported in Table S2 are Effect Size (for adjusted R^2), General Dominance weights (GenDom – average of overall conditional dominance weights i.e., additional contribution to multiple R^2 computed in all possible predictors combination comparisons) and Relative Importance Weights (RIW – proportional contribution to multiple R^2 after correcting correlation among

CellPress
OPEN ACCESS

predictors). Dominance analysis ranks predictors based on explained variance for all pairwise comparisons and minimizes the contribution of predictors in presence of collinearity. Thus, conclusions from GenDom and RIW should be consistent. Importantly, the sum of all weights equal the multiple R^2 of the MR model for both.

Similar to the principle of stepwise MR, we calculated the individual variables' contribution to the R^2 of the full-model by comparing the latter to the R^2 of the reduced model after removing this variable (Figures 6C, S6C, and S6F). We performed regression analyses for each of the 100 samples per predictor variable estimated from bootstrap over epochs, except for distance measures which do not change across trials.

Modularity analysis was performed using the latest version of the Brain Connectivity toolbox and the *modularity*, *agreement* and *consensus* functions (Rubinov and Sporns, 2010). For each of the three FC metrics separately, we computed a consensus community structure using the agreement matrix obtained from the concatenated degenerate partitions across the four frequency bands. This allowed to compare FC distributions between frequency bands, over the same set of modules (Figures 2 and S2). Degenerate partitions were obtained for each frequency band and FC metric by varying the resolution parameter between 0.1-10 (the classical resolution parameter value being 1, smaller values detecting larger modules and higher values detecting smaller modules). The consensus partition was computed with a re-clustering resolution of 0.25 (proportion of resolution parameters, for which any two vertices were assigned to the same class, across all four frequency bands), reapplied 100 times on the agreement matrix. The modularity values (q) reported on the margins of the matrices in Figures 2 and S2 were obtained with the classical resolution parameter of 1.

All violin plots use bootstrap estimates over trials, their shape along the y axis uses a kernel density estimate with a self-optimized bandwidth of the density kernel (https://github.com/bastibe/Violinplot-Matlab/blob/master/violinplot.m).

Statistical significance for average FC strength maps (Figure 3) was calculated by comparing experimentally obtained values for each site to values resulting from permutation of labels across sites i.e., the null model. This procedure was repeated 1000 times and corrected for multiple comparison, using a controlled false discovery rate of 20% with an alpha of 0.05 (Korn et al., 2007). By doing so, we compared the topography of frequency-specific maps to those obtained from a random graph with the same number of nodes, the same number of edges, and the same weight distribution i.e., same values as the original FC values.

Neuron, Volume *109*

Supplemental information

Brain rhythms define distinct interaction networks with differential dependence on anatomy

Julien Vezoli, Martin Vinck, Conrado Arturo Bosman, André Moraes Bastos, Christopher Murphy Lewis, Henry Kennedy, and Pascal Fries

Fig.S1

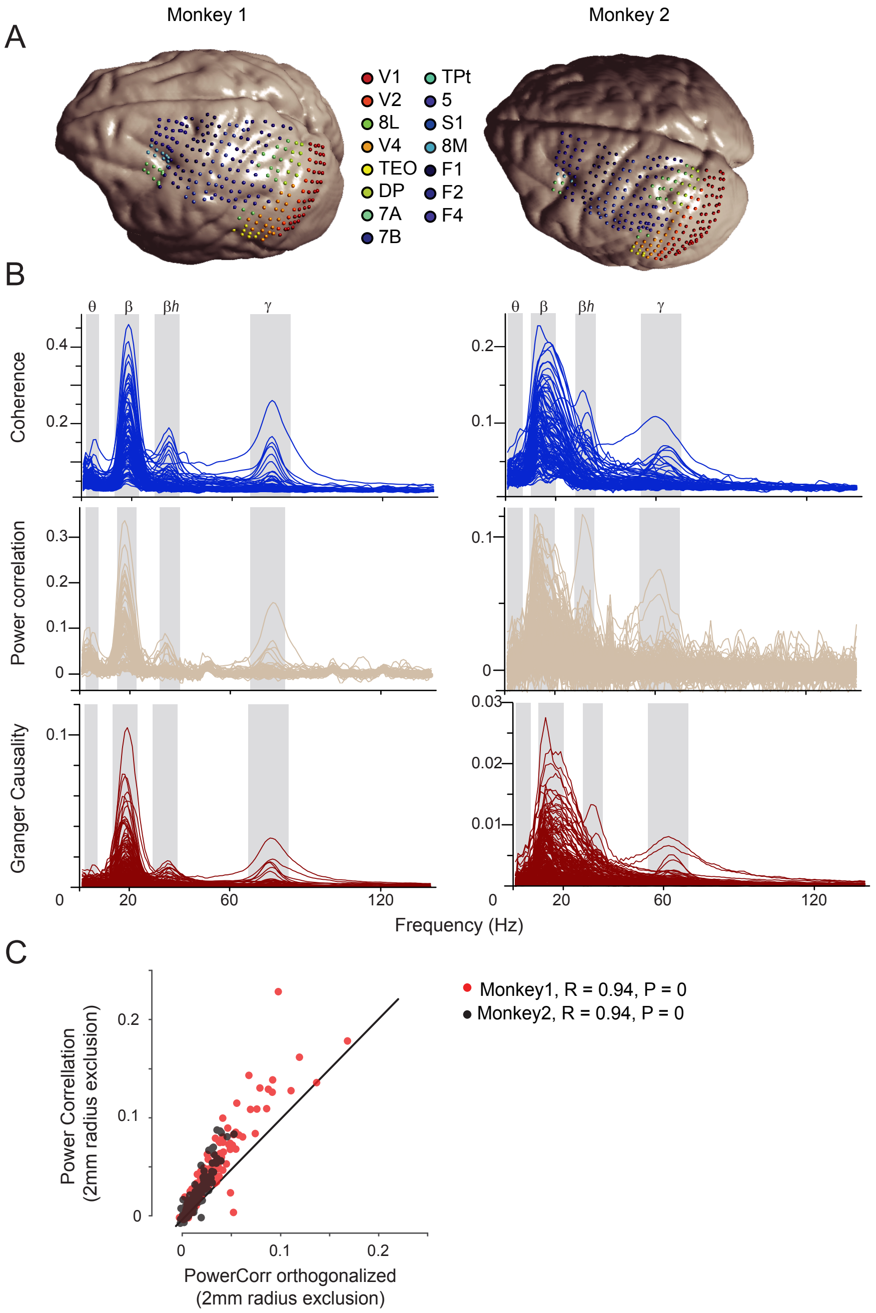

Figure S1. Recording site distributions and FC spectra per monkey, orthogonalized power correlation. Related to Figure 1.

(**A**) ECoG grids registered to brain surface reconstructions from individual MRIs of the two macaque monkeys, as indicated. Sites are colored according to the area color legend on the right, based on the Kennedy lab nomenclature (Markov et al., 2011). V1: Primary visual cortex; V2: Secondary visual cortex; 8L: Lateral part of area 8/FEF; V4: Fourth visual area; TEO: Temporal-occipital area; DP: Dorsal prelunate area; 7A and 7B: Parts A and B of parietal area 7; TPt: temporo-parietal area (posterior auditory association cortex); 5: area 5; S1: Primary somatosensory cortex; 8M: Medial part of area 8/FEF; F1: corresponding to Primary motor cortex; F2: corresponding to the caudal part of dorsal premotor cortex; F4: corresponding to the caudal part ventral premotor cortex.

(**B**) Same as Fig. 1C, but including power correlation and GC spectra. Spectra from Monkey 1 are in the left column, those of Monkey 2 in the right column. The coherence spectra in this figure are identical to those in Fig. 1C and merely included for completeness.

(**C**) Scatter plot of power correlation versus orthogonalized power correlation. Each dot corresponds to one pair of recording sites in Monkey 1 (red dots) or Monkey 2 (black dots), excluding sites within a 2 mm radius to avoid residual volume conduction effects. Note that power correlation and orthogonalized power correlation values were highly correlated (R=0.94, P=0) in both monkeys.

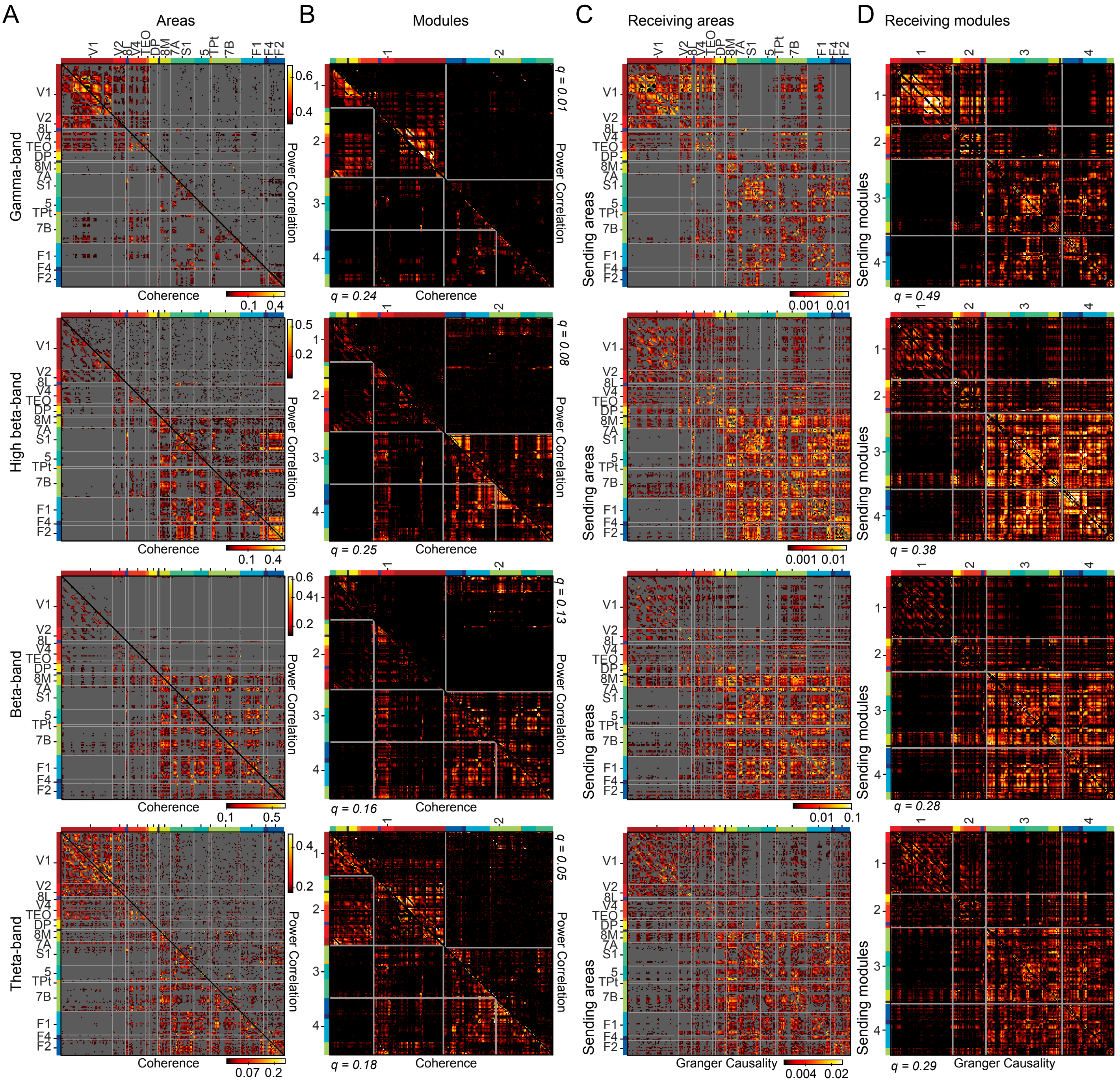

Figure S2. FC matrices for Monkey 2. Related to Figure 2.
(**A-D**) Same as Fig. 2 A-D, but for Monkey 2.

Fig.S3

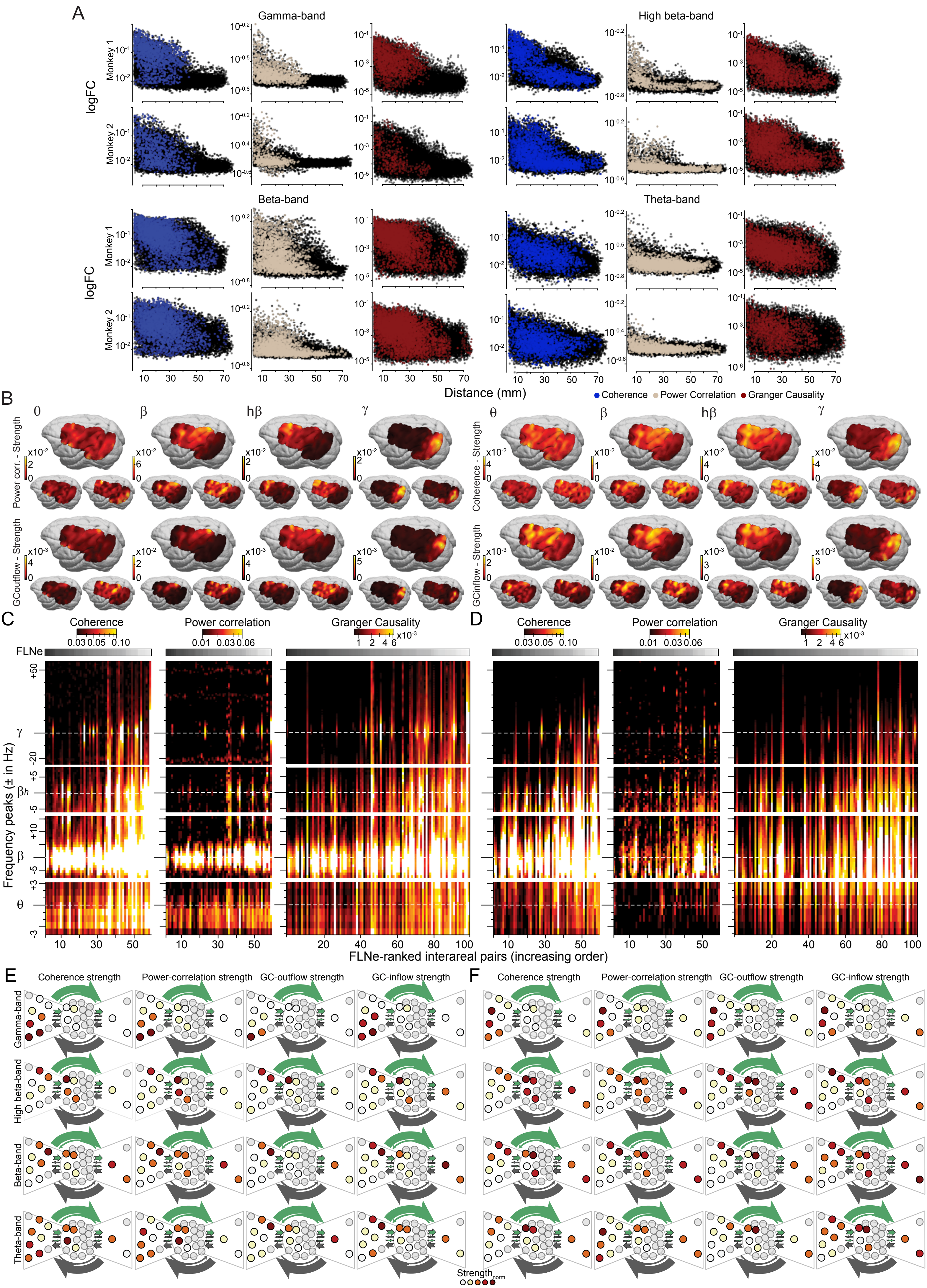

A
Gamma-band
High beta-band
Beta-band
Theta-band
logFC
Monkey 1
Monkey 2
Distance (mm)
Coherence
Power Correlation
Granger Causality
B
Power corr. - Strength
GCoutflow - Strength
Coherence - Strength
GCinflow - Strength
C
D
Coherence
Power correlation
Granger Causality
FLNe
Frequency peaks (± in Hz)
FLNe-ranked interareal pairs (increasing order)
E
F
Coherence strength
Power-correlation strength
GC-outflow strength
GC-inflow strength
Gamma-band
High beta-band
Beta-band
Theta-band
Strength

Figure S3. Per monkey: Site-pair-wise FC versus distance, FC strength topographies, FLNe-ranked FC spectra, and FC strength displayed on the AC-derived core-periphery structure. Related to Figure 3 and Figure 8.

(A) Scatter plots of FC versus distance on the cortical surface (each dot corresponds to one pair of recording sites from separate cortical areas), separately per FC type (color code on bottom right), per frequency band (as indicated above the four quadrants of the panel), and per monkey (as indicated for odd versus even rows). Black dots indicate lack of significance (comparison to a random graph with equal weight distribution; FDR-corrected for multiple comparisons over sites, see Methods).

(B) Same analyses as in Fig. 3, but showing data for the average over both monkeys (larger brains) and separately per monkey (smaller brains: Monkey 1 on left, Monkey 2 on right). To allow optimal comparison, significance masking was removed. Analyses were performed separately per FC type (as indicated to the left of the four quadrants of the panel), and per frequency band (as indicated on the upper left corner of each column).

(C) Same as left part of Fig. S4A, but for Monkey 1.

(D) Same as left part of Fig. S4A, but for Monkey 2.

(E) Same as Fig. 8, but for Monkey 1.

(F) Same as Fig. 8, but for Monkey 2.

Fig.S4

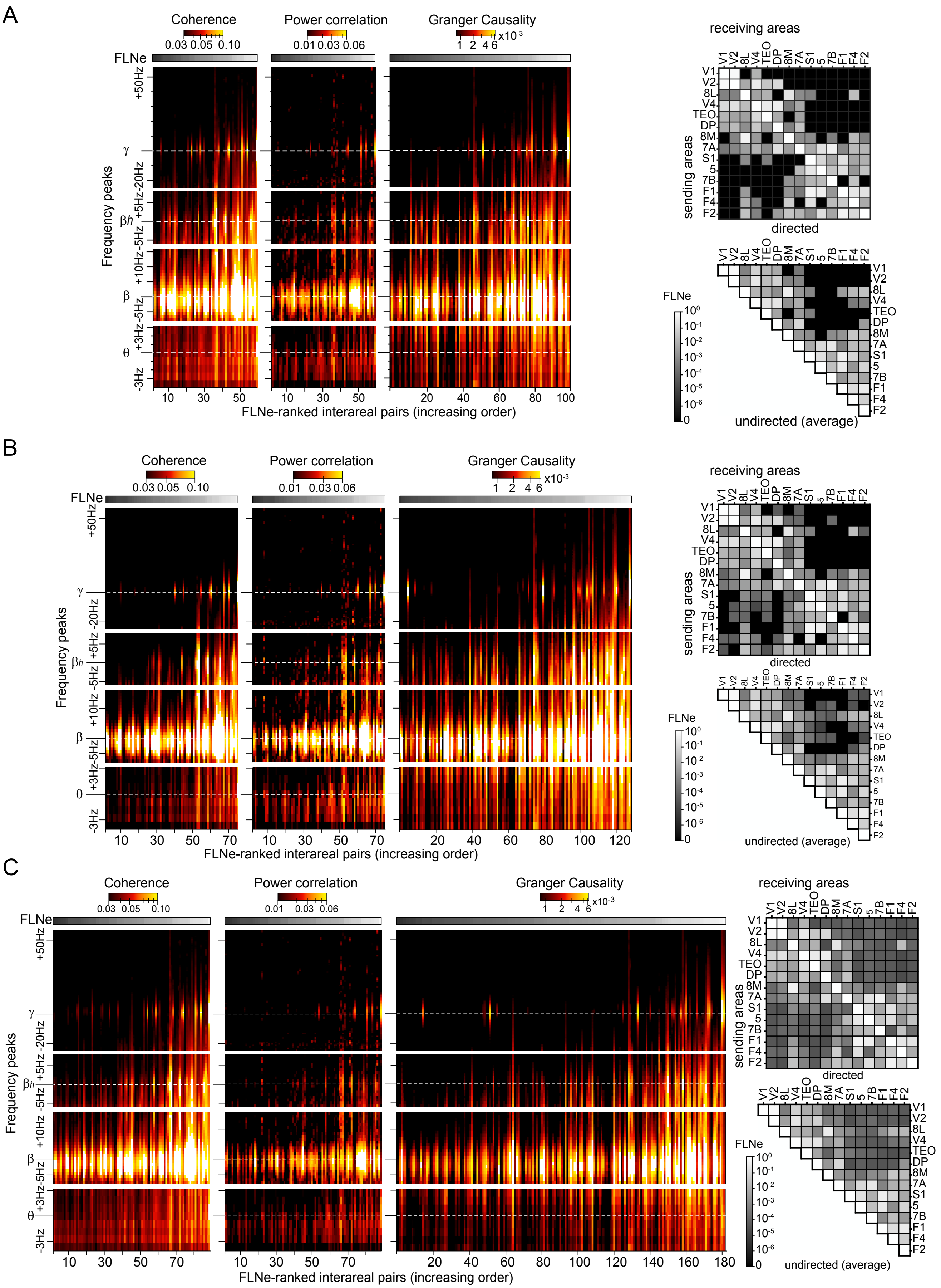

Figure S4. FLNe and FLNe-sorted FC spectra. Related to Figure 4.

(**A**) Coherence, power correlation and GC values (color coded) as a function of frequency (y-axis, relative to per-monkey band-wise peak frequencies) and as a function of FLNe rank (x-axis) of the respective interareal pair. For coherence and power correlation, ranking used FLNe averaged over the two directions as shown in the triangular matrix on the bottom right of the panel. For GC, ranking used FLNe as shown in the full matrix on the top right of the panel. FC values were first averaged over all respective interareal site pairs and subsequently over animals.

(**B**) Same as (A), but including FLNe values based on less than 10 neurons, while still excluding FLNe values of zero. The corresponding FLNe matrices are shown on the right.

(**C**) Same as (A), but including FLNe values based on less than 10 neurons, and replacing FLNe values of zero by estimates assuming Poisson distributions fitted to neuron counts from non-zero FLNe distributions. The corresponding FLNe matrices are shown on the right.

Fig.S5

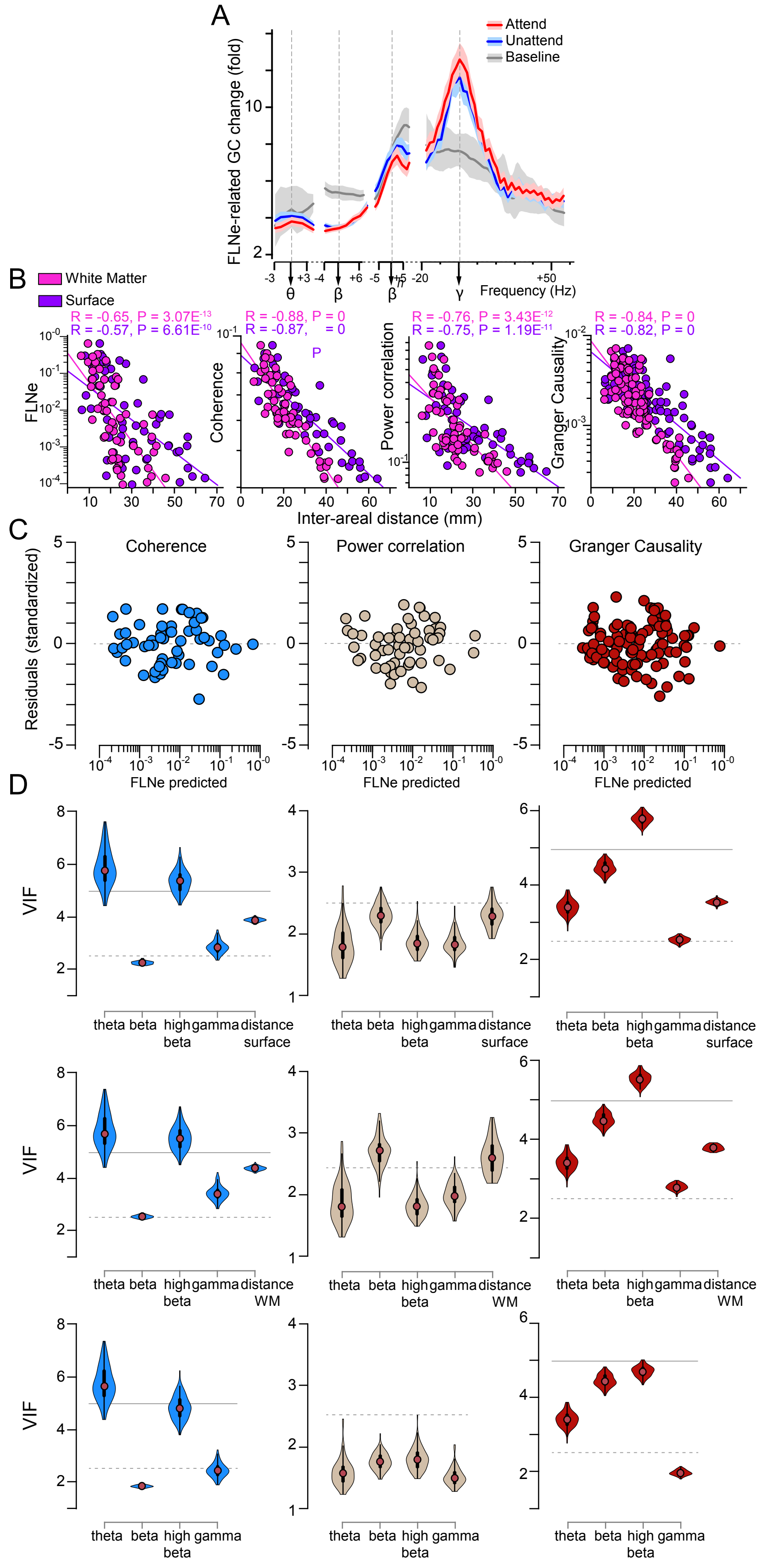

Figure S5. AC-FC correlation for attention conditions and baseline; AC and FC correlate with interareal distance. Related to Figure 5.

(**A**) Same as the FLNe-related GC change shown as red line in Fig. 5C, but here separately for the two attention conditions and the baseline period, as indicated in the color legend on the top right of the panel. Spectra show means over all trials ± 99.9% confidence intervals from bootstrap estimates over trials.

(**B**) Linear regression of log10(FLNe), log10(Coherence), log10(Power correlation) and log10(Granger Causality) against interareal distance on the brain surface (purple) or through white-matter (pink). For FLNe, each dot corresponds to an interareal projection with more than 10 labeled neurons (N=100), for coherence and power correlation, each dot corresponds to a pair of cortical areas (N=105), for GC, each dot corresponds to a directed interareal influence (N=210). FC values were averaged over the four individual frequency bands and over the two monkeys. Individual decay rates are presented in Table S1.

(**C**) Scatterplots of residuals against predicted variable verify homoscedasticity for each FC metric.

(**D**) Variance Inflation Factor (VIF) calculated for each predicting variable of the three models, with interareal distance on the brain surface (top) or through white-matter (WM, middle) as an additional predicting variable or not (bottom).

Dashed (VIF = 2.5) and plain lines (VIF = 5) show moderate correlation range still below critical level.

Fig.S6

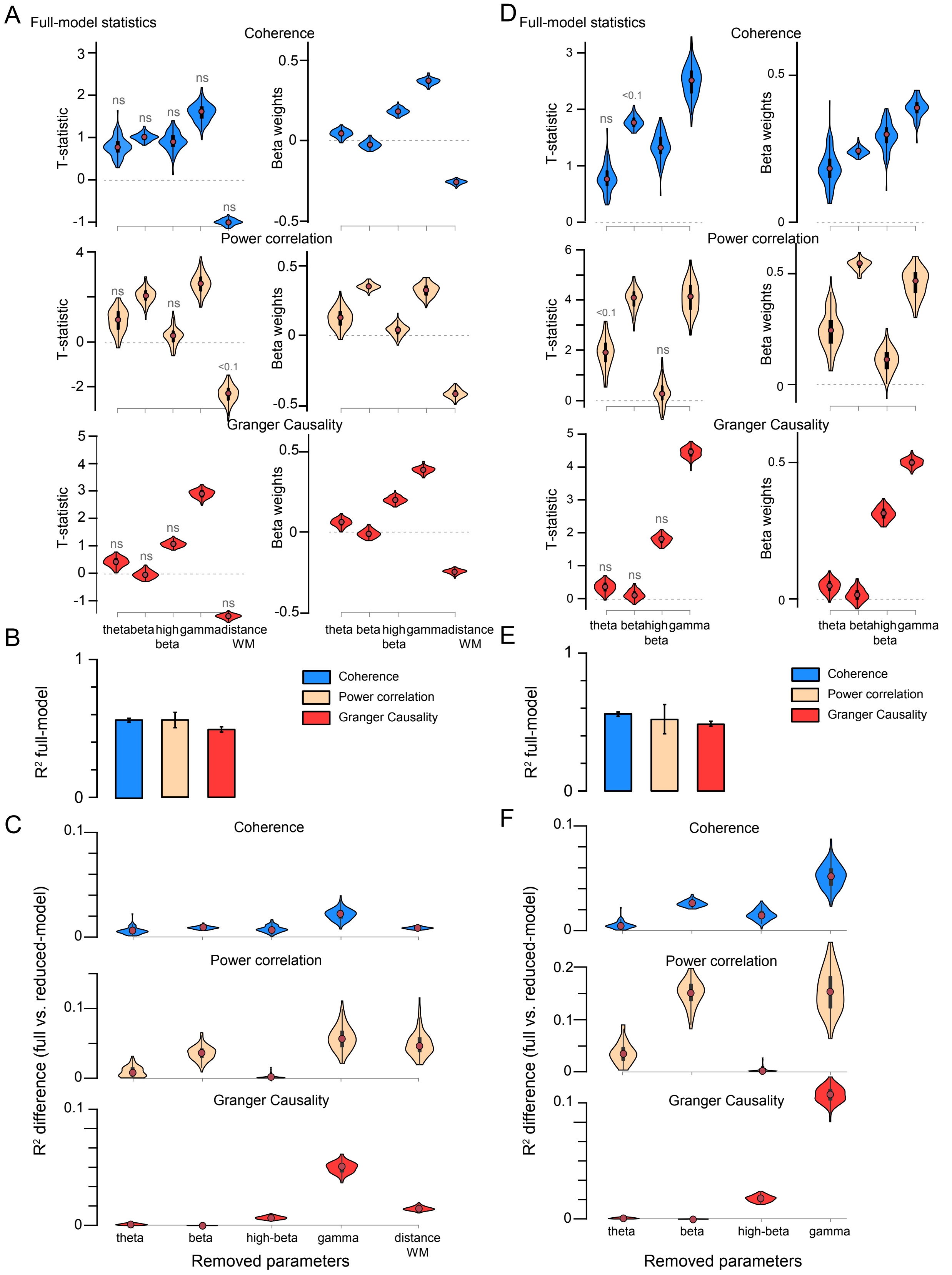

Figure S6. Multiple linear regression with or without interareal distance as additional predictor. Related to Figure 6.
(**A-C**) Same as Figure 6A-C, but using interareal distance through white-matter (WM).
(**D-F**) Same as Fig.6A-C, but not including interareal distance as predicting variable.
In (B) and (E), means and ± 99.9% confidence intervals from bootstrap estimates over trials.

Fig.S7

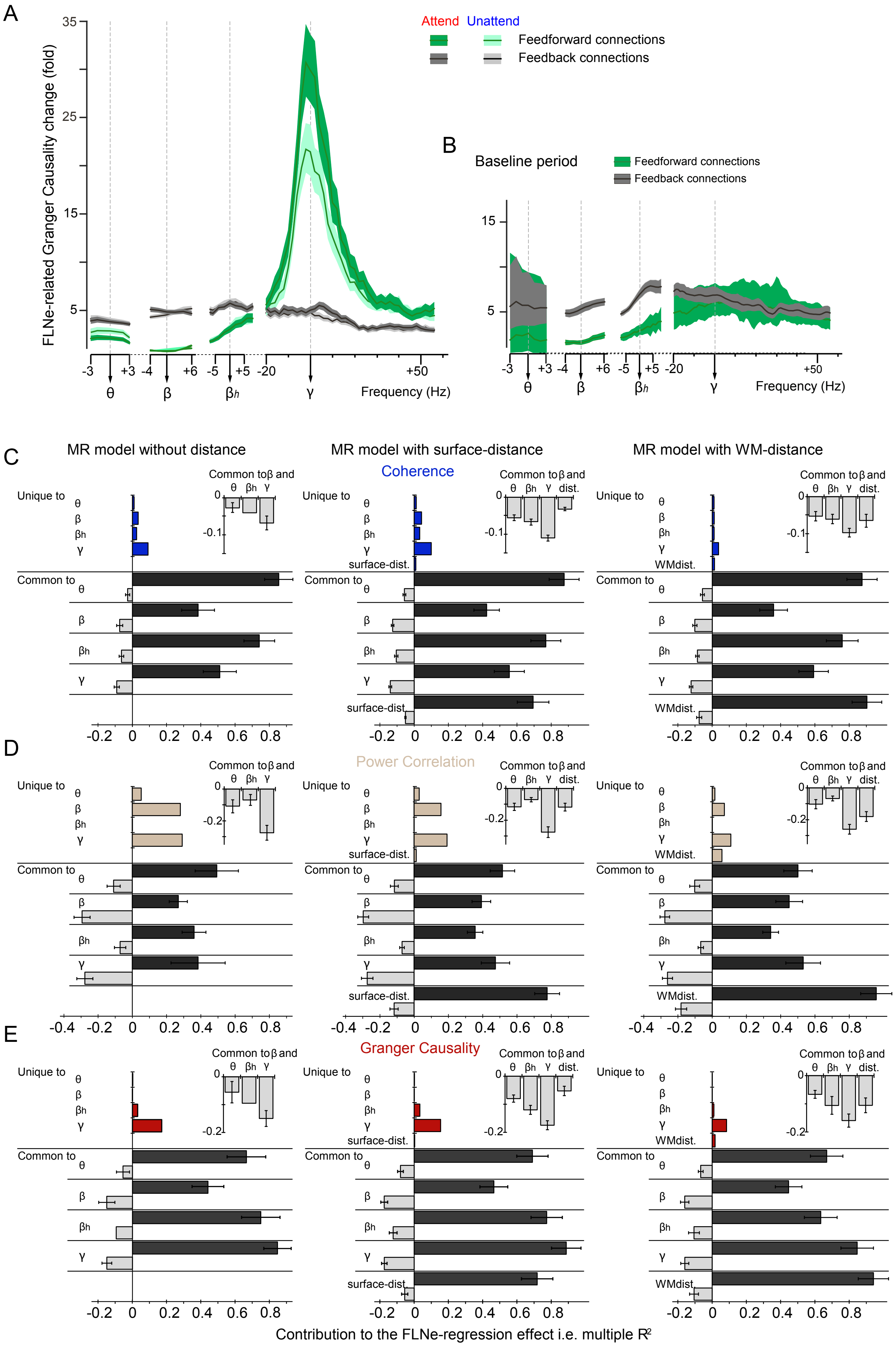
A
Attend Unattend
Feedforward connections
Feedback connections
FLNe-related Granger Causality change (fold)
Frequency (Hz)
B
Baseline period
Feedforward connections
Feedback connections
Frequency (Hz)
C
MR model without distance
MR model with surface-distance
MR model with WM-distance
Coherence
Unique to
Common to
Common to β and
surface-dist.
WMdist.
D
Power Correlation
E
Granger Causality
Contribution to the FLNe-regression effect i.e. multiple R²

Figure S7. AC-FC correlation split by SLN and attention conditions; Commonality analysis summary. Related to Figure 7.
(A) Same as Fig. 7A (main panel), but separately for the two attention conditions indicated at the top.
(B) Same as Fig. 7A (main panel), but for the Baseline period.
(C-E) Unique (*Direct*) and Common (*Shared*) effects for all predictor variables as proportion of the regression effect (i.e. multiple R^2) for MR models with and without distance as additional regressor and for the three FC metrics: Coherence (C), Power Correlations (D), Granger Causality (E). Common (*Shared*) effects are summed separately for positive and negative effects. Inset displays summed Common negative effects for all pairs of predictors involving beta-band synchronizations, Sum ± SD.
Results demonstrate that FC in the gamma band is the dominant variable, with the strongest direct effect and the highest relative importance in MR models predicting FLNe, with or without partial regression of distance. Commonality analysis identified a substantial proportion of the regression effect caused by suppression in MR models, consistently involving FC in the beta-frequency band (sum of negative shared coefficients for Coherence, Power Correlation and GC: 9.73%-29.4%-15.03% without distance, 17.64%-29.75%-17.79% with surface-distance and 12.97%-27.74%-15.98% with WM-distance). The amount of overall suppression caused by beta-band FC in the model was strongest when combined with FC in the gamma-frequency band (insets Fig.S7C-E) suggesting subtle involvement of cross-frequency interactions in the observed regression effect. Furthermore, in all models, we observed that beta-band FC contributed the least to the MR effect (i.e. lowest rs^2). Hence, even if poorly predictive of FLNe, the main contribution of beta-band FC to the MR effect was to increase FLNe-predictive power of the remaining variables and mostly for gamma-band FC.
Spectra in (A-B) show means over all trials ± 99.9% confidence intervals from bootstrap estimates over trials. (C-E) Mean ± SEM.

Fig.S8

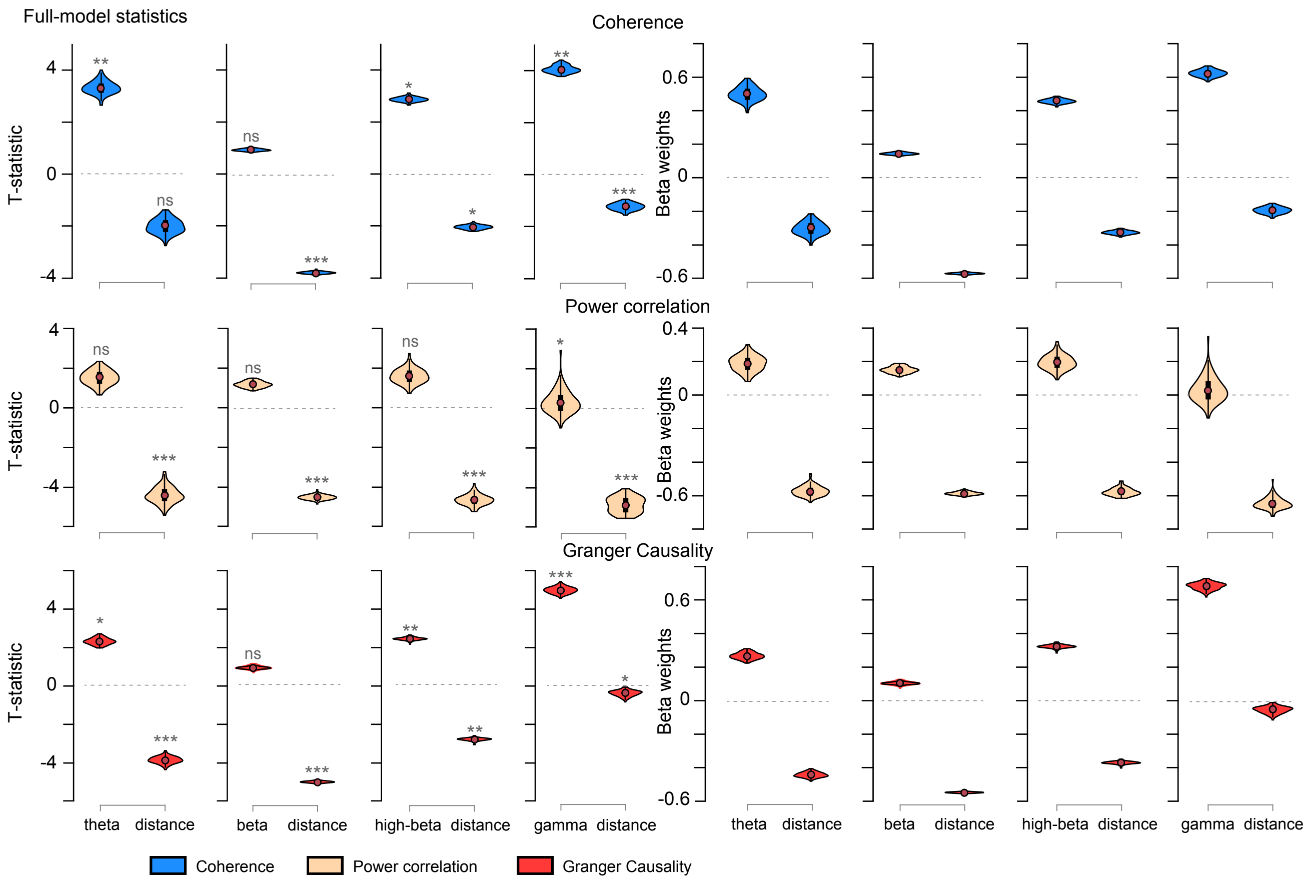

Figure S8. Multiple regression per frequency band. Related to Figure 6.
Similar analysis as Fig. 6, showing T-statistics and Beta weights of multiple linear regressions, with the dependent variable being log10(FLNe), and the independent variables being distance (on the cortical surface) and log10(FC), but now separately for theta, beta, high-beta and gamma, as indicated on the x-axes. The different FC types are shown in the four rows.

TableS1. Exponential decay rates of FC measures with interareal distance.

Mean over all trials ± SEM estimated from bootstrap over trials.

	Interareal distance	Coherence	Power Correlations	Granger Causality
Theta	WM	0.024 ± 0.0005 mm^{-1}	0.028 ± 0.004 mm^{-1}	0.055 ± 0.0012 mm^{-1}
	surface	0.016 ± 0.004 mm^{-1}	0.018 ± 0.003 mm^{-1}	0.034 ± 0.0008 mm^{-1}
Beta	WM	0.033 ± 0.0003 mm^{-1}	0.032 ± 0.001 mm^{-1}	0.063 ± 0.0007 mm^{-1}
	surface	0.021 ± 0.0002 mm^{-1}	0.019 ± 0.001 mm^{-1}	0.039 ± 0.0005 mm^{-1}
High-Beta	WM	0.042 ± 0.0004 mm^{-1}	0.028 ± 0.003 mm^{-1}	0.085 ± 0.0008 mm^{-1}
	surface	0.027 ± 0.0003 mm^{-1}	0.018 ± 0.002 mm^{-1}	0.055 ± 0.0005 mm^{-1}
Gamma	WM	0.027 ± 0.0004 mm^{-1}	0.015 ± 0.002 mm^{-1}	0.075 ± 0.0008 mm^{-1}
	surface	0.018 ± 0.0003 mm^{-1}	0.010 ± 0.001 mm^{-1}	0.049 ± 0.0006 mm^{-1}

TableS2. Summary statistics of multiple regression models for FLNe.

Relative importance measures (Commonality Analysis: Direct and Shared coefficients, General Dominance – GenDom., adjusted Effect Size and Relative Importance Weights – RIW) performed on the mean over all trials; otherwise, mean ± 99.9% CI estimated from bootstrap over trials.

Coherence

Predictors	R^2	R_{adj}^2	NRMSE	r_s^2	Beta values	Beta weights	sig.	Direct	Shared	GenDom.	Effect size	RIW
	0.563 ± 0.02	0.522 ± 0.02	0.23 ± 0.002									
Theta				0.83 ± 0.08	1.73 ± 1.65	0.20 ± 0.19	ns	0.0068	0.4606	0.153	0.136	0.138
Beta				0.34 ± 0.02	1.33 ± 0.26	0.28 ± 0.05	< 0.1	0.0241	0.1914	0.066	0.105	0.079
High-beta				0.69 ± 0.03	1.66 ± 0.75	0.34 ± 0.15	ns	0.0183	0.3919	0.125	0.203	0.123
Gamma				0.51 ± 0.04	2.23 ± 0.53	0.43 ± 0.10	0.0165	0.0562	0.2851	0.123	0.213	0.144
Surface dist.				0.65 ± 0.02	0.01 ± .003	0.17 ± 0.04	ns	0.0056	0.3678	0.096	-0.093	0.080
	0.569 ± 0.01	0.528 ± 0.01	0.23 ± 0.002									
Theta				0.83 ± 0.08	1.58 ± 1.59	0.19 ± 0.19	ns	0.0057	0.4618	0.144	0.123	0.133
Beta				0.34 ± 0.02	0.76 ± 0.22	0.16 ± 0.04	ns	0.0064	0.1850	0.057	0.057	0.068
High-beta				0.69 ± 0.03	1.04 ± 0.76	0.21 ± 0.15	ns	0.0061	0.3858	0.115	0.119	0.114
Gamma				0.50 ± 0.04	1.52 ± 0.57	0.29 ± 0.11	ns	0.0206	0.2645	0.104	0.141	0.123
WM dist.				0.84 ± 0.02	-0.02 ± .005	-0.21 ± 0.04	ns	0.0072	0.4674	0.146	0.126	0.128
	0.558 ± 0.01	0.525 ± 0.02	0.23 ± 0.002									
Theta				0.84 ± 0.08	1.56 ± 1.60	0.18 ± 0.19	ns	0.0055	0.4619	0.184	0.121	0.162
Beta				0.34 ± 0.02	-1.14 ± 0.22	0.24 ± 0.04	< 0.1	0.0188	0.1726	0.080	0.086	0.091
High-beta				0.70 ± 0.03	1.44 ± 0.75	0.29 ± 0.15	ns	0.0137	0.3782	0.152	0.166	0.146
Gamma				0.51 ± 0.04	2.02 ± 0.53	0.39 ± 0.10	0.02	0.0511	0.2340	0.142	0.186	0.160

Table S2 (continued). Summary statistics of multiple regression models for FLNe.
Mean over all trials ± 99.9% CI estimated from bootstrap over trials.

Power correlations

Predictors	R^2	R_{adj}^2	NRMSE	r_s^2	Beta values	Beta weights	sig.	Direct	Shared	GenDom.	Effect size	RIW
	0.545 ± 0.08	0.502 ± 0.08	0.23 ± 0.01									
Theta				0.43 ± 0.28	0.83 ± 1.21	0.19 ± 0.24	ns	0.0164	0.2193	0.092	0.086	0.094
Beta				0.25 ± 0.07	2.03 ± 0.75	0.49 ± 0.18	0.0028	0.0859	0.0525	0.107	0.167	0.118
High-beta				0.29 ± 0.18	0.05 ± 0.86	0.01 ± 0.20	ns	0.0001	0.1565	0.051	0.004	0.049
Gamma				0.39 ± 0.14	2.44 ± 1.06	0.49 ± 0.21	0.0011	0.1051	0.1103	0.149	0.210	0.159
Surface dist.				0.67 ± 0.10	-0.01 ± 0.01	-0.14 ± 0.18	ns	0.0070	0.3608	0.146	0.079	0.125
	0.571 ± 0.05	0.529 ± 0.06	0.23 ± 0.007									
Theta				0.41 ± 0.26	0.61 ± 1.15	0.14 ± 0.23	ns	0.0087	0.2770	0.086	0.063	0.089
Beta				0.24 ± 0.06	-1.50 ± 0.77	0.36 ± 0.18	0.0316	0.043	0.0981	0.089	0.124	0.100
High-beta				0.27 ± 0.17	0.11 ± 0.73	0.03 ± 0.17	ns	0.0004	0.1562	0.050	0.010	0.049
Gamma				0.38 ± 0.14	1.97 ± 1.00	0.40 ± 0.20	0.0085	0.0618	0.1536	0.129	0.170	0.139
WM dist.				0.83 ± 0.08	-0.03 ± 0.02	-0.32 ± 0.17	< 0.1	0.0321	0.4425	0.216	0.204	0.194
	0.539 ± 0.10	0.504 ± 0.11	0.23 ± 0.01									
Theta				0.39 ± 0.26	1.25 ± 1.32	0.24 ± 0.26	< 0.1	0.0281	0.2076	0.125	0.105	0.122
Beta				0.27 ± 0.08	2.34 ± 0.71	0.56 ± 0.16	0.0001	0.1516	-0.0132	0.151	0.191	0.155
High-beta				0.31 ± 0.19	0.15 ± 0.94	0.03 ± 0.22	ns	0.0003	0.1563	0.000	0.010	0.062
Gamma				0.36 ± 0.14	2.70 ± 1.11	0.55 ± 0.21	0.0001	0.1574	0.0580	0.157	0.233	0.199

Table S2 (continued). Summary statistics of multiple regression models for FLNe.
Mean over all trials ± 99.9% CI estimated from bootstrap over trials.

Granger Causality

Predictors	R^2	R_{adj}^2	NRMSE	r_s^2	Beta values	Beta weights	sig.	Direct	Shared	GenDom.	Effect size	RIW
	0.485 ± 0.02	0.458 ± 0.02	0.23 ± 0.02									
Theta				0.61 ± 0.07	0.24 ± 0.27	0.07 ± 0.07	ns	0.0009	0.2963	0.079	0.037	0.073
Beta				0.29 ± 0.03	-0.09 ± 0.17	-0.04 ± 0.07	ns	0.0002	0.1412	0.039	-0.014	0.039
High-beta				0.68 ± 0.03	0.78 ± 0.20	0.36 ± 0.08	< 0.1	0.0162	0.3162	0.100	0.191	0.090
Gamma				0.87 ± 0.03	1.17 ± 0.17	0.53 ± 0.06	0.0004	0.0751	0.3465	0.183	0.316	0.199
Surface dist.				0.67 ± 0.02	-0.01 ± 0.003	0.09 ± 0.05	ns	0.0016	0.3220	0.085	-0.045	0.084
	0.492 ± 0.01	0.465 ± 0.01	0.23 ± 0.001									
Theta				0.60 ± 0.07	0.19 ± 0.29	0.06 ± 0.08	ns	0.0005	0.2967	0.074	0.030	0.069
Beta				0.29 ± 0.02	-0.08 ± 0.17	-0.03 ± 0.07	ns	0.0002	0.1414	0.037	-0.012	0.036
High-beta				0.67 ± 0.03	0.44 ± 0.16	0.20 ± 0.06	ns	0.0052	0.3324	0.092	0.108	0.083
Gamma				0.86 ± 0.03	0.89 ± 0.17	0.40 ± 0.06	0.007	0.0414	0.4216	0.160	0.240	0.178
WM dist.				0.85 ± 0.02	-0.02 ± 0.004	-0.21 ± 0.04	ns	0.0086	0.4202	0.130	0.127	0.126
	0.484 ± 0.02	0.462 ± 0.02	0.23 ± 0.002									
Theta				0.61 ± 0.07	0.24 ± 0.27	0.07 ± 0.07	ns	0.0009	0.2963	0.097	0.038	0.087
Beta				0.29 ± 0.03	-0.09 ± 0.17	-0.04 ± 0.07	ns	0.0002	0.1412	0.048	-0.014	0.045
High-beta				0.69 ± 0.03	0.68 ± 0.19	0.31 ± 0.07	ns	0.0148	0.3176	0.125	0.165	0.111
Gamma				0.87 ± 0.03	1.09 ± 0.15	0.49 ± 0.05	0.0002	0.0831	0.3385	0.214	0.295	0.240
